# Association between *Helicobacter pylori* infection and arterial stiffness: Results from a large cross-sectional study

**DOI:** 10.1371/journal.pone.0221643

**Published:** 2019-08-29

**Authors:** Ji Min Choi, Seon Hee Lim, Yoo Min Han, Heesun Lee, Ji Yeon Seo, Hyo Eun Park, Min-Sun Kwak, Goh Eun Chung, Su-Yeon Choi, Joo Sung Kim

**Affiliations:** 1 Department of Internal Medicine, Healthcare Research Institute, Seoul National University Hospital Healthcare System Gangnam Center, Seoul, Republic of Korea; 2 Department of Internal Medicine and Liver Research Institute, Seoul National University College of Medicine, Seoul, Republic of Korea; Medical University Innsbruck, AUSTRIA

## Abstract

**Background:**

Chronic systemic inflammation is an important causative factor in the pathogenesis of atherosclerosis. However, the effect of chronic *Helicobacter pylori (Hp)* infection on arterial stiffness, a predictor of cardiovascular events, remains unclear. We evaluated the association between *Hp* infection and arterial stiffness in asymptomatic healthy individuals.

**Methods:**

Arterial stiffness was evaluated using the cardio-ankle vascular index (CAVI). We included subjects who underwent CAVI and anti-*Hp* IgG antibody evaluations, simultaneously, between March 2013 and July 2017. Demographic characteristics and metabolic and cardiovascular parameters were compared with respect to anti-*Hp* IgG antibody status. Multivariable logistic regression analyses were performed to determine the effect of *Hp*-seropositivity and conventional cardiovascular risk factors on arterial stiffness.

**Results:**

Of 2,251 subjects, 1,326 (58.9%) were included in the *Hp*-seropositive group. Median age (*P* < 0.001) and systolic blood pressure (*P* = 0.027) were significantly higher in the *Hp*-seropositive than in the *Hp*-seronegative group. Levels of LDL-cholesterol were significantly higher in the *Hp*-seropositive than in the *Hp*-seronegative group (*P* = 0.016). Other serum metabolic parameters were not significantly different between the two groups. The median CAVI value and the proportion of subjects with a CAVI ≥ 8 were significantly higher in the *Hp*-seropositive than in the *Hp*-seronegative group (both *P* < 0.001). On multivariable logistic regression analyses, *Hp*-seropositivity, age, body mass index, waist circumference, smoking, hypertension, diabetes mellitus, and dyslipidemia were significantly associated with high CAVI values. In the subgroup analysis conducted according to age group, a tendency towards an increased association between *Hp*-seropositivity and CAVI was observed with increasing age, even though the difference did not reach the statistical significance.

**Conclusions:**

*Hp*-seropositivity was significantly associated with arterial stiffness. *Hp* infection may contribute to the development of cardiovascular diseases.

## Introduction

*Helicobacter pylori (Hp)* is a Gram-negative, spiral-shaped bacterium that infects more than half of the world's population [1]. *Hp* plays a causative role in the development of many gastrointestinal diseases including chronic gastritis, peptic ulcers, gastric mucosa associated lymphoid tissue lymphoma [2], and gastric cancer [3]. Growing evidence has also supported a role for *Hp* infection in the pathogenesis of several extra-gastric diseases, including cardiovascular, neurological, hematological, and respiratory diseases and metabolic syndrome [4].

Atherosclerosis underlies the development of all cardiovascular diseases (CVDs), and inflammation plays an important role in the pathogenesis of atherosclerosis [5]. Studies have also investigated whether *Hp-*induced inflammation affects the development and progression of atherosclerosis. Although some epidemiological studies have shown a positive relationship between *Hp* infection and CVDs [6–10], others failed to find any association [11, 12]. In subjects with chronic *Hp* infection, levels of serum cytokines, including interleukin-6 and tumor necrotic factor-alpha, which are known to play a role in CVDs, are higher than in uninfected subjects [13, 14].

Arterial stiffness is an early marker of systemic atherosclerosis and an independent predictor of cardiovascular events and all-cause mortality [15, 16]. Arterial stiffness can be measured by several non-invasive methods [17]. Brachial-ankle pulse wave velocity (PWV) has been widely used to estimate arterial stiffness, but can be influenced by blood pressure (BP) at the time of measurement, thus limiting its routine clinical use [18]. Cardio-ankle vascular index (CAVI), a novel arterial stiffness index which represents the stiffness of the whole artery, is easy to measure, independent of BP, and has better reproducibility than PWV [18–20]. Therefore, CAVI has been used as a screening tool to assess subclinical atherosclerotic burden in asymptomatic healthy people [21].

This cross-sectional study was performed to investigate the association between *Hp* infection and arterial stiffness measured by CAVI in asymptomatic healthy subjects.

## Materials and methods

### Participants and study design

**Fig 1:**
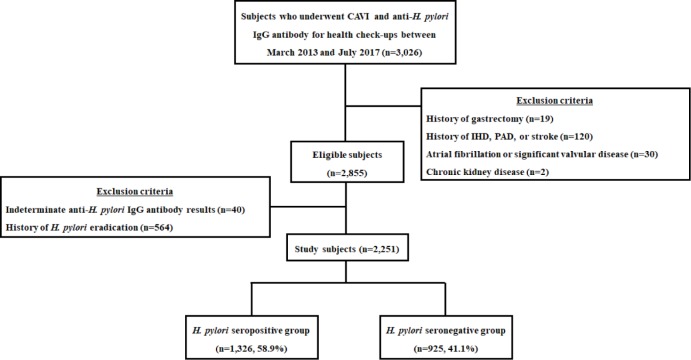
Study population. CAVI, cardio-ankle vascular index; *H*. *pylori*, *Helicobacter pylori*; n, number; IHD, ischemic heart disease; PAD, peripheral artery disease.

Fig 1 presents a schematic diagram of the study design. Between March 2013 and July 2017, subjects who underwent general health check-ups including CAVI and anti-*Hp* immunoglobulin G antibody (anti-*Hp* IgG) testing, simultaneously at Seoul National University Hospital Healthcare System Gangnam Center were enrolled in this retrospective cross-sectional study. All subjects were aged 18 years or older. Exclusion criteria were prior history of *Hp* eradication or gastrectomy, significant arrhythmia or valvular heart disease, ischemic heart disease, peripheral artery disease, stroke or chronic kidney disease [22]; and indeterminate anti-*Hp* IgG antibody results. After exclusion, the subjects were divided into two groups according to anti-*Hp* IgG antibody results: (1) *Hp*-seropositive group and (2) *Hp*-seronegative group. Baseline demographic data and laboratory markers including metabolic and cardiovascular parameters were obtained from subjects’ medical records. This study is reported according to the STROBE statement [23]. The study protocol was approved by the Ethics Committee of the Seoul National University Hospital (Institutional Review Board Number: H-1608-154-787) and was conducted in accordance with the Declaration of Helsinki. Written informed consent was obtained from each subject.

### Clinical, anthropometric and laboratory measurements

A structured self-reported questionnaire was used to access socio-demographic characteristics, medical history, current medications, and lifestyle factors such as smoking status, alcohol intake, and exercise [24]. Height and body weight were measured by digital scales. The body mass index (BMI) was calculated as body weight (in kilograms) divided by the squared height (in meters). Waist circumference (in centimeters) was evaluated by measuring at the midpoint between the lower costal margin and the iliac crest, using a non-stretch measuring tape. Smoking status was classified into two categories: never and ever (ex-smoker or current smoker). Excessive alcohol consumption was defined as drinking more than 140 g per week. Physical activity was classified into two categories: regular exercise (≥ 2 hours exercise a week, at least moderate intensity) or inactive group. BP was measured using sphygmomanometers in the seated position after 5 minutes rest. Hypertension was defined as 1) history of hypertension, 2) use of antihypertensive medication, or 3) systolic BP ≥ 140 mmHg and/or diastolic BP ≥ 90 mmHg. Diabetes mellitus was defined as 1) history of diabetes mellitus, 2) use of antidiabetic medications, or 3) fasting glucose level ≥ 126 mg/dL and/or glycosylated hemoglobin (HbA1c) ≥ 6.5%. Dyslipidemia was defined as 1) history of dyslipidemia, 2) use of antidyslipidemic medications, or 3) total cholesterol level ≥ 240 mg/dL and/or triglycerides ≥ 200 mg/dL and/or low-density lipoprotein (LDL) cholesterol ≥ 160 mg/dL and/or high density lipoprotein (HDL) cholesterol < 40 mg/dL.

For all subjects, blood was obtained after at least 10 hours of fasting, for the estimation of fasting blood glucose, HbA1c, renal function, and lipid profiles including total cholesterol, triglycerides, LDL and HDL cholesterol. Diagnosis of *Hp* infection was based on presence of serum anti-*Hp* IgG antibody tested using a commercially available immunoassay kit: HPG kit (Immulite® 2000 CMIA, Siemens, Germany). The HPG kit uses a chemiluminescent enzyme immunoassay, and has sensitivity and specificity of 91% and 100%, respectively [25]. Values higher than 1.10 IU/mL were considered positive [26]. To exclude false negative or positive results for anti-*Hp* IgG antibody, we reviewed serial changes of the titer in subjects who underwent multiple tests and referred to the results of rapid urease test or histologic examination of gastric tissue, if they were available. Approximately 51% (1,148/2,251) of all study subjects could be referred to their results of rapid urease test or histologic examination, but this reference had no effect on the grouping according to the *Hp* serostatus.

### Measurement of CAVI

To evaluate arterial stiffness, CAVI was calculated automatically with a VaSera VS-1000 (Fukuda Denshi, Tokyo, Japan) from the measurement of BP and pulse wave velocity, while monitoring the electrocardiogram and heart sounds [18, 19, 27]. After 5 minutes of rest, the brachial pulse pressure was measured using an automatic cuff oscillometric device in a seated subject. The average of two measurements was used for systolic and diastolic BP and pulse pressure. With the subject lying supine, cuffs were applied to both upper arms and ankles, a phonocardiogram was placed at the right sternal border in the second intercostal space, and electrocardiographic electrodes were attached to both wrists. The measurements were taken after a 10 minute rest period. PWV was calculated by dividing the distance from the aortic valve to the ankle by the sum of the time between the closing sound of the aortic valve and the notch of the brachial pulse wave, and the rise of the brachial pulse wave and the rise of ankle pulse wave. CAVI was obtained by using the following equation:

$$CAVI=a\left[(\frac{2\rho }{\Delta P})\times \ln(\frac{Ps}{Pd})\times {PWV}^{2}\right]+b$$

where *Ps* and *Pd* represent systolic and diastolic BP, respectively, *ΔP* is *Ps*-*Pd*, *ρ* is blood density, and *a* and *b* are constants. We used the mean value of the right and left CAVI for the analysis.

### Statistical analysis

Data were expressed as the median (interquartile range) or numbers and percentages. Categorical variables were analyzed by chi-square tests. Continuous variables were assessed by Mann-Whitney U test, a nonparametric test, because they were not distributed normally. A predefined CAVI cutoff value of 8 was used for further analysis, based on previous studies [28, 29]. A univariate analysis was performed to evaluate the associations between CAVI levels and *Hp* serologic status as well as conventional cardiovascular risk factors. All variables with *P* < 0.10 on univariate analysis and clinically relevant variables were included on a multivariable logistic regression model. Using multivariable logistic regression analyses, we assessed whether *Hp* seropositivity affects CAVI levels after adjusting for age, sex, and conventional cardiovascular risk factors. Odds ratios (ORs) and 95% confidence intervals (CIs) were used to estimate the association between the various factors and CAVI level. We performed subgroup analysis for the main outcome based on age. For subgroup analysis, we used multivariable analysis that included a test of interaction. The forest plot was used to demonstrate the difference in the effect of *Hp* infection on arterial stiffness according to subgroup, and the *P* value is for the interaction between *Hp* infection and age on arterial stiffness.

A *P* value <0.05 was considered statistically significant. Statistical analyses were performed using IBM SPSS 22.0 (IBM SPSS Statistics for Windows, Version 22.0, IBM Corp., Armonk, NY).

## Results

### Baseline characteristics

Between March 2013 and July 2017, 3,026 subjects underwent CAVI and anti-*Hp* IgG antibody testing. The baseline characteristics of 2,855 eligible subjects are shown in eligible subjects are shown in S1 Table. After exclusion of ineligible subjects, 2,251 subjects were included in the analysis: 1,326 (58.9%) subjects in the *Hp*-seropositive group and 925 (41.1%) subjects in the *Hp*-seronegative group (Fig 1). Baseline characteristics of the study subjects are shown in Table 1. Subjects in the *Hp*-seropositive group were significantly older than those in the *Hp*-seronegative group (*P* < 0.001). The proportion of ever smoker in the *Hp*-seropositive group was significantly higher than that in the *Hp*-seronegative group (*P* = 0.016). Median systolic BP was significantly higher in the *Hp*-seropositive group than the *Hp*-seronegative group (*P* = 0.027). There was no significant difference in sex composition, median BMI, median waist circumference, degree of alcohol consumption and physical activity, diastolic BP, pulse rate, or prevalence of hypertension and diabetes mellitus between the two groups. The prevalence of dyslipidemia in the *Hp*-seropositive group was significantly higher than that in the *Hp*-seronegative group (*P* = 0.040).

**Table 1:** Baseline characteristics of study subjects according to *H*. *pylori* seropositivity.

	*H*. *pylori* seropositive (n = 1,326)	*H*. *pylori* seronegative (n = 925)	*P* value
**Age, years**	55.0 (50.0–60.3)	53.0 (48.0–59.0)	<0.001
**Sex, male**	953 (71.9)	646 (69.8)	0.299
**BMI, kg/m^2^**	24.3 (22.4–26.2)	24.1 (22.3–26.0)	0.191
**Waist circumference, cm**	87.0 (82.0–93.0)	87.0 (81.0–92.0)	0.348
**Smoking status**			0.016
Never	685 (51.7)	526 (56.9)	
Ever*	641 (48.3)	399 (43.1)	
**Alcohol consumption**			0.658
Not excessive	995 (75.0)	686 (74.2)	
Excessive	331 (25.0)	239 (25.8)	
**Physical activity**			0.895
Regular exercise	499 (37.6)	351 (37.9)	
Inactive	827 (62.4)	574 (62.1)	
**Systolic BP, mmHg**	128.0 (119.0–138.0)	127.0 (118.0–137.0)	0.027
**Diastolic BP, mmHg**	84.0 (78.0–91.0)	84.0 (77.0–90.0)	0.229
**Pulse rate, bpm**	64.0 (58.0–70.0)	64.0 (58.0–71.0)	0.603
**Hypertension**	677 (51.1)	441 (47.7)	0.123
**Diabetes mellitus**	238 (17.9)	147 (15.9)	0.211
**Dyslipidemia**	706 (53.2)	451 (48.8)	0.040

Values are presented as median (interquartile range) or n (%). *H*. *pylori*, *Helicobacter pylori*; n, number; BMI, body mass index; BP, blood pressure

*: Ever smoker was defined as a current or ex-smoker.

### Laboratory markers including metabolic and cardiovascular parameters

As shown in Table 2, there were no significant differences in total cholesterol, triglyceride, HDL-cholesterol, fasting glucose, HbA1c, and parameters for renal function. LDL-cholesterol levels were significantly higher in the *Hp*-seropositive group than in the *Hp*-seronegative group.

**Table 2:** Laboratory markers including metabolic and cardiovascular parameters according to *H*. *pylori* seropositivity.

	*H*. *pylori* seropositive (n = 1,326)	*H*. *pylori* seronegative (n = 925)	*P* value
**Fasting glucose, mg/dL**	100.0 (92.0–110.0)	99.0 (92.0–109.0)	0.424
**Total cholesterol, mg/dL**	196.0 (173.0–221.0)	194.0 (170.5–217.0)	0.160
**Triglyceride, mg/dL**	105.0 (72.8–157.0)	110.0 (75.0–157.5)	0.341
**HDL-cholesterol, mg/dL**	52.5 (44.0–62.0)	53.0 (45.0–64.0)	0.202
**LDL- cholesterol, mg/dL**	123.0 (102.8–145.0)	119.0 (99.0–141.0)	0.016
**HbA1c, %**	5.6 (5.4–5.9)	5.6 (5.4–5.9)	0.277
**BUN, mg/dL**	14.0 (12.0–17.0)	14.0 (12.0–16.0)	0.433
**Creatinine, mg/dL**	0.9 (0.7–1.0)	0.9 (0.7–1.0)	0.180
**CAVI, right**	7.8 (7.1–8.6)	7.5 (7.0–8.2)	<0.001
**CAVI, left**	7.7 (7.1–8.4)	7.5 (7.0–8.1)	<0.001
**CAVI, mean**	7.8 (7.2–8.5)	7.6 (7.0–8.3)	<0.001
**CAVI ≥ 8.0**	533 (40.2)	282 (30.5)	<0.001

Values are presented as median (interquartile range) or n (%). *H*. *pylori*, *Helicobacter pylori*; n, number; HDL-cholesterol, high-density lipoprotein cholesterol; LDL-cholesterol, low-density lipoprotein cholesterol; HbA1c, glycosylated hemoglobin; BUN, blood urea nitrogen; CAVI, cardio-ankle vascular index

The overall distribution of CAVI values in both groups is shown in S1 Fig. In the *Hp*-seropositive group, the mean values of the right and left CAVI were higher than those of the *Hp*-seronegative group (*P* < 0.001). The percentage of subjects with a CAVI value higher than cutoff was significantly higher in the *Hp*-seropositive group than the *Hp*-seronegative group (*P* < 0.001).

### Factors associated with elevated CAVI levels

On univariate analysis, age, BMI, smoking status, hypertension, diabetes mellitus, dyslipidemia, and *Hp*-seropositivity were found to be significantly associated with elevated CAVI levels (Table 3). On multivariable logistic regression analysis, age (OR 1.15; 95% CI 1.13–1.17, *P* < 0.001), BMI (OR 0.80; 95% CI 0.74–0.86, *P* < 0.001), waist circumference (OR 1.04, 95% CI 1.02–1.07, *P* = 0.002), ever smoker (OR 1.96; 95% CI 1.58–2.43, *P* < 0.001), hypertension (OR 1.94; 95% CI 1.56–2.42, *P* < 0.001), diabetes mellitus (OR 1.66; 95% CI 1.26–2.19, *P* < 0.001), dyslipidemia (OR 1.36; 95% CI 1.10–1.68, *P* = 0.005), and *Hp*-seropositivity (OR 1.36; 95% CI 1.10–1.68, *P* = 0.005) were found to be associated with elevated CAVI levels. Sex, degree of alcohol consumption and physical activity were not significantly associated with elevated CAVI levels.

**Table 3:** Multivariate analysis of factors associated with elevated CAVI levels (CAVI≥ 8).

	Univariate analysis		Multivariate analysis^a^	
Risk factor	OR (95% CI)	*P* value	OR (95% CI)	*P* value
Age, years	1.16 (1.14–1.17)	<0.001	1.15 (1.13–1.17)	<0.001
Sex, male	1.03 (0.85–1.24)	0.767		
BMI, kg/m^2^	0.94 (0.92–0.97)	<0.001	0.80 (0.74–0.86)	<0.001
Waist circumference, cm	1.00 (0.99–1.01)	0.979	1.04 (1.02–1.07)	0.002
Smoking status, ever (vs. never)*	1.47 (1.23–1.74)	<0.001	1.96 (1.58–2.43)	<0.001
Alcohol, excessive (vs. not excessive)	1.05 (0.86–1.28)	0.641		
Physical activity, regular (vs. inactive)	0.87 (0.73–1.04)	0.130		
Hypertension	2.62 (2.19–3.13)	<0.001	1.94 (1.56–2.42)	<0.001
Diabetes mellitus	2.92 (2.34–3.66)	<0.001	1.66 (1.26–2.19)	<0.001
Dyslipidemia	1.79 (1.51–2.14)	<0.001	1.36 (1.10–1.68)	0.005
*Hp*-seropositivity	1.53 (1.28–1.83)	<0.001	1.36 (1.10–1.68)	0.005

CAVI, cardio-ankle vascular index; OR, odds ratio; CI, confidence interval; BMI, body mass index; *Hp*, *Helicobacter pylori*

*: Ever smoker was defined as a current or ex-smoker

^a^All variables with *P* <0.10 in univariate analysis and clinically relevant variables were included in a multivariate logistic regression model.

### Subgroup analysis of the effect of Hp-seropositivity on CAVI levels by age group

**Fig 2:**
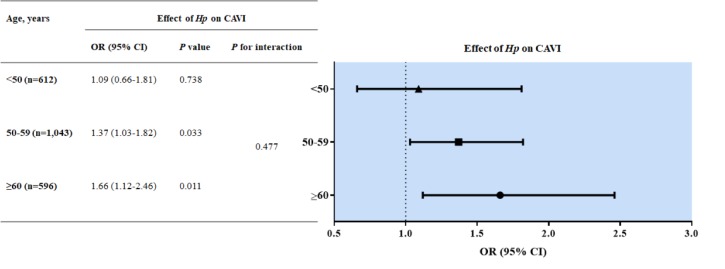
Subgroup analysis of the effect of *Hp*-seropositivity on CAVI levels by age group. When multivariable analysis was performed with variables affecting CAVI, the effect of *Hp*-seropositivity on CAVI levels increased with age. However, we did not find any significant interaction between age and *Hp* infection on arterial stiffness through a test of interaction. *Hp*, *Helicobacter pylori*; CAVI, cardio-ankle vascular index; n, number; OR, odds ratio; CI, confidence interval.

To investigate the effect of *Hp*-seropositivity on CAVI among patients of different ages, we divided the ages into three groups so that each group included approximately the same number of subjects (Fig 2; <50, 50–59 and ≥60 years). We found that the likelihood of *Hp*-seropositivity on CAVI levels increased with age: <50 years (adjusted OR 1.09; 95% CI 0.66–1.81, *P* = 0.738), 50–59 years (adjusted OR 1.37; 95% CI 1.03–1.82, *P* = 0.033), and ≥60 years (adjusted OR 1.66; 95% CI 1.12–2.46, *P* = 0.011). However, when we performed additional analysis, we did not find any significant interaction between *Hp* infection and age on arterial stiffness (*P* for interaction = 0.477, Fig 2).

## Discussion

In this cross-sectional study, we investigated the association between *Hp* infection and arterial stiffness measured by CAVI in asymptomatic healthy individuals. After adjusting for various cardiovascular risk factors, *Hp*-seropositivity showed significant association with increased arterial stiffness. Moreover, the effect of *Hp*-seropositivity on arterial stiffness was more pronounced with increasing age.

Many studies have investigated the association between *Hp* infection status and CVD. A recent meta-analysis found a significant association between *Hp* infection and risk of acute myocardial infarction [30]. Another longitudinal study revealed that *Hp* antibody levels predicted the incidence of stroke (OR 1.58; 95% CI 1.09–2.28) [8]. Studies have also reported that *Hp* infection affects the development of subclinical atherosclerosis, and is a potential risk factor for the development of CVD in healthy populations [9, 10, 31]. In these studies, indicators of subclinical atherosclerosis such as PWV, calcium score, or degree of coronary artery stenosis were worse in the *Hp*-infected group than in the non-infected group. However, other studies failed to show a positive association between *Hp* infection and CVD [12, 16, 32, 33]. Alternatively, some studies showed that CVD is only related to some virulent *Hp* strains, such as those carrying the cytotoxin associated gene-A antigen, suggesting that the effects of *Hp* on CVD may depend upon *Hp* virulence factors [34–36]. In this study, we used CAVI to evaluate arterial stiffness, an indicator of subclinical atherosclerosis, in a large number of healthy subjects, and demonstrated a significant association between *Hp* infection and subclinical atherosclerosis.

Previous studies demonstrated that CAVI ≥ 8 was associated with significant coronary stenosis or calcification in asymptomatic subjects [28, 29] and is the optimal cutoff for predicting carotid arteriosclerosis [37]. In this study, we also analyzed the factors associated with arterial stiffness based on a CAVI value of 8 (< or ≥8). On multivariable analysis, age was a significant predictor of CAVI, consistent with the results of previous studies [38], whereas sex was not. The mean age of subjects included in this study (54.65±9.03) was higher than that of previous studies (47.1±12.5) in which sex was significantly associated with CAVI values [38, 39]. This discrepancy may be explained by the older age of the subjects in this study [38, 40].

BMI was negatively correlated with CAVI in this study. Some studies that have examined the effect of body fat on CAVI demonstrated that increased BMI, which can be interpreted as the systemic accumulation of fat, has a negative correlation with CAVI, as in this study [38, 39]. Although multiple hypotheses have been proposed to explain these inverse correlations [41], other studies have suggested that visceral obesity, rather than overall obesity per se, is a significant risk factor for increased arterial stiffness [42, 43]. Alternatively, this study failed to show a significant association of waist circumference, an index of central obesity, with CAVI in univariate analysis. However, given that waist circumference is a clinically relevant parameter, it was included as one of the variables in multivariate analysis. As a result, waist circumference was also significantly correlated with CAVI after adjusting for other variables. Other well-known risk factors for CVD, such as smoking status, hypertension, diabetes mellitus, and dyslipidemia, have also been shown to have a strong effect on CAVI levels [38, 39].

In the present study, *Hp*-seropositivity was an independent predictor of arterial stiffness. However, the mechanism whereby *Hp* infection induces atherosclerosis is unclear. Some studies have suggested that *Hp* infection may induce dyslipidemia, including an increase in total cholesterol, LDL-cholesterol and triglyceride and decrease in HDL-cholesterol, resulting in CVD [44, 45]. In our study, the level of LDL-cholesterol and the prevalence of dyslipidemia was also significantly higher in the *Hp*-seropositive group. Chronic *Hp* infection has also been reported to trigger a T1 helper cell mediated inflammatory reaction and release of inflammatory cytokines such as interleukin-1, 6, 8 and tumor necrotic factor-alpha, which lead to endothelial dysfunction [13, 14]. Furthermore, it has been reported that malabsorption of vitamin B12 and folic acid can contribute to the development of atherosclerosis by inducing hyperhomocysteinemia [46]. On the other hand, some studies suggest that *Hp* may be directly related to atherosclerotic plaque formation, as supported by the detection of *Hp* DNA in atherosclerotic plaques [47, 48]. Nevertheless, further research is required to elucidate the association between chronic *Hp* infection and the formation and progression of atherosclerosis.

We investigated whether the effect of *Hp* infection on CAVI levels varied by age (Fig 2). When divided into three subgroups, the effect of *Hp*-seropositivity on CAVI increased with age (<50 years, adjusted OR 1.09, 95% CI 0.66–1.81; 50–59 years, adjusted OR 1.37; 95% CI 1.03–1.82; ≥60 years, adjusted OR 1.66; 95% CI 1.12–2.46). Even though we failed to demonstrate the significant interaction between age and *Hp* infection on arterial stiffness (*P* for interaction = 0.477), *Hp* seropositivity tended to have a greater impact on arterial stiffness with age. Considering that most *Hp* infections are acquired during early childhood and last for many years or even lifelong if untreated, older age groups would be assumed to have long-standing *Hp* infection [49]. Our data demonstrated that the longer the infection period, the greater the influence of the *Hp* infection on the CAVI.

Our study has several strengths. First, a large number of subjects were included in the analysis, thus making the results robust. Second, medical staff obtained accurate information regarding patients’ past medical history, medication history, and history of *Hp* eradication. In addition, we measured arterial stiffness using CAVI, a highly reproducible technique that is unaffected by BP, thus allowing us to obtain accurate and reliable results. Furthermore, to the best of our knowledge, this is the first study to demonstrate that the effects of *Hp* infection on arterial stiffness may be influenced by patient age, thus suggesting an association with the duration of the *Hp* infection.

This study also has some limitations. First, this was a cross-sectional study, and therefore, causation could not be established. Furthermore, the influence of *Hp* infection on the progression of arterial stiffness over time cannot be deduced. Second, this study did not include virulent strains of *Hp*. It is known that the effect of *Hp* infection on the development of certain diseases is different depending on the virulence factor; therefore, additional studies that take this into account are required. Third, *Hp* infection was diagnosed using noninvasive serological tests. This diagnostic method is inexpensive, fast, and widely available, making it the most commonly used in a mass investigation. Although serologic tests have a high negative predictive value, they cannot reliably distinguish between current and past infections. In other words, *Hp* serology may remain positive for several years even after successful *Hp* eradication [50]. To overcome this limitation, we thoroughly investigated the history of *Hp* eradication therapy and tried to improve the reliability of the study by supplementing the shortcomings of serologic tests. Lastly, we only included subjects who underwent CAVI. In our institution, the physician proposes an investigation plan for each asymptomatic subject after taking into account individual comorbidities (hypertension, diabetes mellitus, dyslipidemia, etc.), family history, and social risk factors such as habits of drinking and smoking. Therefore, the subjects included in this study tend to be older, have higher rates of males, and have higher rates of subjects with underlying risk factors for atherosclerosis than the general population. Even though we adjusted for conventional atherosclerotic risk factors, there may have been selection bias and caution should be taken when generalizing these results to the general population.

In conclusion, this study identified an association of chronic *Hp* infection with arterial stiffness in asymptomatic healthy subjects. In particular, the effect of *Hp* on arterial stiffness becomes more significant with persistent infection. In conclusion, this study has identified *Hp* as a novel and modifiable risk factor for CVD in adults. Although additional studies are required to validate these results, our findings indicate that routine screening for *Hp* may be indicated for all patients with CVD, especially in areas with a high prevalence of *Hp*.

## Supporting information

S1 Table Baseline characteristics of the 2,855 eligible subjects. (DOCX)

S2 Table STROBE statement—Checklist of items that should be included in reports of observational study (cross-sectional study). (PDF)

S1 Fig Distribution of CAVI values according to *H*. *pylori* seropositivity. (PDF)

S1 Appendix (Health Questionnaire_Original language version). (PDF)

S2 Appendix (Health Questionnaire_English version). (PDF)

S3 Appendix Dataset. (PDF)
