# Supplementary material for: Association between Helicobacter pylori infection and arterial stiffness: Results from a large cross-sectional study
Source: PLoS One. 2019 Aug 29;14(8):e0221643. doi: 10.1371/journal.pone.0221643 (PMC6715239; doi:10.1371/journal.pone.0221643)
Supplement: S1 Table — (DOCX) [file pone.0221643.s001.docx]

**S1 Table. Baseline characteristics of the 2,855 eligible subjects**

| Total subjects (n=2,855) | | |
| --- | --- | --- |
| Age, years |  | 55.0 (50.0-60.0) |
| Sex | Male | 2,072 (72.6) |
|  | Female | 783 (27.4) |
| BMI, kg/m2 |  | 24.2 (22.4-26.1) |
| Waist circumference, cm |  | 87.0 (82.0-92.5) |
| Smoking | Never | 1,652 (57.9) |
|  | Ever smoker^*^ | 1,203 (42.1) |
| Alcohol consumption | Not excessive | 2,151 (75.4) |
|  | Excessive | 703 (24.6) |
| Physical activity | Regular exercise | 1,013 (35.5) |
|  | Inactive | 1,842 (64.5) |
| Systolic BP, mmHg |  | 126.0 (116.0-136.0) |
| Diastolic BP, mmHg |  | 83.0 (77.0-90.0) |
| Pulse rate, bpm |  | 65.0 (59.0-72.0) |
| Hypertension |  | 1,353 (47.4) |
| Diabetes mellitus |  | 491 (17.2) |
| Dyslipidemia |  | 1,515 (53.1) |
| Fasting glucose, mg/dL |  | 100.0 (93.0-110.0) |
| Total cholesterol, mg/dL |  | 194.0 (170.0-217.0) |
| Triglyceride, mg/dL |  | 107.0 (74.0-156.0) |
| HDL-cholesterol, mg/dL |  | 52.0 (44.0-63.0) |
| LDL- cholesterol, mg/dL |  | 121.0 (100.0-142.0) |
| HbA1c, % |  | 5.6 (5.4-5.9) |
| BUN, mg/dL |  | 14.0 (12.0-17.0) |
| Creatinine, mg/dL |  | 0.9 (0.7-1.0) |
| CAVI, right |  | 7.7 (7.1-8.5) |
| CAVI, left |  | 7.6 (7.1-8.3) |
| CAVI, mean |  | 7.7 (7.1-8.4) |
| CAVI ≥ 8.0 |  | 1,066 (37.3) |

Values are presented as median (interquartile range) or n (%).

BMI, body mass index; BP, blood pressure; HDL-cholesterol, high-density lipoprotein cholesterol; LDL-cholesterol, low-density lipoprotein cholesterol; HbA1c, glycosylated hemoglobin; BUN, blood urea nitrogen; CAVI, cardio-ankle vascular index

^*^: Ever smoker was defined as a current or ex-smoker.
