# Supplementary figures and images for: Association between Helicobacter pylori infection and arterial stiffness: Results from a large cross-sectional study
Source: PLoS One. 2019 Aug 29;14(8):e0221643. doi: 10.1371/journal.pone.0221643 (PMC6715239; doi:10.1371/journal.pone.0221643)

Supplementary Figure 1. Distribution of CAVI values according to *H. pylori* seropositivity

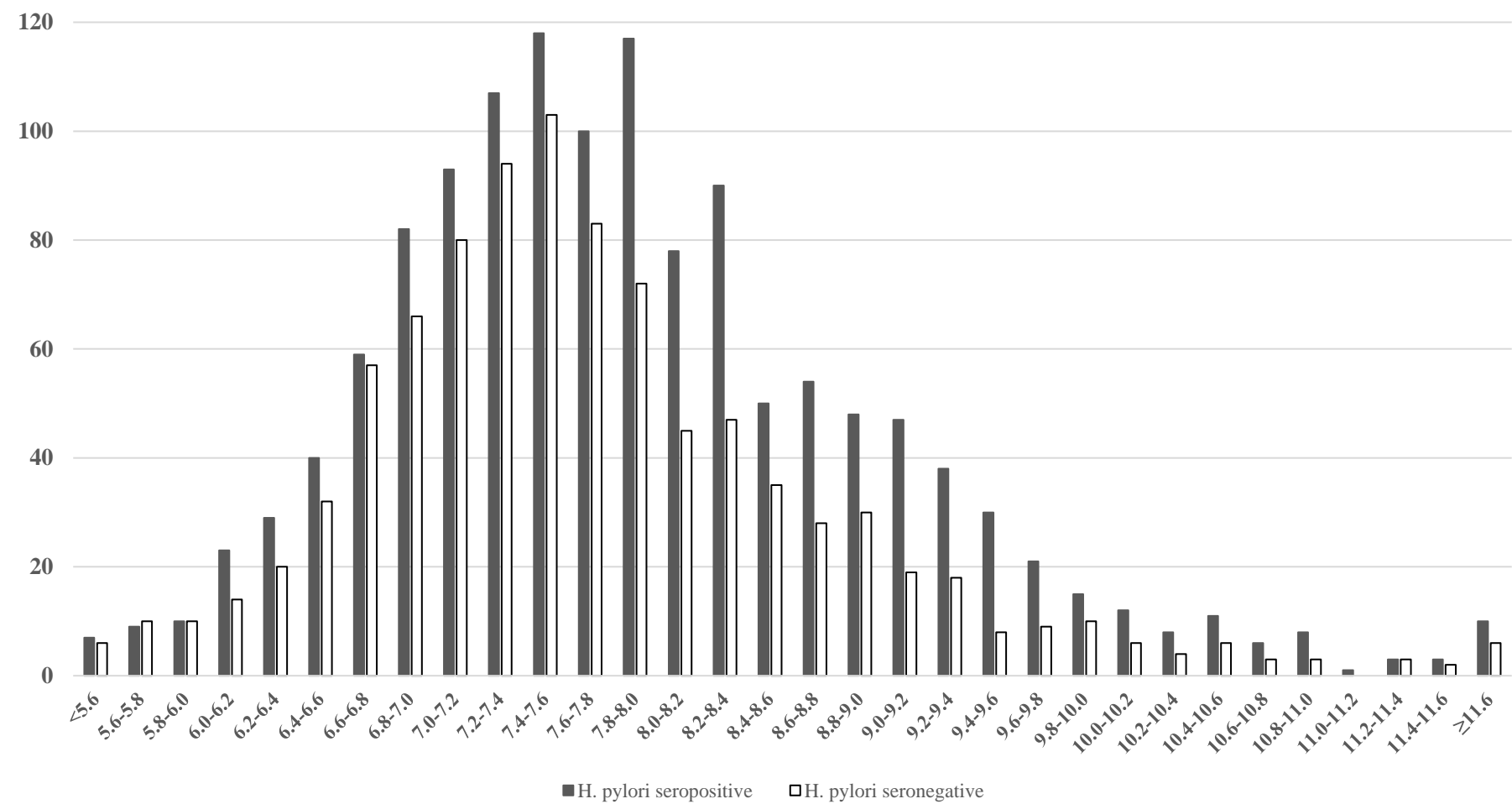

Supplement: S1 Fig — (PDF) [file pone.0221643.s003.pdf]
