# Supplementary material for: Association between Helicobacter pylori infection and arterial stiffness: Results from a large cross-sectional study
Source: PLoS One. 2019 Aug 29;14(8):e0221643. doi: 10.1371/journal.pone.0221643 (PMC6715239; doi:10.1371/journal.pone.0221643)
Supplement: S1 Appendix — (PDF) [file pone.0221643.s004.pdf]

국문

건강 문진표

방문일: □□-□□-□□□□

기본 정보			
성명	한글:	영문: (성)	(이름)
성별	남 □	여 □	
생년월일:	□□-□□-□□□□		

질병력

☐ 아래의 질환이 없다

질환명	질환있음	현재 투약중	비고
고혈압	☐	☐	
당뇨병	☐	☐	
고지혈증	☐	☐	
협심증/심근경색	☐	☐	☐ 관상동맥확장술/스텐트<br>☐ 수술
뇌졸중 (중풍)	☐	☐	
만성신장질환	☐	☐	☐ 신기능 저하<br>☐ 투석 중
간경변	☐	☐	
만성 B형 간염	☐	☐	
만성 C형 간염	☐	☐	
기타질환	☐		☐ 부위:<br>☐ 질환 명:

암	☐ 폐암 ☐ 위암 ☐ 대장암/직장암 ☐ 간암<br>☐ 유방암 ☐ 자궁경부암 ☐ 갑상선암 ☐ 전립선암<br>☐ 기타

복부 수술	수술부위:<br>☐ 위/십이지장 ☐ 대장/직장 ☐ 맹장<br>☐ 담낭 ☐ 간 ☐ 췌장<br>☐ 신장 ☐ 자궁 ☐ 난소/나팔관<br><br>진단명:

기타 수술력	수술부위:<br>진단명:

복용 약물

☐ 아래의 약을 복용하지 않는다.

약 이름	약 이름
☐ 아스피린<br>☐ 항응고제 (와파린/쿠마딘)<br>☐ 소염진통제<br>☐ 스테로이드제<br>☐ 갑상선호르몬<br>☐ 갑상선기능항진증약	☐ 골다공증약<br>☐ 칼슘제<br>☐ 진정제/수면제<br>☐ 한약<br>☐ 기타 ( )

헬리코박터 제균치료

헬리코박터 제균치료를 받은 적이 있습니까?

☐ 없다

☐ 있다 ▶ ☐ 제균치료 됨

☐ 제균치료 안 됨

☐ 모른다

가족력

부모, 형제, 자매, 자녀 중 아래의 질환을 앓았거나 그로 인해 사망한 경우가 있습니까?

질환 명	질환 명
☐ 고혈압<br>☐ 당뇨병	☐ 만성 간염/간경변<br>☐ 간암<br>☐ 위암

□ 협심증/심근경색 (남성 55세, 여성 65세 이전에 진단된 경우만 해당)<br>□ 뇌졸중 (중풍)	□ 대장암/직장암<br>□ 폐암

흡연력

지금까지 총 5갑 (100개비) 이상의 담배를 피운 적이 있습니까?

□ 아니오

□ 과거에 흡연을 하였으나 현재는 끊었음 ▶ 금연 하신 지는 몇 년 되셨습니까? 만 ( ) 년

금연 전까지 흡연 기간은? 총 ( ) 년

금연 전 하루 평균 흡연량은? ( ) 개비

□ 현재도 흡연 중 ▶ 흡연 기간은? 총 ( ) 년

하루 평균 흡연량은? ( ) 개비

음주력

술을 얼마나 자주 마십니까?

◦ 한 달에: □ 1번 이하 □ 2~4번

◦ 일주일에: □ 2번 □ 3번 □ 4번 □ 5번 □ 6번 □ 7번

술을 드실 때 한 번에 보통 몇 잔씩 마십니까?

(술 종류에 관계없이 각각의 술잔으로 계산합니다. 355cc 맥주 1캔 = 맥주 1.6잔)

□ 1~2잔 □ 3~4잔 □ 5~6잔 □ 7~9잔 □ 10잔 이상

운동력

지난 1주일 간, 평소보다 숨이 훨씬 더 차는 격렬한 활동을 하루 10분 이상 한 날은 며칠이었습니까?<br>(예: 달리기, 빠른 속도로 자전거 타기, 단식 테니스, 등산, 무거운 물건 나르기 등)	지난 1주일 간, 평소보다 숨이 조금 더 차는 중간 정도 활동을 하루 30분 이상 한 날은 며칠이었습니까?<br>(예: 보통속도 자전거 타기, 복식 테니스, 가벼운 물건 나르기, 걸레질하기 등)	지난 1주일 간, 한 번에 적어도 10분 이상 걸은 날은 며칠이었습니까?<br>(교통 수단 이용할 때 걸은 것뿐만 아니라 오락 활동, 여가 시간에 걸은 것도 포함)
☐ 0일 ☐ 1일<br>☐ 2일 ☐ 3일<br>☐ 4일 ☐ 5일<br>☐ 6일 ☐ 7일	☐ 0일 ☐ 1일<br>☐ 2일 ☐ 3일<br>☐ 4일 ☐ 5일<br>☐ 6일 ☐ 7일	☐ 0일 ☐ 1일<br>☐ 2일 ☐ 3일<br>☐ 4일 ☐ 5일<br>☐ 6일 ☐ 7일
그런 날 격렬한 활동을 하면서 보낸 시간:<br>평균 ☐☐☐ 분/일	그런 날 중간 정도 활동을 하면서 보낸 시간:<br>평균 ☐☐☐ 분/일	그런 날 걸으면서 보낸 시간:<br>평균 ☐☐☐ 분/일
