# Supplementary material for: Association between Helicobacter pylori infection and arterial stiffness: Results from a large cross-sectional study
Source: PLoS One. 2019 Aug 29;14(8):e0221643. doi: 10.1371/journal.pone.0221643 (PMC6715239; doi:10.1371/journal.pone.0221643)
Supplement: S2 Appendix — (PDF) [file pone.0221643.s005.pdf]

English

Health Questionnaire

Date of Visit: □□-□□-□□□□

Basic information

Name Last Name: First Name:

Gender Male □ Female □

Date of Birth (M-D-Y): □□-□□-□□□□

Past Medical History

☐ Not applicable to the below diagnosis

Diagnosis	Yes	Medication	Remark
Hypertension	☐	☐	
Diabetes	☐	☐	
Hyperlipidemia	☐	☐	
Angina/Myocardiac Infarction	☐	☐	☐ Percutaneous coronary intervention/Stent<br>☐ Operation
CVA (Stroke)	☐	☐	
Chronic Renal Disease	☐	☐	☐ Decreased renal function<br>☐ Dialysis
Liver Cirrhosis	☐	☐	
Chronic Hepatitis B	☐	☐	
Chronic Hepatitis C	☐	☐	
Others Diseases	☐		☐ Sites:<br>☐ Diagnosis:

Cancer	☐ Lung ☐ Stomach ☐ Colon/Sigmoid ☐ Liver<br>☐ Breast ☐ Cervix ☐ Thyroid ☐ Prostate<br>☐ Other

Abdominal Surgery	Lesion:<br>☐ Stomach/Duodenum ☐ Colon/Sigmoid ☐ Appendix<br>☐ Gall bladder ☐ Liver ☐ Pancreas<br>☐ Kidney ☐ Uterus ☐ Ovary/Fallopian tube<br><br>Diagnosis:

Other Surgery	Lesion:<br>Diagnosis:

Medicaion

□ Not applicable to the below medication

Medication	Medication
□ Aspirin<br>□ Anti-coagulant (Warfarin/Coumadin)<br>□ Anti-inflammatory agents<br>□ Steroids<br>□ Thyroid Hormone<br>□ Antithyroid Agent	□ Osteoporosis treatment<br>□ Calcium<br>□ Sedatives/Sleeping Pills<br>□ Oriental Herbal Medication<br>□ Others: ( )

Eradication of Helicobacter pylori

Have you ever had medications for eradication of Helicobacter pylori?

□ No

□ Yes ▶ □ Successfully eradicated

□ Failed to eradicate

□ Do not know

Family History

Have any of your family members (parents, siblings or children) been diagnosed or died with any of the following diseases?

Diagnosis	Diagnosis
□ Hylertension<br>□ Diabdetes<br>□ Angina/Myocardiac Infarction (If diagnosed under 55 years old in male or 65 years old in female)<br>□ Cerebrovascular accident (Stroke)	□ Chronic hepatitis/Liver cirrhosis<br>□ Liver Cancer<br>□ Stomach Cancer<br>□ Colon/Sigmoid Cancer<br>□ Lung Cancer

Smoking History

Have you smoked at least 5 packs (100 cigarettes) in the life?

☐ No

☐ Smoked in the past, but quit

▶ How long has it been since you quit smoking cigarettes? ( ) year(s)

How long have you smoked? ( ) year(s)

How many cigarettes did you smoke a day? ( ) cigarette(s)

☐ Currently smoking

▶ How long have you been smoking? ( ) year(s)

How many cigarettes do you smoke a day? ( ) cigarette(s)

Alcohol Drinking Habit

How often do you drink alcohol?

◦ Per month: ☐ less than once ☐ 2~4 times

◦ Per week: ☐ twice ☐ 3 times ☐ 4 times ☐ 5 times ☐ 6 times ☐ 7 times

How much do you usually drink at a time?

(Count each glass of the type of alcohol. Ex. Beer 1 can (355cc) = beer 1.6 glasses)

☐ 1~2 glass(es) ☐ 3~4 glasses ☐ 5~6 glasses

☐ 7~9 glasses ☐ more than 10 glasses

Exercise Related

Think about all the vigorous activities (activities that take hard physical effort and make you breathe much harder than normal) that you did in the last 7 days. Think only about those physical activities that you did for at least 10 minutes at a time. During the last 7 days, on how many days did you do vigorous physical activities like heavy lifting, digging, aerobics, or fast bicycling?	□ 0 □ 1<br>□ 2 □ 3<br>□ 4 □ 5<br>□ 6 □ 7	How much time did you usually spend doing vigorous physical activities on one of those days:<br>□□□ Minute(s)/day
Think about all the moderate activities (activities that take moderate physical effort and make you breathe somewhat harder than normal) that you did in the last 7 days. Think only about those physical activities that you did for at least 30 minutes at a time. During the last 7 days, on how many days did you do moderate physical activities like carrying light loads, bicycling at a regular pace, or doubles tennis?	□ 0 □ 1<br>□ 2 □ 3<br>□ 4 □ 5<br>□ 6 □ 7	How much time did you usually spend doing moderate physical activities on one of those days:<br>□□□ Minute(s)/day
Think about the time you spent walking in the last 7 days. This includes at work and at home, walking to travel from place to place, and any other walking that you might do solely for recreation, sport, exercise, or leisure. During the last 7 days, on how many days did you walk for at least 10 minutes at a time?	□ 0 □ 1<br>□ 2 □ 3<br>□ 4 □ 5<br>□ 6 □ 7	How much time did you usually spend walking on one of those days:<br>□□□ Minute(s)/day
