# Supplementary material for: Association between Helicobacter pylori infection and arterial stiffness: Results from a large cross-sectional study
Source: PLoS One. 2019 Aug 29;14(8):e0221643. doi: 10.1371/journal.pone.0221643 (PMC6715239; doi:10.1371/journal.pone.0221643)
Supplement: S3 Appendix — (PDF) [file pone.0221643.s006.pdf]

Sex	Age	BMI	Waist	Smoking	Alcohol	Exercise	HTN	DM	Dyslipid	SBP	DBP	PR	CAVI_Rt	CAVI_Lt	CAVI_mean	GLU	BUN	CR	CHOL	TG	HDL	LDLC	HBA1C	H.pylori
1	68	24.4	89	1	1	1	1	0	1	141	88	69	8.9	8.5	8.7	117	10	0.89	167	145	49	98	5.6	1
2	81	20.8	79	1	2	1	1	0	1	148	81	52	9.3	9.3	9.3	81	18	0.55	245	109	78	142	5.5	1
1	65	27.9	94.5	1	1	1	1	0	0	149	95	72	9.2	8.7	8.95	89	15	0.94	171	108	42	107	5.3	1
2	63	20.8	75	1	1	1	1	1	0	128	82	79	9.3	9.1	9.2	146	16	0.63	215	78	57	142	7.9	1
2	54	20.8	83	1	1	1	0	0	1	129	83	60	7.5	7.6	7.55	86	14	0.74	265	128	66	166	6.1	0
1	60	25.9	88	1	2	1	1	0	1	119	90	75	7.7	7.7	7.7	99	15	0.91	166	81	48	105	5.4	0
1	53	26.4	94	2	1	1	1	1	1	146	103	83	9	8.3	8.65	176	20	0.97	134	152	59	69	6.5	0
2	59	24.1	82	1	1	1	0	1	0	128	70	61	7.9	8	7.95	107	15	0.59	232	108	48	159	6.9	0
2	43	24.7	79	2	1	1	1	0	0	120	80	75	6.9	7.2	7.05	85	14	0.65	191	70	58	117	5.4	1
2	43	22.6	84	2	1	1	1	0	0	114	74	65	6.9	6.9	6.9	80	10	0.69	159	64	44	96	5.6	1
1	56	28.7	100	2	1	1	0	0	1	127	84	65	6.6	6.6	6.6	112	12	0.82	229	162	47	143	5.9	1
1	65	25.4	91	1	1	1	0	1	0	128	85	63	10	10.1	10.05	93	27	1.25	137	63	56	69	6.1	0
2	59	27.7	87	1	1	1	0	0	1	138	89	62	7.7	7.5	7.6	106	21	0.84	257	113	58	170	5.7	0
2	51	27.1	89	1	1	1	0	0	0	115	77	66	7.5	7.6	7.55	84	13	0.83	188	53	50	127	5.9	1
2	44	27.7	90	1	1	1	1	0	0	121	77	59	7.4	7.1	7.25	88	12	0.79	144	69	45	85	5.4	0
2	51	22.5	83	2	1	1	0	0	0	129	76	73	6.5	6.3	6.4	80	11	0.7	177	67	73	89	5.4	1
1	72	24.9	85	1	1	1	1	1	1	138	97	73	10.9	10.2	10.55	79	17	0.83	154	67	35	113	6.4	1
1	67	22.1	82	1	1	1	1	0	0	160	105	89	10	9.7	9.85	113	24	0.88	239	54	88	135	5.4	0
1	55	23.4	85	2	1	1	1	0	0	138	96	59	10.5	7.5	9	108	12	0.76	197	71	64	119	5.4	1
2	62	21.3	84	1	1	2	0	0	1	109	69	80	7.5	7.5	7.5	95	12	0.69	171	89	53	101	5.6	1
2	56	20.8	71	1	1	1	0	0	1	104	69	66	8.4	8.1	8.25	81	22	0.72	275	58	77	175	5.6	1
1	49	20.0	74	2	1	1	0	0	1	111	79	73	7.7	7.9	7.8	78	19	0.82	244	83	69	154	5.4	0
1	68	26.9	91	2	1	2	1	0	1	134	84	55	8.2	8	8.1	96	13	1.19	140	164	32	83	5.5	1
1	21	28.7	94	1	1	2	0	0	0	137	65	61	5.8	5.6	5.7	89	12	0.9	153	104	45	89	4.8	0
2	33	24.1	77	1	1	1	0	0	0	107	63	59	6.1	6.1	6.1	83	13	0.79	181	79	52	111	5.3	1

1	68	24.3	83	2	1	1	0	0	0	125	81	54	9.2	8.9	9.05	94	19	0.8	149	36	54	85	5.4	1
2	71	21.5	83	1	1	1	1	0	1	127	88	71	10	9.8	9.9	94	7	0.55	166	119	35	106	5.4	1
2	57	21.9	86	1	1	1	0	0	0	101	70	66	7.2	7.5	7.35	91	13	0.59	235	91	58	153	4.8	0
1	61	19.3	74.5	2	1	1	0	0	0	125	75	63	7.3	7.4	7.35	113	12	0.95	156	93	51	80	5.7	1
1	56	26.9	100	2	1	1	1	1	1	139	90	59	10.2	9	9.6	94	19	0.76	140	95	46	81	6.8	1
2	58	23.0	86	1	1	1	1	0	1	132	72	74	7.4	7.5	7.45	86	23	0.7	252	60	66	163	5.3	0
2	66	23.8	81	1	1	1	1	0	1	117	80	47	8.1	8	8.05	96	20	0.89	170	150	41	106	6.1	1
1	68	28.3	96	1	1	1	1	1	1	121	75	44	8.8	9	8.9	155	20	1.05	223	204	47	148	7.6	0
2	64	20.3	72	2	1	1	0	0	1	121	62	43	9.6	9.3	9.45	91	18	0.7	302	131	57	201	5.6	1
1	34	26.2	85	1	2	2	0	0	1	134	86	59	6.7	6.7	6.7	109	17	0.9	358	209	48	282	5.6	0
2	35	18.8	68	1	1	2	0	0	0	118	71	53	6.4	6.5	6.45	84	16	0.85	183	66	57	108	5.4	1
1	63	22.1	88	2	1	1	1	0	1	125	77	62	9.5	9.4	9.45	114	17	1.05	269	312	39	163	5.9	1
1	46	29.1	99	1	2	1	1	1	1	154	106	72	8.3	8.7	8.5	208	10	0.84	158	209	48	58	11.4	1
1	64	24.7	93	1	1	2	1	1	1	131	93	76	10.8	9.2	10	140	18	0.92	175	53	61	107	7.4	1
2	69	24.0	85	1	1	1	1	0	1	118	74	47	8.6	8.4	8.5	93	17	0.75	173	178	57	88	5.4	0
1	66	29.4	103	1	2	1	1	0	1	137	92	59	8.6	9.4	9	107	20	0.99	180	135	58	99	5.6	1
1	60	21.5	82	1	2	1	0	0	0	122	84	56	7.8	7.6	7.7	98	15	0.86	190	60	71	98	5.4	0
2	54	24.3	84	1	1	2	0	0	1	110	67	54	6.3	6.4	6.35	86	14	0.72	385	68	79	264	5.4	0
2	53	21.0	78	1	1	2	0	0	0	113	75	53	7.3	7.4	7.35	92	18	0.76	233	96	52	149	5.5	1
1	44	27.4	95	1	2	1	1	0	1	146	99	53	9	7.7	8.35	101	13	0.93	229	132	60	154	5.3	0
1	71	26.6	89	2	2	1	1	1	1	155	90	80	10.9	12.2	11.55	132	19	0.91	195	65	43	148	6.3	1
1	70	20.0	76	1	1	2	0	0	1	108	71	65	8	7.8	7.9	90	20	1.06	244	57	61	165	5.7	1
1	53	30.5	100	2	1	2	0	0	1	136	89	69	6.8	6.6	6.7	109	15	1.03	151	116	52	80	5.8	1
2	68	20.6	85	1	1	1	1	0	0	131	85	77	10.3	10.1	10.2	83	23	0.67	205	80	65	119	5.2	0
2	70	21.2	80	1	1	1	0	1	1	127	81	63	8.8	9.3	9.05	102	16	0.7	185	242	50	86	6.6	1
1	67	22.0	87	1	1	2	0	0	1	128	83	59	8.2	7.8	8	91	24	0.72	259	92	63	177	5.5	0
1	51	24.5	86	1	1	1	0	0	0	119	88	58	8	7.6	7.8	106	17	0.82	194	181	48	109	5.6	1
1	60	25.7	91	2	1	2	1	1	0	140	95	70	8.1	8	8.05	128	15	1.03	212	177	59	131	6.7	1
2	57	19.5	79.5	1	1	2	0	0	0	120	70	62	7.4	7.6	7.5	89	15	0.55	201	78	57	130	5.4	0

2	56	20.4	79	1	1	1	0	0	1	117	74	69	8.5	8.2	8.35	94	16	0.72	201	212	50	111	5.9	1
2	55	24.7	88	1	1	2	1	0	0	148	90	57	8.7	8.3	8.5	91	17	0.47	186	54	57	115	5.9	0
2	65	27.2	102	1	2	2	1	1	1	137	94	50	8	8	8	143	15	0.58	246	74	75	152	5.3	0
1	65	29.0	99	2	1	2	1	0	1	145	85	58	8.8	9	8.9	114	16	0.8	208	204	38	138	5.7	1
1	65	23.0	92	2	2	2	1	0	1	125	85	64	8	8.8	8.4	90	14	0.9	232	78	48	174	5.6	1
1	72	20.8	81.5	2	1	2	1	1	1	134	79	68	12	10.6	11.3	206	12	0.85	153	160	33	103	7.5	1
2	67	25.6	93	1	1	2	1	1	0	152	87	57	8.7	8.9	8.8	118	17	0.74	172	106	45	114	6.7	1
1	68	24.0	84	2	1	2	0	0	1	116	74	64	9	9.1	9.05	95	14	0.85	240	56	56	173	5.4	1
2	57	19.8	76	1	1	1	0	0	1	110	71	63	8.3	8.4	8.35	94	15	0.76	166	54	68	90	5.9	0
2	42	18.3	71	1	1	1	0	0	0	115	72	66	8.2	7.6	7.9	91	11	0.59	196	51	64	116	5.4	1
1	56	25.1	81	2	1	1	0	1	1	126	86	67	9.9	12.5	11.2	169	15	1.04	231	155	67	130	7.5	0
1	51	22.3	87	2	1	2	1	0	0	121	79	72	6.9	7	6.95	120	16	0.76	197	88	45	141	5.6	1
1	41	27.8	91	2	1	1	1	0	1	128	83	84	7.5	7.6	7.55	115	11	0.88	168	200	48	96	5.8	1
2	66	19.8	82	1	1	1	0	1	0	122	79	64	8	7.8	7.9	124	15	0.73	225	66	72	130	7.4	0
1	44	32.2	99	2	1	1	1	0	1	147	84	47	6	5.9	5.95	85	16	0.91	226	177	40	164	5.4	0
2	54	27.5	98	1	1	1	0	0	0	115	70	58	6.3	6.4	6.35	92	13	0.96	214	87	52	138	5.5	1
2	58	26.3	90	1	1	1	1	0	1	143	95	83	7.6	7.9	7.75	100	11	0.53	175	66	57	101	5.5	0
1	45	25.8	90	2	1	1	0	0	0	128	84	56	7	7	7	89	12	1.16	214	127	43	152	5.4	0
1	43	26.3	87	2	2	1	0	0	0	120	86	71	7.5	7.4	7.45	95	11	1.06	214	161	69	129	5.3	1
1	43	27.4	95	2	1	1	1	0	1	129	85	54	7.3	7.2	7.25	98	16	0.98	179	130	45	119	6.1	1
1	61	24.9	90	2	2	1	1	0	1	145	89	55	8.5	8.4	8.45	107	16	0.78	231	102	47	162	5.6	1
1	60	24.1	86	2	1	2	1	0	1	135	85	76	8.3	8	8.15	74	15	1.15	181	61	65	101	5.5	1
2	36	17.3	63.5	1	1	1	0	0	0	91	58	64	7.7	7.7	7.7	84	8	0.71	212	116	61	116	5.2	0
1	41	26.5	93	1	2	2	0	0	0	119	76	63	6.9	6.8	6.85	90	14	0.93	212	158	46	136	5.3	0
1	36	24.0	90	2	2	2	0	0	0	132	86	47	7.2	7.3	7.25	92	13	1.18	206	194	57	121	5	1
1	50	24.4	87	2	2	1	0	0	1	121	85	69	7.7	8	7.85	105	14	0.8	271	313	46	180	5.4	0
2	36	23.6	84	1	1	2	0	0	0	122	73	56	6	6.2	6.1	92	9	0.55	173	59	53	99	5.8	1
1	66	25.1	92	2	1	1	0	0	0	108	71	57	8.8	8.4	8.6	112	11	1.06	167	55	41	116	5.7	0
2	67	24.3	85	1	1	2	0	1	1	122	77	66	8.2	7.6	7.9	102	15	0.69	207	94	54	135	6.5	1

2	53	19.0	75	1	1	2	0	0	0	126	77	52	7.8	8	7.9	88	14	0.58	208	66	78	112	5.2	0
1	44	33.2	109	1	1	1	0	0	1	132	77	55	7	6.9	6.95	97	14	1.08	195	198	37	133	5.3	0
1	47	25.3	94	2	1	2	0	0	0	125	83	56	8.2	8	8.1	86	15	1.24	192	89	57	118	5.4	1
1	41	29.4	93	1	2	1	0	0	1	118	89	61	6.8	6.6	6.7	100	18	1.06	294	113	65	208	5.3	1
1	77	27.3	96	2	2	2	1	1	1	126	80	74	9.3	8.9	9.1	113	14	0.89	203	66	46	145	6.5	1
1	46	25.5	96	2	1	1	0	0	0	134	85	64	7.2	7.5	7.35	103	16	0.85	207	121	57	134	6.2	0
1	39	26.0	92	1	1	2	0	0	0	108	67	51	7.6	7.5	7.55	92	12	1.17	150	77	41	95	5.2	0
2	31	18.9	69	2	1	1	0	0	0	135	89	74	8	7.4	7.7	74	18	0.65	232	67	75	139	5	1
1	70	23.3	95	1	2	2	1	0	1	131	97	63	9.2	8.5	8.85	93	20	0.78	228	294	52	139	5.4	0
1	56	22.4	80.5	2	1	1	1	0	1	134	93	60	8.2	8.1	8.15	102	11	0.85	284	88	41	230	5.7	1
1	61	26.2	88	1	2	1	1	0	0	127	88	61	7.7	8.1	7.9	112	19	1.03	177	73	56	112	5.5	0
1	47	29.3	107	2	1	1	0	0	1	139	88	77	7.9	8.1	8	99	13	1.02	222	96	45	164	5.3	0
1	74	20.1	77	2	2	1	1	0	0	126	79	55	10.6	9.8	10.2	112	17	1.09	238	179	57	157	6.4	1
1	58	26.5	94	2	1	2	1	0	1	128	95	53	7.7	7.2	7.45	99	12	1.04	170	124	37	105	5.8	1
2	47	25.6	87	1	1	1	0	0	0	128	81	65	6	5.9	5.95	97	11	0.8	169	150	46	102	5.3	0
1	65	29.1	108	1	2	1	1	1	1	153	101	66	10.7	11.1	10.9	135	17	0.92	207	91	60	131	5.3	1
2	63	20.8	77	1	1	2	0	0	0	110	79	53	9.4	9.3	9.35	86	16	0.59	197	68	65	103	5.4	0
1	50	22.5	87	1	1	1	0	0	0	104	72	50	7.6	7.6	7.6	82	15	0.94	201	59	69	116	5.4	0
1	59	27.3	93	2	2	1	1	0	1	110	77	71	7.6	7.8	7.7	119	17	0.95	203	151	42	141	5.8	1
2	55	23.9	81	1	1	1	0	0	1	117	79	64	6.9	7.2	7.05	90	15	0.64	251	173	69	143	5.6	0
1	49	21.7	87	2	2	1	0	0	0	123	81	56	8.5	8.5	8.5	98	9	0.87	221	177	55	139	5.7	0
2	59	20.5	75.5	1	1	1	0	0	0	124	88	60	8.4	8.7	8.55	83	15	0.92	219	48	69	134	5.6	0
1	55	28.3	97.5	2	2	2	1	0	0	132	97	66	6.6	6.3	6.45	77	12	0.67	168	44	66	93	5.7	1
2	79	25.2	94	1	1	1	1	1	0	137	82	91	10.3	9.7	10	174	21	0.65	234	128	54	158	7.8	1
2	63	26.6	92	1	1	2	1	0	1	140	87	62	9.3	7.7	8.5	97	11	0.65	293	160	56	213	6	1
2	51	29.2	101	1	1	1	0	1	1	119	81	75	7.3	7.1	7.2	171	17	0.56	266	294	48	160	10.6	1
2	74	25.2	88.5	1	1	2	1	0	0	143	83	80	8.9	9.1	9	81	18	0.74	174	67	47	111	5.7	1
2	57	24.0	83	1	1	1	1	0	0	136	92	64	10.1	9.7	9.9	109	19	0.63	237	81	70	145	5.8	1
1	39	25.6	92	2	2	2	0	0	1	118	81	62	8.1	7.7	7.9	108	13	0.86	222	189	43	153	5.5	1

2	57	26.8	86.5	2	1	1	1	0	0	154	91	60	7.5	7.4	7.45	89	18	0.77	200	197	58	106	5.4	1
1	63	24.7	93.5	2	1	1	1	0	1	117	85	66	8.4	7.5	7.95	86	15	0.96	240	83	56	169	5.5	1
1	55	26.3	95	2	1	1	1	1	0	144	98	61	9.8	9	9.4	153	12	0.82	167	181	45	102	8.1	1
2	62	19.9	84	1	1	1	0	0	1	111	77	73	7.1	7.2	7.15	101	10	0.75	235	80	49	163	5.4	1
1	55	23.0	84	1	1	2	1	0	0	139	92	55	6.3	6.2	6.25	90	11	0.88	211	80	53	135	5.6	1
1	55	23.8	89	1	1	2	1	0	0	133	91	65	7.5	7.1	7.3	100	15	1.07	208	132	46	140	5.2	0
1	48	24.7	85	2	1	1	1	0	0	134	94	61	8.1	7.8	7.95	97	21	0.97	190	128	49	120	5.1	1
2	71	27.6	91.5	1	1	2	1	0	1	150	101	61	10.4	9.5	9.95	88	19	0.65	187	192	36	117	5.9	0
1	48	25.1	92	2	2	1	1	1	1	137	90	66	8.3	8.1	8.2	145	16	0.69	208	227	49	112	7.8	1
1	60	23.2	86	1	1	2	1	0	0	139	91	81	7.7	7.7	7.7	99	18	0.97	189	82	65	105	6.1	0
2	79	23.1	84.5	1	1	1	1	0	1	160	93	67	10.5	10.2	10.35	101	16	0.62	208	157	67	108	5.8	1
1	62	25.2	84	2	2	1	1	0	1	150	104	62	8	7.4	7.7	104	16	0.77	238	124	67	148	5.4	1
1	58	25.9	93.5	2	2	1	1	1	0	129	84	71	9	10.6	9.8	137	12	0.69	188	108	43	126	7.3	0
2	60	26.5	93	1	1	1	0	1	1	115	73	62	9.3	9.2	9.25	123	19	0.74	211	134	65	121	7.1	1
2	31	16.2	59	1	1	1	0	0	0	108	70	62	7.1	7.1	7.1	76	13	0.72	166	41	63	92	5.3	0
2	61	22.1	77	1	1	1	0	0	0	126	77	69	7.1	6.7	6.9	100	13	0.67	217	89	65	136	5.7	0
1	40	25.0	82	2	1	1	0	0	1	120	69	79	7	6.9	6.95	74	12	1.21	185	250	28	60	5.4	1
2	59	28.6	95.5	2	1	1	1	0	1	124	91	68	7.9	7.8	7.85	96	11	0.76	248	143	47	164	5.5	1
1	50	23.9	90	1	2	1	1	0	0	123	84	73	6.7	6.5	6.6	107	13	0.83	136	89	43	83	5.9	0
1	55	25.1	93	2	1	2	1	0	1	124	88	80	9	8.8	8.9	107	33	1.15	137	74	36	93	5.8	0
2	53	24.6	79	1	1	2	0	0	1	108	80	75	6.9	6.8	6.85	73	23	0.72	256	49	57	190	5.5	0
1	53	27.6	97	2	2	1	0	1	1	123	87	61	7.1	7.4	7.25	110	11	0.99	146	274	37	78	7	1
1	45	22.4	84	1	1	1	0	0	1	116	83	65	8	7.8	7.9	94	11	1.01	215	262	48	131	5.4	1
2	49	20.6	71.5	1	1	1	0	0	0	112	74	67	8	8.1	8.05	83	9	0.68	185	44	57	111	5.3	1
2	58	29.1	89	1	1	1	1	0	0	140	96	59	7.6	7.2	7.4	101	10	0.63	206	121	58	130	6.1	1
1	72	23.2	89	1	1	1	1	0	1	148	88	72	8.9	9.4	9.15	78	16	1.43	245	96	55	191	5.5	0
1	40	25.4	92	1	2	2	0	0	1	124	73	73	6.4	6.2	6.3	77	17	1.08	219	72	73	137	5.7	1
2	49	27.2	88	1	1	1	0	0	0	109	76	75	7.4	7.2	7.3	85	14	0.54	199	33	56	139	5.4	0
1	55	23.8	90.5	2	2	1	1	0	0	129	81	72	8.6	8.1	8.35	90	12	0.64	160	66	75	84	5.3	0

2	54	19.4	75	1	1	2	0	0	0	102	66	55	8	7.7	7.85	92	13	0.66	186	77	44	125	5.7	0
1	48	27.3	94	1	2	1	1	0	1	133	99	62	6.5	10	8.25	88	12	0.85	153	119	36	106	5.5	0
1	75	22.4	91	1	1	1	0	0	0	131	83	53	10.1	10.7	10.4	91	20	0.96	145	65	43	91	6.1	1
2	55	26.4	94	1	1	1	1	1	1	118	87	67	7.2	7.2	7.2	133	23	0.81	151	76	59	75	6.5	1
1	53	26.2	96	2	2	1	1	0	1	145	99	62	8.7	8.6	8.65	87	15	0.71	186	319	40	99	5.3	0
1	49	24.6	87	2	1	1	0	0	1	121	82	71	6.8	6.3	6.55	96	15	0.9	243	147	58	148	5.5	0
1	43	26.0	96	2	2	1	0	1	1	124	85	92	8	6.7	7.35	133	7	0.77	207	208	42	145	5.9	1
1	59	21.0	83	2	2	1	0	0	1	121	87	64	8	8	8	96	12	0.82	282	143	70	181	5.4	1
1	44	26.2	98	2	1	1	1	0	0	140	97	79	6.3	6.2	6.25	117	12	0.87	222	177	49	152	6	0
2	68	33.8	104	1	1	1	1	1	1	128	83	66	7.3	7.6	7.45	104	20	0.69	145	128	44	82	6.5	1
2	66	26.3	98	1	1	2	1	1	1	139	75	60	9.4	9.2	9.3	118	15	0.83	157	110	65	70	6.8	1
1	60	26.9	103	1	1	1	1	0	1	129	87	56	7.4	7.2	7.3	104	13	0.99	293	160	49	216	5.6	0
2	56	21.3	83	1	1	1	0	0	1	107	68	62	7.7	8.1	7.9	85	15	0.8	242	71	60	163	5.5	1
1	61	24.2	85	1	2	1	0	0	0	124	86	54	7.4	7.1	7.25	101	11	1.05	205	105	54	126	5.9	0
2	54	23.1	86	1	1	1	0	0	0	107	71	45	8.2	8.5	8.35	107	10	0.63	196	190	47	108	5.7	0
1	60	21.8	83.5	1	1	1	1	0	0	145	95	48	7.7	7.6	7.65	95	17	0.65	144	77	55	81	5.4	1
1	48	24.3	92.5	2	2	1	0	0	1	112	75	72	7.2	7.3	7.25	101	12	0.78	183	225	53	91	5.4	0
2	56	20.6	73	1	1	1	0	0	0	109	71	55	7.7	7.7	7.7	84	16	0.73	186	96	58	105	6	0
1	63	18.7	70	2	1	1	0	1	1	117	76	71	9.2	9.1	9.15	95	15	0.67	150	92	37	96	6.5	0
1	60	22.8	85.3	2	2	1	1	0	0	138	93	53	8.7	9.2	8.95	109	24	0.88	193	158	41	130	5.8	0
2	57	23.4	85	1	1	2	0	1	1	129	82	66	8.7	8.2	8.45	131	12	0.68	177	231	44	95	8.3	1
2	58	24.0	85	1	1	1	1	0	0	147	87	87	7.4	7.7	7.55	124	13	0.76	208	49	56	143	6.1	0
1	60	23.3	81	1	1	1	0	1	1	112	73	61	7.7	7.7	7.7	125	15	0.92	117	113	37	71	7.4	0
1	52	28.4	102	2	1	1	1	0	1	148	107	80	7.5	7.6	7.55	109	9	0.95	210	479	51	109	6.3	0
1	57	25.7	88	2	1	1	1	0	1	128	90	60	7.8	7.8	7.8	109	12	0.93	283	53	50	233	5.5	1
2	57	22.2	82.5	1	1	1	1	0	1	155	93	88	6.8	6.5	6.65	108	12	0.73	315	131	62	225	5.7	1
2	50	26.7	96	1	1	1	0	0	0	110	85	57	6.4	6.3	6.35	94	14	0.63	162	81	62	92	5.7	0
1	59	23.8	85	1	2	1	1	0	0	116	84	81	8.3	8.3	8.3	95	15	1.1	127	51	42	75	5.6	1
2	74	24.9	93	1	1	1	1	1	1	148	84	58	10.7	10.9	10.8	101	25	0.73	170	102	46	101	7	0

1	46	31.5	104	1	2	1	1	1	1	147	97	90	7.3	6.8	7.05	128	14	0.82	161	246	48	79	6.9	0
1	39	29.7	112	1	2	1	0	0	0	128	87	46	7.5	7	7.25	106	15	0.76	193	155	40	132	5.8	1
2	37	18.4	72.5	1	1	2	0	0	0	94	66	61	5.4	5.4	5.4	85	14	0.75	160	45	68	81	5.4	0
2	47	19.1	73	1	1	1	1	0	0	140	83	68	7.8	7.7	7.75	84	13	0.73	191	47	75	98	5.3	1
2	43	18.1	66	1	1	1	0	0	0	135	82	64	7.5	7.6	7.55	81	14	0.84	226	54	89	119	5.3	1
1	89	21.9	84	2	1	1	1	1	1	151	78	68	12.2	10.4	11.3	131	19	0.89	170	83	42	116	7.6	1
2	38	21.8	83	1	1	1	0	0	0	122	86	56	6.5	6.3	6.4	84	11	0.71	176	51	63	99	5.6	0
2	45	19.6	74	1	1	1	1	0	0	140	100	72	7	7.1	7.05	91	9	0.67	220	54	79	124	5.4	1
2	64	17.1	71	1	1	1	1	0	0	137	91	62	7.2	6.9	7.05	92	18	0.78	238	43	74	145	5.5	1
2	57	24.8	76.5	1	1	1	0	0	1	129	79	66	6.9	7	6.95	87	9	0.69	143	70	39	96	5.3	0
1	45	28.0	96.8	1	2	1	1	0	0	140	94	61	8	7.6	7.8	106	13	1.01	210	162	56	133	5.4	1
2	67	24.2	88	1	1	1	1	0	0	113	78	61	8.5	8.2	8.35	98	15	0.72	239	113	75	131	5.6	0
1	51	26.1	89	1	2	1	1	0	0	112	74	73	6.7	6.2	6.45	104	22	0.91	172	74	48	117	5.4	1
1	49	21.9	83	1	1	1	1	1	1	134	95	75	7.5	7.4	7.45	156	11	0.75	253	239	48	164	7.1	0
1	49	23.7	84	2	2	1	0	0	0	118	85	53	8.7	8.3	8.5	106	21	1.03	218	194	52	146	5.5	0
1	39	22.8	86	1	1	1	0	0	0	122	84	66	7.1	6.9	7	99	12	1.1	186	122	44	127	5.5	0
1	40	22.0	77	2	1	1	0	0	0	109	59	48	7.6	7.4	7.5	74	13	1.11	158	45	49	99	5.2	0
2	53	26.3	97	1	2	1	1	0	0	135	91	64	8.5	8.5	8.5	101	16	0.78	167	157	51	95	5.7	0
1	51	25.1	90	1	1	1	1	0	0	111	75	62	7.6	7.6	7.6	100	15	1.14	212	104	47	148	5.3	1
1	60	20.5	80	1	1	1	0	0	1	118	81	71	7.1	7.1	7.1	87	15	1.12	203	59	49	144	5.5	1
2	56	22.1	78.5	1	1	1	0	0	1	131	81	56	7.8	7.8	7.8	73	12	0.75	249	131	59	156	5.6	1
1	59	27.8	100	1	1	1	1	0	1	135	86	55	7.7	7.4	7.55	106	20	0.83	177	118	39	122	6.1	0
2	61	29.3	101	1	1	1	0	0	1	130	87	66	7.5	7.4	7.45	110	14	0.86	266	147	49	193	6.4	1
2	60	24.1	83	1	1	1	1	0	0	149	94	63	7.8	7	7.4	92	14	0.52	195	129	44	128	5.8	0
1	63	27.4	98	2	2	1	1	0	1	121	84	75	8.6	8.6	8.6	83	10	0.69	139	116	47	81	5.3	1
1	48	25.0	88	1	1	1	0	0	0	127	85	66	7.7	7.7	7.7	94	11	0.95	193	115	48	135	5.3	1
1	63	21.0	82	2	2	1	1	0	0	135	96	48	8.1	8	8.05	104	13	0.84	190	102	43	128	5.4	1
1	49	26.0	86.5	1	1	1	0	0	1	125	81	60	7.1	6.7	6.9	103	15	1.19	205	96	39	154	5.9	1
2	70	26.1	95	1	1	1	0	0	1	112	74	55	7.3	7.3	7.3	97	13	0.69	202	62	63	124	5.8	1

2	39	25.0	87	1	1	1	0	0	0	115	79	72	7.1	7.1	7.1	90	12	0.72	200	100	41	146	6	0
1	51	25.5	91	2	1	1	0	0	0	126	74	57	8	7.9	7.95	93	14	0.78	208	66	61	135	5.4	1
1	43	29.1	96.5	2	1	1	1	0	1	144	93	92	8	7.9	7.95	117	12	1.01	206	429	37	118	5.6	1
1	50	19.4	77.6	2	1	2	0	0	0	117	77	81	8.3	8.6	8.45	91	20	0.89	173	36	51	112	5.8	0
1	53	24.4	87	1	1	1	1	1	1	129	76	61	7.2	7.6	7.4	168	19	0.91	161	176	69	80	6.2	0
2	48	22.3	79	1	1	2	0	0	0	102	65	45	7.4	7.4	7.4	67	17	0.7	207	30	69	129	5.4	1
1	53	30.1	103	1	1	1	1	1	1	154	106	59	9.5	8.5	9	164	24	1.28	185	284	51	97	8.2	1
1	49	24.7	88	2	1	2	0	0	0	126	79	78	7.9	7.8	7.85	96	11	1.01	179	145	40	113	5.3	1
1	51	24.1	80	1	2	2	0	0	0	101	64	50	7.2	7.3	7.25	92	10	0.91	142	54	56	75	5.3	1
2	50	28.4	93.5	1	1	2	1	0	1	136	90	49	7.8	7.6	7.7	123	15	0.79	254	159	41	184	6.4	0
1	62	22.9	90	2	1	1	0	0	0	124	79	62	8.3	8.6	8.45	95	28	0.92	192	51	66	119	5.4	1
1	63	16.7	74.5	2	2	1	1	0	0	127	92	75	8.3	8.2	8.25	88	11	0.89	171	79	46	103	5.4	1
1	42	22.2	83	2	1	1	1	0	1	146	105	84	8.3	7.7	8	98	14	0.97	226	131	42	165	5.7	0
1	62	26.0	90.5	1	1	1	1	0	1	108	76	52	7.7	7.7	7.7	109	15	0.85	216	118	36	154	5.5	1
1	52	21.6	74.8	2	2	1	0	0	1	123	78	54	8.6	8.6	8.6	98	13	0.76	176	52	56	102	5.5	1
2	47	25.2	87	1	1	2	0	0	1	103	69	81	6.9	6.8	6.85	90	15	0.86	184	85	50	110	5.8	0
1	58	23.4	88	2	1	1	0	0	1	117	79	58	8.4	8.2	8.3	97	14	0.93	218	203	46	145	5.4	0
2	65	20.7	73	1	1	1	0	0	0	131	75	68	8.9	8.9	8.9	85	21	0.62	221	139	53	134	6.2	0
2	62	21.8	77.2	1	1	1	1	0	0	137	81	66	9	8.6	8.8	94	13	0.59	230	53	67	144	5.9	1
1	45	26.2	91	2	1	1	0	0	1	124	81	64	8.1	7.8	7.95	94	14	0.97	254	305	45	158	5.4	1
2	58	18.4	62	1	1	1	0	0	0	116	69	66	7.7	7.9	7.8	85	18	0.8	187	44	89	75	5.5	0
2	55	19.7	70.5	1	1	2	0	0	0	126	78	46	7.2	7	7.1	98	19	0.78	180	35	60	105	5.8	1
2	43	20.8	72	1	1	1	0	0	0	107	69	62	6.7	6.9	6.8	80	8	0.56	132	36	45	75	5	0
1	52	28.1	101	1	1	1	1	0	1	135	90	72	6.8	6.7	6.75	88	21	1.16	241	124	50	162	5.5	1
1	66	20.3	86	2	2	1	1	0	1	130	82	52	9.3	9.4	9.35	100	10	0.99	186	74	62	102	5.3	1
1	53	23.5	80	2	1	1	0	0	1	117	74	59	7.1	7.2	7.15	82	21	1.07	267	86	41	215	5.3	1
2	53	23.2	86	1	1	1	0	0	1	126	86	75	7.3	7.4	7.35	95	14	0.81	292	50	79	191	5	1
1	61	21.3	81.5	2	1	1	1	0	0	116	89	71	10.1	9.3	9.7	90	15	1.05	179	71	45	112	5.4	0
1	69	22.0	90	1	1	1	1	0	0	152	101	70	9.6	8.6	9.1	111	13	0.9	166	74	65	95	5.4	0

2	68	24.6	95	1	1	1	0	0	1	139	79	55	10.2	9.8	10	110	14	0.75	178	69	63	102	5.9	1
1	60	25.3	88	1	2	1	0	0	0	108	69	66	7.5	7.5	7.5	101	12	0.81	184	64	45	117	5.6	1
2	48	22.9	78	1	1	1	0	0	0	105	67	59	6.5	6.8	6.65	70	14	0.7	190	44	51	126	5.2	1
2	69	28.4	101.5	1	1	1	1	0	1	128	74	71	8.1	8.4	8.25	96	11	0.74	178	100	66	97	6	1
1	48	27.2	89.6	2	2	2	0	0	0	121	79	71	6.2	6.8	6.5	92	11	0.88	183	105	52	109	5.1	0
1	75	29.7	102.5	2	1	1	1	1	1	146	95	73	12.1	13.1	12.6	105	29	1.29	217	120	49	149	7.1	1
2	60	31.3	103	1	1	1	1	0	1	119	75	70	9.1	8.6	8.85	100	19	1.09	256	430	41	140	5.7	1
1	68	25.6	99	2	1	2	1	1	1	150	96	90	10.9	10.9	10.9	237	17	1.03	185	97	51	122	7.6	1
1	54	20.9	77	1	1	1	0	0	1	115	78	57	7.2	7.1	7.15	92	17	0.89	167	60	69	83	5.4	0
2	48	20.7	72.5	1	1	2	0	0	0	100	63	49	7.5	7.5	7.5	80	12	0.69	190	58	52	114	5.1	1
1	70	22.5	78	2	1	1	1	0	1	146	88	69	9.6	9.1	9.35	75	10	1.03	217	49	61	134	5.4	1
1	53	21.5	87.5	2	1	2	0	0	1	116	80	65	7.4	7.3	7.35	103	9	0.74	221	270	33	132	5.8	1
2	64	25.1	89.5	1	1	1	0	0	1	136	88	71	8.2	8.4	8.3	107	14	0.8	316	116	76	216	6	0
1	37	21.2	77	2	2	2	0	0	0	114	71	59	7.1	7.1	7.1	95	11	0.93	188	65	82	98	5.3	0
1	44	21.3	77	2	1	1	0	0	1	104	77	75	8.9	8.5	8.7	80	16	0.83	267	147	68	169	5.4	0
1	54	24.4	86.3	1	2	1	0	0	0	109	78	80	6.3	6.1	6.2	87	19	0.89	191	58	85	100	5.3	1
2	55	22.1	77.5	1	1	1	0	0	1	124	78	69	7.6	7.5	7.55	87	9	0.84	244	72	59	171	5.6	1
1	47	29.6	103.2	2	1	1	1	1	1	132	87	84	6.8	5.9	6.35	197	14	0.85	126	149	34	78	8.1	0
1	56	22.8	84.5	2	1	1	0	0	1	134	79	71	8.8	8.7	8.75	93	16	0.88	282	85	63	215	6	1
2	34	23.3	82	2	1	1	0	0	0	105	62	51	7.2	7.2	7.2	78	11	0.72	207	91	44	148	5.3	0
1	51	27.5	103.8	2	2	1	1	0	0	118	87	75	8.5	8.5	8.5	119	16	0.92	171	147	49	104	5.6	0
2	58	20.8	73.5	1	1	1	0	0	0	107	70	65	7.7	7.8	7.75	92	10	0.57	182	83	56	117	5.6	0
1	57	22.1	83	2	1	1	1	1	0	136	91	59	8.9	8.5	8.7	131	14	0.97	167	153	44	105	5.9	1
2	69	24.5	91.2	1	1	2	1	0	0	143	97	102	9.1	8.9	9	93	12	0.82	204	188	51	115	5.7	1
1	56	29.1	100	1	1	1	1	0	1	128	87	70	8.9	8.9	8.9	98	12	1.16	248	163	48	183	5.4	1
1	41	27.7	92	2	1	1	0	0	1	121	83	78	6.9	7	6.95	90	21	0.83	183	99	33	141	5.4	1
2	55	28.3	99	2	1	1	1	1	1	128	91	69	8.1	7.6	7.85	147	13	0.71	161	185	52	86	7.6	0
2	47	20.6	78	1	1	1	0	0	0	97	63	59	6.3	6.3	6.3	79	17	0.88	198	56	59	131	5.4	0
2	52	19.1	72.7	1	1	1	0	0	1	117	77	77	7.5	7.4	7.45	85	15	0.62	163	119	39	102	5.5	1

2	70	27.8	100.5	1	1	1	1	1	1	148	82	68	10.5	11	10.75	209	13	0.6	158	151	38	91	11.4	1
2	48	36.0	105.5	1	1	1	1	0	1	148	89	74	7	7.2	7.1	107	9	0.55	219	108	46	171	5.7	1
1	50	30.9	114	2	1	1	1	0	1	137	94	82	9.4	8.8	9.1	106	18	1.05	177	125	38	128	5.8	1
1	51	29.7	99.2	1	2	1	1	0	1	118	84	52	7	7.1	7.05	109	18	1.12	207	266	41	126	5.9	1
2	58	27.6	88.5	1	1	1	1	0	0	120	69	48	8.9	8.9	8.9	95	25	0.65	201	65	61	132	5.8	0
1	46	23.2	85.5	1	1	1	0	0	0	117	81	60	7.4	7.4	7.4	90	13	0.79	189	93	41	143	5.3	1
1	59	25.3	86.5	1	2	2	0	0	1	130	80	52	7.3	7.2	7.25	106	15	0.84	151	88	57	84	5.4	0
1	64	27.1	97.5	1	1	1	1	0	1	155	106	65	9.8	9.7	9.75	98	14	0.99	239	139	43	174	5.6	0
1	51	28.3	102	1	1	2	1	0	0	123	74	53	7.4	7.2	7.3	93	16	0.85	174	89	57	99	5.4	0
1	58	24.5	84	2	1	1	0	0	1	131	87	67	8.9	8.1	8.5	98	10	0.84	291	326	52	185	5.3	1
1	66	21.1	79.5	1	1	1	0	0	0	117	78	61	8.5	8.4	8.45	107	12	0.73	209	50	61	140	5.5	1
1	61	36.1	121	1	1	1	1	1	0	147	92	75	9.2	9.2	9.2	157	17	0.95	214	105	55	134	5.9	1
2	52	22.7	84	1	1	1	0	0	1	116	71	70	8.3	8.1	8.2	96	11	0.72	280	67	44	233	5.7	1
1	58	25.1	87.7	1	1	2	0	0	1	123	86	59	6.9	6.7	6.8	92	16	0.85	145	79	47	91	5.4	1
1	62	22.7	88	2	2	1	1	0	0	148	96	65	8	7.7	7.85	88	14	1.19	216	91	52	148	5.5	0
2	72	24.5	86.1	1	1	1	1	1	1	156	84	49	9.5	9.3	9.4	115	17	0.79	187	229	66	101	6.4	1
1	58	26.1	91	1	1	1	1	0	0	107	71	53	8.3	7.9	8.1	105	18	1.03	206	101	57	139	5.8	0
2	76	17.7	70.5	1	1	1	0	0	1	118	61	65	10.6	10.7	10.65	84	14	0.79	211	68	70	140	5.8	1
1	77	24.7	95	2	1	2	1	0	1	130	71	62	11.7	17.1	14.4	100	16	1.01	115	80	56	54	5.7	1
1	33	31.8	113	2	1	1	0	0	1	129	81	67	6.5	6.2	6.35	103	12	0.87	179	121	35	130	6.4	0
1	47	22.5	84.8	2	1	1	1	0	0	117	84	61	6.7	6.7	6.7	95	11	1.11	182	84	66	100	5.6	1
1	47	27.7	94.2	1	2	1	1	1	1	134	90	69	7.6	7.1	7.35	161	15	0.88	255	237	44	183	11.9	1
1	61	26.7	94	2	1	1	1	1	1	125	80	56	8.4	8.6	8.5	173	25	1.07	262	246	33	191	8.4	1
1	51	23.6	81	1	1	1	0	0	1	119	82	71	7.2	7.3	7.25	98	11	0.86	243	150	62	161	5.6	1
1	72	24.6	89	1	1	1	1	1	1	132	81	71	10.7	10.1	10.4	193	19	0.94	174	101	46	123	7.9	0
2	60	21.9	78.5	1	1	1	0	0	0	103	67	59	7.9	7.7	7.8	80	15	0.66	124	63	86	30	5.4	0
1	64	22.6	83.5	1	1	1	1	0	0	120	82	70	7.8	7.8	7.8	93	25	0.96	201	108	62	130	5.3	0
2	48	20.2	75	1	1	1	1	0	1	134	91	75	7.1	7.1	7.1	93	15	0.53	222	315	53	126	5.2	0
1	51	25.0	90	2	2	1	1	0	1	135	98	61	8.2	8.2	8.2	111	16	0.85	182	99	58	112	5.9	1

2	63	24.0	85.7	1	1	1	1	0	1	141	92	77	9.3	8.5	8.9	77	13	0.62	224	65	89	126	5	1
1	47	24.8	90.1	2	1	1	0	0	1	114	78	80	7.4	7.4	7.4	113	15	0.89	188	146	34	144	5.6	1
1	57	29.8	99	1	2	1	1	1	1	129	81	84	8.1	7.8	7.95	136	19	0.95	167	188	52	100	6.6	1
1	43	24.2	88.5	1	1	1	1	0	0	138	100	77	7.9	7.5	7.7	98	11	0.76	239	161	66	152	5.2	0
2	69	23.6	85.3	1	1	1	1	1	1	132	75	66	8.1	8.2	8.15	131	13	0.49	166	174	52	80	6.4	1
1	53	24.7	92	1	1	1	0	0	1	117	84	62	7.8	7.5	7.65	103	18	1.19	237	72	54	172	5.2	1
1	59	22.2	88	2	2	1	1	0	1	136	95	71	11.6	10.3	10.95	114	17	1.13	212	232	49	137	5.1	0
2	64	23.3	90	1	1	1	1	0	0	127	80	84	9.1	8.8	8.95	81	21	0.89	211	43	65	150	5.4	0
2	61	22.5	86	1	1	1	1	0	1	141	93	66	7.9	7.8	7.85	80	14	0.65	170	43	73	101	5	1
1	68	22.4	85	1	1	1	1	0	1	120	88	68	7.9	8.4	8.15	99	18	0.98	226	61	62	161	5.4	0
2	63	24.2	93.4	1	1	1	0	0	0	120	82	66	8.2	7.9	8.05	90	17	0.61	181	102	49	118	5.8	1
1	43	22.6	90.5	1	1	1	1	0	0	143	81	74	6.8	6.6	6.7	97	14	0.91	222	63	55	159	5.1	0
2	37	23.7	89.2	1	1	1	0	0	0	119	70	68	7.2	7.3	7.25	95	13	0.71	198	103	46	140	5.4	0
2	61	23.1	87	1	1	1	0	1	1	120	81	63	9.3	9.2	9.25	124	15	0.63	183	192	39	140	6.5	0
1	55	24.4	89.5	1	2	1	0	0	1	114	71	79	7.6	7.6	7.6	93	14	0.98	239	74	62	168	5.3	1
1	44	25.9	94	1	1	2	0	0	0	125	83	83	6.1	6.1	6.1	104	19	0.84	198	91	49	144	5.2	1
2	46	20.5	86.7	1	1	1	0	0	0	110	62	53	7.4	7.3	7.35	87	14	0.74	204	67	41	153	5.4	1
1	45	27.7	97	2	1	1	0	0	0	132	81	60	6.8	6.7	6.75	104	13	0.95	199	106	44	142	5.7	1
2	55	22.6	84.5	1	1	2	1	0	1	148	97	84	9.8	9.3	9.55	98	9	0.79	281	96	71	201	5.6	1
1	52	20.1	76.8	1	1	2	0	0	1	122	73	56	7.1	7.1	7.1	95	14	0.92	240	94	60	164	5.4	1
2	66	25.5	95	1	1	1	0	0	1	126	82	56	8.9	8.7	8.8	96	16	0.66	238	94	69	167	5.4	0
1	43	24.6	89.1	2	1	1	1	0	0	128	91	74	7.7	7.3	7.5	111	11	0.84	205	108	47	148	5.8	0
2	68	21.2	74	1	1	1	1	1	1	129	74	68	7.8	7.7	7.75	131	22	0.84	132	65	68	63	7	1
1	44	23.7	87.6	2	2	1	0	0	0	115	73	51	5.9	5.8	5.85	89	14	1.03	164	158	47	101	5	1
1	70	21.8	80.2	1	1	1	0	0	0	107	72	52	9	9.2	9.1	106	20	1.16	206	99	48	152	5.4	1
1	50	27.0	92	1	2	2	0	0	1	108	64	64	6.5	6.7	6.6	101	15	0.94	201	111	41	146	5.3	1
1	59	22.2	82.6	2	1	1	0	0	0	130	89	73	8.3	8.4	8.35	86	15	0.89	221	123	44	154	5.4	1
2	57	22.1	83.5	1	1	2	0	0	0	121	76	50	7.5	7.1	7.3	93	13	0.61	212	82	45	154	5.4	0
1	65	27.2	95	1	1	1	1	0	0	129	84	70	8.3	8.6	8.45	112	20	0.93	198	109	40	148	5.6	0

2	47	21.9	80	1	1	1	0	0	0	117	79	83	7.7	7.1	7.4	93	7	0.64	208	87	82	118	5.1	1
2	56	24.2	84	1	1	1	1	1	1	135	85	95	8.1	8.3	8.2	117	13	0.6	138	76	58	80	6.6	0
1	50	26.3	88.6	2	1	1	0	0	0	109	71	51	7.2	7.1	7.15	94	14	0.99	187	95	72	112	5.1	1
1	45	21.5	75.1	2	1	1	1	0	1	131	92	75	6.9	7.2	7.05	84	11	0.87	224	170	45	162	5.3	1
1	52	23.0	90.8	1	2	1	0	0	1	120	75	60	7	6.9	6.95	107	22	1.07	179	89	66	113	5.5	1
2	43	20.8	78	1	1	1	0	0	0	117	78	66	7.5	7.4	7.45	97	10	0.81	183	82	48	121	5.4	0
1	48	28.4	102.2	2	1	1	0	1	1	120	80	65	7.4	7.3	7.35	152	11	0.74	139	186	34	84	5.8	1
2	56	23.4	82	1	1	1	1	0	0	153	108	63	8.5	8.3	8.4	89	20	0.69	207	103	87	112	5.6	0
1	53	28.3	93	1	2	2	1	0	1	129	92	76	7.8	7.5	7.65	101	18	0.75	230	78	79	152	5.6	1
1	36	21.0	87.5	2	1	1	0	0	0	124	80	76	6.6	6.7	6.65	101	14	0.79	154	117	54	88	5.1	1
1	57	23.1	86.8	1	2	2	0	0	0	121	86	73	7.7	7.8	7.75	104	15	0.84	173	103	54	115	5.4	1
2	45	21.4	76	1	1	1	0	0	0	120	71	88	7.6	7.4	7.5	92	13	0.66	154	100	52	94	5	0
1	44	30.3	99	1	1	1	0	0	0	123	79	78	6.9	6.7	6.8	91	10	0.83	185	117	47	129	5.6	1
2	54	24.5	82	1	1	1	0	0	1	121	80	70	8	8	8	102	17	0.79	237	71	66	162	4.8	1
1	52	24.1	80	2	2	1	0	0	1	126	88	67	8.2	8	8.1	98	11	0.85	213	201	49	141	5.3	1
2	50	21.4	82.2	1	1	1	0	0	0	126	83	60	7.7	7.7	7.7	82	14	0.75	208	57	62	138	5.3	1
1	52	23.4	86	2	2	1	0	0	1	117	89	67	8.2	8	8.1	110	14	0.8	182	243	39	116	5.2	0
1	49	24.7	91	1	1	1	1	0	1	131	95	58	8.8	8.4	8.6	88	13	0.99	196	277	34	124	5.3	1
1	33	24.8	87	2	1	1	0	0	0	127	77	58	6.1	6.1	6.1	84	17	0.9	176	112	48	113	4.9	0
1	51	25.3	88	2	1	1	0	0	0	110	78	72	7.2	7.3	7.25	82	11	1.06	158	40	41	116	6.1	1
1	50	36.7	120	1	1	1	1	1	1	160	110	76	7.8	7.7	7.75	111	10	0.7	142	90	34	104	7.5	1
2	66	33.9	112.7	2	1	1	1	1	1	134	70	65	8	7.7	7.85	85	24	0.45	163	233	43	96	5.7	0
1	67	24.2	103	1	1	1	1	1	1	127	86	66	10.3	10.5	10.4	138	16	0.95	242	411	36	144	9.9	1
1	63	25.6	96.1	1	1	1	1	1	1	166	99	75	10	10	10	115	12	0.66	161	121	36	118	11.1	1
1	72	23.2	89	2	2	1	0	0	0	124	77	77	10.8	10.5	10.65	73	15	1.01	191	75	66	119	5.1	0
2	46	23.3	83.7	2	2	1	0	0	0	106	72	60	8.9	8.6	8.75	103	15	0.76	152	108	43	102	5.4	0
1	48	27.4	98	2	2	2	1	0	1	132	96	56	9.7	9.6	9.65	102	25	1.02	196	250	36	129	5.7	0
2	24	18.3	66.1	1	1	1	0	0	0	105	81	65	6.5	7	6.75	82	11	0.7	141	59	66	76	4.8	0
1	48	25.4	84	2	1	1	1	0	1	135	92	89	6.1	6.1	6.1	125	14	0.94	231	61	65	164	5.4	1

1	45	35.1	107.8	1	1	1	1	0	1	138	99	78	6.5	5.9	6.2	92	21	1.19	213	134	32	169	5.5	1
2	54	26.3	84.6	1	1	1	0	0	0	126	69	72	6.9	6.9	6.9	94	10	0.61	204	89	65	132	5.3	1
2	36	22.1	76.5	1	1	1	0	0	0	119	77	65	6.2	6.1	6.15	82	11	0.56	175	102	61	106	4.9	1
1	40	22.1	85.7	1	1	1	0	0	0	125	86	68	6.4	6.6	6.5	102	11	0.84	169	90	64	98	5.1	0
2	43	24.8	87	1	1	1	0	0	0	110	77	71	7.3	7.3	7.3	91	14	0.54	195	46	82	112	5.4	0
2	58	22.7	80	1	1	1	1	0	1	146	92	47	7.1	7	7.05	86	12	0.59	220	96	42	172	5.4	1
1	66	26.0	95	1	1	1	1	1	1	131	79	62	7.4	7.7	7.55	136	17	0.78	204	98	82	117	6.7	1
1	41	29.1	101	2	2	1	0	0	1	126	87	76	6.7	6.7	6.7	114	13	1.01	230	358	52	135	5.9	0
1	61	28.4	102.1	1	1	1	0	0	0	131	81	56	8	7.9	7.95	100	12	0.89	194	159	47	133	5.8	0
1	36	23.7	88.7	1	1	1	0	0	1	118	71	61	7.6	7.3	7.45	90	15	1.03	242	163	42	182	5.4	1
1	36	28.0	105.7	1	1	1	0	0	1	118	70	79	7.9	8.1	8	100	16	0.89	139	144	24	103	5.1	1
2	74	22.2	83.7	1	1	2	0	0	0	132	76	57	8.9	8.8	8.85	94	30	0.66	193	40	55	140	5.7	1
1	39	22.3	77.5	2	1	2	0	0	0	120	69	45	7.9	7.7	7.8	86	13	1.14	175	131	44	115	5.2	1
1	55	25.1	92.8	1	1	1	0	0	0	124	76	65	7.1	7.1	7.1	93	15	1.03	183	88	56	122	5.4	1
1	76	21.9	87	1	1	1	1	0	1	123	74	50	9.8	9.9	9.85	104	24	1.14	162	86	46	114	5.3	0
1	45	23.8	77.7	2	1	1	0	0	0	118	87	53	8.4	8.7	8.55	93	16	0.79	173	81	54	108	5	0
1	38	29.2	100.5	2	1	1	0	0	0	127	80	69	6.5	6.2	6.35	102	13	0.78	220	176	56	148	5.6	1
1	41	24.6	84.5	2	1	1	0	0	1	115	75	70	7.1	7	7.05	97	14	0.77	248	137	43	175	5.7	1
2	56	22.4	83.8	1	1	1	0	1	1	121	64	61	7.3	7.2	7.25	142	13	0.67	222	115	55	147	7	1
1	40	22.6	73.7	1	1	1	0	0	0	108	75	76	7.2	7.2	7.2	94	10	0.92	188	61	68	116	5.2	0
1	51	25.5	91	1	1	1	0	0	0	119	82	59	6.7	6.8	6.75	87	12	0.81	188	86	58	120	5.2	1
1	52	25.2	88.5	1	1	1	0	0	0	107	76	69	7.3	7.6	7.45	97	10	0.79	195	145	65	112	5.3	0
1	49	22.3	84.1	1	2	1	0	0	0	122	83	66	7.8	7.6	7.7	91	13	1	233	84	69	152	5.1	0
1	38	22.5	84	1	1	1	0	0	0	115	72	61	7.8	7.6	7.7	90	11	0.84	182	118	45	128	5.3	0
1	38	30.9	118.7	2	1	1	0	0	0	124	76	66	5.7	5.7	5.7	103	11	0.82	204	77	51	141	5.4	0
1	30	26.5	93	2	1	1	1	0	0	137	94	58	7.6	7.5	7.55	99	14	0.9	230	186	73	135	5	0
2	49	26.4	89	1	1	1	1	0	1	135	91	69	6.6	6.5	6.55	121	15	0.55	217	119	43	164	5.8	1
2	75	19.9	89	1	1	1	1	1	1	134	82	78	12.7	11.9	12.3	109	15	0.55	160	122	98	60	7.2	1
2	51	20.0	72	1	1	2	1	0	0	121	83	56	7	7	7	94	13	0.73	192	37	70	123	5.1	0

1	63	25.3	93	1	1	1	1	1	0	131	78	47	9.4	8.8	9.1	117	19	0.99	202	111	46	127	6.5	1
1	68	22.4	75.5	1	2	2	0	1	0	139	84	57	8.2	8	8.1	130	18	0.79	236	91	73	159	6.4	1
2	48	38.6	109	2	1	1	1	0	0	150	90	68	5.5	5.9	5.7	93	13	0.84	239	133	50	151	5.7	1
1	57	36.0	123	1	1	1	1	1	1	130	88	58	9.2	8.7	8.95	161	16	0.95	225	246	45	134	8.9	1
1	81	26.9	96	2	2	1	0	0	1	133	83	49	9.4	9.6	9.5	113	20	1.07	134	80	71	58	6.1	1
1	77	23.6	85	1	1	1	1	1	1	147	83	90	11.6	11.8	11.7	232	28	1.01	157	69	68	76	9.4	0
1	54	25.5	93	2	2	1	1	0	0	137	94	71	8.6	8.5	8.55	98	14	1.01	163	104	56	78	5.4	0
1	62	26.3	101	2	2	2	0	0	1	123	71	46	7.7	7.8	7.75	89	12	0.97	266	169	56	169	5.6	0
2	56	22.9	85	1	2	1	0	0	1	111	65	51	6.7	6.7	6.7	101	9	0.63	333	42	108	200	5.4	1
1	53	25.3	92	1	2	1	0	0	0	122	80	66	7.4	7.3	7.35	104	18	0.83	202	173	48	122	5.5	0
2	42	21.2	76.8	1	1	1	0	0	1	110	73	68	6.9	6.8	6.85	111	11	0.91	240	121	45	160	5.9	1
1	58	24.7	89.5	2	1	1	1	0	0	128	83	66	8.1	8.2	8.15	109	8	0.89	212	187	53	122	6.1	1
2	40	29.2	91.5	1	1	2	0	0	0	136	84	62	6.7	6.7	6.7	96	13	0.72	231	119	52	152	5.9	0
2	52	31.6	108	2	2	1	0	0	0	108	77	69	7.8	8.2	8	98	14	0.64	187	79	57	116	6.1	1
1	59	26.0	100.5	2	1	1	1	0	0	151	89	61	9.3	9	9.15	97	22	1.08	227	90	75	132	5.7	1
2	54	25.5	87.5	2	1	1	0	1	0	123	79	88	8.3	8.2	8.25	257	20	0.71	227	94	56	149	11.2	1
1	53	27.8	80	2	1	1	1	0	1	139	87	67	8.2	8.1	8.15	103	15	1.07	232	206	41	144	6.1	1
2	65	24.2	90	1	1	1	1	1	1	148	89	73	9	8.6	8.8	167	17	0.68	277	138	67	173	7.6	0
1	54	23.2	79.5	2	1	1	1	1	1	146	94	52	8.5	8.3	8.4	112	13	0.93	178	171	42	107	6	1
1	41	34.5	112	2	1	1	0	0	1	108	77	71	7.7	7.3	7.5	89	13	0.83	163	88	36	112	6.1	0
1	55	26.1	89	2	2	1	1	1	0	143	103	78	9.5	9.2	9.35	141	11	0.81	200	63	73	125	7.4	1
2	49	26.1	87	1	1	1	0	0	0	107	73	60	7.9	8.1	8	107	22	0.6	186	45	71	100	5.6	1
1	44	24.1	89	1	1	1	0	0	1	128	85	72	7.9	7.8	7.85	94	19	0.89	162	212	33	98	5.7	0
1	50	29.2	108.5	1	1	1	1	1	1	136	93	55	8.3	8.4	8.35	155	16	0.82	158	173	39	92	7.3	1
2	57	17.6	66.3	1	1	1	0	0	0	128	85	66	7.7	7.7	7.7	91	14	0.69	229	56	103	120	5.7	0
1	40	30.4	101.5	1	1	1	1	0	1	140	76	67	6	6	6	114	16	1.22	234	217	50	147	5.6	1
2	41	22.4	81.5	1	1	1	1	0	0	142	92	57	6.5	6.7	6.6	92	13	0.81	184	38	81	94	5.3	0
1	70	24.5	93	2	1	1	0	1	1	106	89	66	10	10.1	10.05	111	16	1.03	176	117	53	107	6.4	0
1	53	25.9	93.6	1	1	1	1	1	1	104	68	64	6.8	6.9	6.85	104	28	1.49	138	87	42	83	6.5	1

1	68	26.4	90	2	1	1	1	0	0	146	78	60	9.4	9.1	9.25	112	12	0.85	193	60	50	129	5.7	1
2	64	28.4	95.5	1	1	1	1	1	1	133	83	70	9.4	9.3	9.35	124	15	0.59	164	82	61	88	7.5	1
1	51	27.7	95.3	2	1	2	1	0	0	127	89	54	8.6	7.9	8.25	100	26	1.15	183	145	48	106	5.4	0
1	41	30.7	100.5	2	2	1	1	0	1	152	103	93	8.1	10.5	9.3	114	15	1.01	293	367	43	169	6.1	0
1	61	23.3	84	1	1	1	0	0	0	120	83	59	6.9	7.3	7.1	100	14	0.84	168	111	50	90	5.4	0
1	39	23.9	92.5	2	2	1	0	0	0	120	75	54	7.7	7.7	7.7	88	17	1.03	218	92	70	127	5.4	0
1	72	25.5	91.5	1	1	1	1	1	0	138	89	55	11.2	11.4	11.3	141	18	0.77	208	94	51	138	8.1	0
2	61	19.9	77	1	1	1	0	0	1	119	75	70	7.8	7.7	7.75	94	12	0.62	292	82	59	209	6	0
2	54	23.3	84.5	1	1	1	0	0	0	123	87	78	8.4	8.3	8.35	103	16	0.66	233	73	58	136	5.5	1
1	49	25.4	94	1	1	1	1	0	0	121	84	67	8	7.6	7.8	105	16	0.99	198	67	51	136	5.4	1
2	40	21.8	77	1	2	1	0	0	0	110	72	65	6.7	6.6	6.65	76	14	0.94	175	77	65	94	5.3	1
1	45	24.8	90.5	1	1	1	0	0	1	119	83	78	6.1	6	6.05	50	17	1.08	262	62	68	177	5.2	1
1	58	27.6	93.5	1	1	1	0	0	1	125	87	74	7.6	7.5	7.55	88	10	0.75	163	160	34	96	5.4	0
1	55	25.0	91	2	2	1	1	1	1	142	96	59	9.7	9.7	9.7	127	13	0.77	134	275	43	60	5.8	0
2	58	21.8	77.5	1	1	1	0	0	0	112	66	61	7.2	7.2	7.2	88	10	0.71	161	152	43	88	5.6	0
1	44	23.7	81	1	1	2	0	0	0	114	71	63	6.7	6.8	6.75	114	17	1.03	197	60	52	129	5.8	1
1	49	23.1	83	2	2	1	0	0	0	113	61	53	7.1	6.9	7	100	12	0.93	191	75	62	121	5.7	1
1	55	24.0	84	2	2	1	1	0	1	137	103	78	9	10.6	9.8	88	11	1.02	208	212	40	131	5.5	0
1	65	28.1	102.5	2	2	2	1	1	1	128	79	74	8.9	8.6	8.75	140	11	0.93	167	122	81	72	6.6	0
2	62	15.8	65	1	1	1	0	0	1	112	75	48	9.2	9.2	9.2	98	17	0.61	258	65	111	138	5.3	0
2	48	27.7	109	1	1	1	0	0	0	127	87	70	7	6.9	6.95	93	13	0.71	155	54	47	98	6	1
2	70	29.2	74	1	1	1	1	1	1	150	90	76	11.4	11.5	11.45	208	6	0.47	130	78	34	78	10.8	1
2	24	27.9	96	1	1	1	0	0	0	126	75	67	5.5	5.4	5.45	99	7	0.51	145	64	51	81	5.5	1
1	56	25.0	90.5	1	2	1	0	0	1	126	88	60	8.1	7.3	7.7	92	15	0.88	248	328	39	141	5.4	1
1	64	20.3	72	2	2	1	1	1	0	143	81	66	8	7.9	7.95	118	9	0.76	169	63	102	65	5.9	1
2	63	25.9	89	1	1	1	1	1	1	141	98	68	9.2	8.8	9	140	27	0.68	262	133	52	173	7.1	1
1	39	25.7	89.5	1	1	1	0	0	0	122	73	46	7.6	7.6	7.6	89	22	1.03	198	71	66	127	5.4	0
1	65	24.8	93	2	2	1	1	0	1	129	91	64	8.7	9.7	9.2	96	14	0.69	150	87	55	87	5.7	0
1	54	28.1	97	1	2	1	1	0	0	145	109	68	7.8	7.1	7.45	94	11	0.88	158	89	58	78	5.7	1

1	54	23.5	85	2	1	1	1	0	1	142	92	70	10	9.4	9.7	102	15	0.91	196	202	59	110	5.7	1
1	51	24.5	93.5	2	1	1	1	0	0	135	94	74	7.1	7.1	7.1	107	14	0.95	157	81	40	101	5.3	0
1	47	23.0	80	1	1	1	0	0	1	116	66	53	7.8	7.8	7.8	98	13	0.94	128	61	37	80	5.9	0
1	45	21.1	76	2	2	1	0	0	1	126	83	92	6.8	7.1	6.95	116	10	0.58	284	67	98	181	5.6	1
2	42	22.7	78	1	1	1	0	0	0	129	86	65	6.9	6.8	6.85	105	11	0.61	174	45	76	93	5.7	1
1	55	21.2	81	1	1	1	0	0	0	114	74	71	6.7	6.5	6.6	94	12	0.99	187	44	53	124	5.4	0
1	63	28.1	98.5	2	2	2	1	1	1	120	93	64	6.3	6.1	6.2	127	15	1.06	178	80	56	113	6.5	1
2	59	21.9	83	1	1	1	1	0	0	146	87	56	8.8	8.2	8.5	97	12	0.73	219	39	80	135	5.9	1
1	66	25.8	91	1	1	1	0	0	0	138	88	69	9	8.8	8.9	110	10	0.83	181	52	49	128	5.4	1
1	46	28.4	93	1	1	1	1	1	0	124	76	83	7.8	8	7.9	126	14	1.07	204	185	40	135	6.2	1
1	55	21.7	83	2	1	2	1	0	1	134	90	71	9.4	9.2	9.3	113	17	0.82	249	131	61	171	5.6	0
1	41	24.1	87	2	1	1	1	0	1	134	92	64	6.4	6.3	6.35	95	11	0.91	151	184	31	97	5.3	1
1	34	25.2	90.5	1	1	1	0	0	0	134	89	62	7.4	7.5	7.45	97	13	0.95	216	147	51	133	5.3	1
2	52	23.6	79.5	1	1	1	0	0	0	125	76	56	7.1	7.3	7.2	99	13	0.72	164	41	69	97	5.9	0
1	41	22.8	82	2	2	1	0	0	1	125	86	79	6.4	6.2	6.3	93	12	0.78	195	213	50	107	5.4	1
1	57	22.9	83.5	2	2	2	0	0	1	126	86	55	8.7	8.3	8.5	105	20	0.81	214	358	45	127	5.3	0
1	48	21.8	84	1	2	1	0	0	0	123	77	65	7.2	7.5	7.35	88	19	0.91	161	63	51	98	5.4	1
1	56	30.1	97	1	1	1	0	0	1	133	72	60	5	4.9	4.95	89	12	0.81	203	363	39	102	5.4	1
2	50	25.1	92	1	1	1	0	0	0	127	83	72	6.7	6.6	6.65	100	9	0.73	201	43	69	126	5.8	1
1	58	23.8	85	1	1	1	0	0	0	93	65	53	7.8	7.7	7.75	99	16	0.95	200	87	52	128	5.5	1
2	65	30.5	105	1	1	1	1	1	1	138	89	73	10.1	10	10.05	119	10	0.75	155	132	35	98	6.4	1
1	49	25.0	94	2	1	1	0	0	1	122	88	67	7.1	6.9	7	114	8	0.98	206	102	35	152	5.7	1
1	53	30.7	113	1	1	1	1	0	0	159	103	68	8.4	8.2	8.3	101	19	0.9	117	42	47	66	5.7	1
2	70	25.6	91	1	1	1	1	0	1	146	78	61	8.9	8.7	8.8	114	19	0.51	267	91	43	221	6.2	0
1	56	27.0	94	2	2	2	0	0	1	119	77	67	7.8	7.7	7.75	116	10	0.85	246	332	43	121	5.7	1
1	50	23.6	87	2	2	2	0	0	0	121	79	53	7.4	7.2	7.3	92	11	0.85	189	96	49	126	5.4	1
2	66	29.3	103	1	1	1	0	1	1	136	80	57	7.1	6.8	6.95	125	17	0.64	355	152	58	261	6.9	1
1	71	21.1	72	2	1	1	0	0	0	132	89	70	9.3	9.1	9.2	93	20	1.05	164	53	66	92	5.3	1
1	55	27.0	88	1	1	1	1	0	1	136	85	55	7.4	7.1	7.25	103	14	0.88	193	215	38	119	5.8	1

1	55	22.4	78	2	1	1	0	0	0	111	73	58	9.1	8.9	9	95	21	1.03	210	124	54	140	5.7	1
1	42	24.7	87	2	1	2	0	0	0	119	77	53	7.8	7.9	7.85	99	14	0.98	208	58	69	138	5.7	0
1	55	18.2	73	2	1	1	0	0	0	110	76	65	8.2	8.1	8.15	105	12	1.01	171	96	61	99	5.4	1
1	65	24.6	83	1	1	1	0	0	0	118	83	60	7.2	7.4	7.3	105	11	0.85	182	81	44	125	5.8	1
2	64	21.2	76	1	1	1	0	0	0	129	82	63	6.2	6	6.1	58	12	0.78	235	106	88	130	5.2	1
1	47	24.8	88.5	1	2	1	0	0	0	123	79	58	8.2	8.3	8.25	111	9	0.99	205	98	43	148	5.9	1
1	62	23.6	83	2	1	1	0	0	1	121	81	69	8.6	8.5	8.55	103	12	0.94	145	77	49	93	6	0
2	55	22.5	79	1	1	1	0	0	1	126	82	57	6.9	6.6	6.75	93	12	0.68	182	122	60	108	5.9	0
1	47	26.4	90	1	2	1	1	0	0	130	96	65	6.8	6.4	6.6	120	15	0.85	213	93	76	134	5.4	0
1	39	33.0	71	2	1	1	1	0	0	157	102	90	8.4	8	8.2	109	11	0.89	209	121	73	127	5.8	0
1	52	21.8	79	1	1	1	0	0	0	122	80	68	8.1	8.1	8.1	92	13	0.71	185	89	73	103	5.4	0
1	58	19.9	78.5	1	1	1	1	1	0	121	90	81	7.6	7.4	7.5	129	10	0.74	190	51	58	130	6.1	1
2	68	20.8	81	1	1	1	1	0	0	154	86	73	9.5	9.5	9.5	95	11	0.51	168	74	55	102	5.9	0
1	33	22.5	84	2	2	1	0	0	0	112	64	46	7.5	7.5	7.5	89	18	1.03	181	69	66	112	5.6	1
2	35	21.5	76	1	1	1	0	0	0	91	67	82	6.3	6.2	6.25	93	11	0.57	152	38	64	88	5.4	0
1	43	27.2	100	2	1	1	1	0	1	135	94	57	7.2	6.8	7	111	13	0.93	162	233	40	94	5.7	1
1	41	30.0	105	2	2	1	1	0	0	126	85	82	6.2	7	6.6	102	11	0.77	199	182	44	137	5.8	0
2	74	25.2	87	1	1	1	1	0	1	152	93	63	9.5	8.7	9.1	96	22	1.01	151	74	62	77	5.8	0
1	49	25.0	85.5	1	1	2	1	0	0	134	87	61	7.3	7	7.15	96	13	0.87	207	63	60	134	5.8	0
1	43	27.0	94	1	1	1	0	0	1	128	86	58	6.8	6.8	6.8	102	8	0.87	166	160	55	97	5.9	0
1	47	25.5	84	1	2	1	0	0	0	110	74	59	7.2	7.2	7.2	94	10	0.85	160	58	65	91	5.2	0
2	60	20.1	76.5	1	1	1	1	0	1	137	88	69	9.4	9.3	9.35	87	11	0.67	273	82	78	181	5.6	1
1	60	24.4	82	2	1	1	0	0	1	122	77	67	8.7	8.3	8.5	92	15	1.19	250	129	51	176	5.5	0
2	50	29.0	91	1	1	2	1	0	0	115	70	60	7.4	7.3	7.35	98	12	0.8	187	102	46	125	5.9	1
2	58	20.2	74.5	1	1	1	0	0	0	88	53	64	7.5	7.3	7.4	86	19	0.72	188	95	55	109	5.7	1
1	41	23.7	83	1	1	1	0	0	1	114	82	72	6.9	6.8	6.85	103	15	1	222	91	55	161	5.6	0
1	56	26.7	86.5	1	2	1	1	0	1	136	83	57	7	7	7	103	13	0.89	112	38	81	43	6	1
1	53	26.5	91	1	1	1	0	1	1	124	89	69	7.7	8.2	7.95	146	13	1.01	143	191	37	89	6.4	0
2	50	29.6	96.5	1	1	1	1	1	1	145	91	72	9.4	7.8	8.6	126	10	0.66	147	88	56	75	6.8	1

1	54	26.5	92.5	1	1	1	0	0	0	111	77	57	7.8	7.9	7.85	98	18	1.07	198	179	40	96	5.4	0
2	56	23.1	83	1	1	1	0	0	0	121	77	59	6.8	7.2	7	88	15	0.68	175	50	79	95	5.9	0
1	56	24.3	84	1	1	1	0	0	1	120	79	67	7.1	7.2	7.15	94	15	0.81	263	52	46	199	5.6	0
1	61	26.0	93	2	2	1	1	0	1	155	101	61	10.1	9.5	9.8	111	16	1.05	188	481	46	77	6.2	0
1	60	26.2	90	1	1	1	1	1	1	132	86	77	7.1	7.7	7.4	127	17	0.96	146	107	53	84	6.6	1
1	43	26.1	94	2	1	2	0	0	0	116	78	63	7.7	7.5	7.6	100	13	0.86	217	105	52	150	6.2	0
1	53	22.2	83.5	2	1	1	1	0	0	126	81	70	8.2	8.2	8.2	87	15	0.97	192	166	49	116	5.5	1
2	68	28.5	100	1	1	1	1	0	1	126	90	64	7.6	8.1	7.85	101	19	0.83	181	74	73	105	5.9	1
2	48	21.2	78	1	1	1	0	0	0	124	80	56	7.1	7.1	7.1	95	10	0.8	168	44	60	107	5.5	1
2	54	19.9	70.5	1	1	1	0	0	1	119	76	55	7.4	7.4	7.4	82	12	0.64	285	109	89	181	5.7	0
1	46	26.2	88	1	1	1	0	0	1	122	86	51	7.5	7.4	7.45	93	11	0.97	228	183	41	166	5.8	0
1	56	20.4	79	2	2	2	0	0	0	122	76	63	8.5	8.4	8.45	96	12	1.09	176	130	40	119	5.4	1
1	65	23.4	85	2	2	1	0	0	1	111	78	64	7.3	7.2	7.25	88	10	0.92	163	64	62	98	5.5	0
1	67	21.6	86	2	1	1	1	0	1	141	96	84	8.6	8.3	8.45	100	13	1.03	218	118	36	163	5.7	0
2	66	20.3	77	1	1	1	1	0	1	126	83	62	9.3	8.8	9.05	103	8	0.64	193	91	60	122	6.2	1
1	58	23.2	88	1	1	1	0	0	0	133	85	49	6.3	6.7	6.5	97	8	0.83	200	46	89	110	5.4	1
1	49	25.7	88	1	1	1	1	0	0	121	86	68	8.1	7.6	7.85	99	8	0.86	229	100	57	154	5.4	0
2	46	20.8	79	1	1	2	1	0	0	139	94	59	6.5	7	6.75	97	13	0.76	155	60	48	96	5.3	0
1	50	23.0	86	2	1	2	1	0	0	140	90	63	7	7	7	101	6	0.86	204	118	67	126	5.6	0
1	55	22.2	84	1	1	1	1	0	0	125	93	67	8	7.9	7.95	101	16	0.89	191	65	49	138	5.8	0
2	67	26.0	87	1	1	1	1	0	1	154	92	64	8.2	7.4	7.8	96	13	0.57	251	92	71	164	5.9	1
2	57	36.6	111.5	1	1	1	0	0	1	134	81	60	7.4	7.4	7.4	83	12	0.81	248	66	91	154	5.5	1
2	58	19.1	76	1	1	1	0	1	1	122	83	79	8.2	8.1	8.15	248	13	0.72	259	126	48	167	10.6	0
2	55	20.8	74	2	2	1	1	0	0	120	82	75	8.2	11.4	9.8	90	9	0.52	221	117	68	142	5	1
1	41	25.7	90.5	2	1	2	1	0	1	133	91	77	7	6.6	6.8	94	10	0.92	222	116	34	170	5.8	0
2	52	24.4	81	1	1	1	0	0	1	132	82	64	8.9	7.2	8.05	104	12	0.73	193	255	34	126	5.6	1
1	53	23.8	90	1	2	1	0	1	0	131	80	66	7.2	7.2	7.2	131	14	0.76	146	71	56	88	6.4	1
2	70	25.9	90.5	2	1	1	1	1	1	126	82	65	11.5	9.3	10.4	129	15	0.95	200	188	79	98	6.9	0
2	68	21.0	79.5	1	1	1	1	0	1	132	79	66	7.8	7.4	7.6	111	8	0.7	185	66	91	93	6.2	0

1	68	24.3	97	2	1	1	1	1	1	126	93	76	11	13.1	12.05	149	21	1.25	183	185	41	122	6.1	0
1	45	22.2	84	2	2	1	1	0	0	156	107	67	6.9	6.8	6.85	90	9	1.07	175	78	83	89	5.2	1
1	49	21.1	84	1	1	1	0	0	0	115	73	58	7.7	7.6	7.65	93	12	0.9	178	112	53	115	5.4	1
1	57	25.6	89	1	1	1	1	0	1	132	94	57	6.7	6.6	6.65	103	11	0.87	167	141	55	89	5.6	0
1	55	29.4	102.5	2	1	1	1	0	0	137	90	66	8.1	8	8.05	111	13	0.75	221	105	51	152	5.7	0
2	66	23.5	88.5	1	1	1	1	0	1	131	85	81	7.5	7.6	7.55	100	12	0.94	174	328	51	80	6	1
1	53	25.0	88	2	1	1	1	0	0	176	120	86	9.5	8.9	9.2	94	16	1.05	209	159	56	128	5.3	1
1	55	25.9	89	1	2	1	1	0	0	157	99	75	6.3	6.6	6.45	108	16	1.02	173	141	42	106	5.5	1
1	62	22.2	81	1	1	1	0	0	1	119	77	73	7.9	7.6	7.75	109	20	0.91	174	77	54	114	6.1	1
1	55	25.1	95.5	2	2	1	1	0	0	139	96	68	7.4	7.1	7.25	92	12	0.87	137	169	44	80	5.7	0
1	54	24.8	86	1	2	2	1	0	0	140	76	45	5.7	5.9	5.8	83	9	1.1	189	75	63	123	5.4	0
2	68	26.8	92	1	1	1	1	0	1	147	91	66	8.4	8.1	8.25	93	18	0.57	242	82	50	181	5.6	0
1	48	24.3	86	2	1	1	1	0	1	123	92	60	9	8.3	8.65	100	10	0.96	247	94	72	165	5.7	1
1	53	23.9	87.5	2	1	1	1	1	0	121	80	61	7.6	7.7	7.65	130	14	0.75	134	97	56	77	6.6	1
1	45	25.4	92	2	1	2	0	0	1	123	77	74	7.2	6.8	7	92	15	1.13	210	222	48	129	5.4	1
2	62	27.3	90	1	1	1	0	0	1	133	70	58	7.5	7.6	7.55	114	13	0.55	189	96	79	108	6	0
1	65	33.6	110.5	2	2	2	1	0	1	158	106	64	8.4	8	8.2	121	21	0.82	162	153	59	94	6.2	0
2	44	20.4	76	1	1	2	0	0	0	134	67	62	7	7	7	104	11	0.71	162	93	69	80	5.5	0
2	61	22.8	77	1	1	1	0	0	1	136	87	66	8.7	8	8.35	84	12	0.63	211	208	42	132	5.4	1
1	64	23.6	94.5	2	1	1	1	1	1	152	96	93	10.4	10	10.2	112	20	0.95	250	176	63	161	7.5	1
1	42	30.1	105	1	1	2	1	0	1	144	89	54	5.4	5.4	5.4	93	12	1.24	280	231	54	164	5.7	1
1	45	26.2	96	1	1	1	0	0	1	122	84	67	7.5	7	7.25	99	14	1	240	110	68	155	5.5	1
2	50	22.4	82.5	1	1	1	0	0	0	132	84	86	7.1	7	7.05	93	8	0.58	195	29	81	126	5.6	0
1	59	25.9	91	1	1	1	0	0	1	108	84	95	6.1	6.2	6.15	97	16	0.98	263	63	65	197	5.4	0
1	47	24.0	87	2	2	1	0	0	0	135	83	71	7.5	7.3	7.4	94	13	0.84	219	51	76	143	5.3	0
1	55	24.2	83.5	1	2	1	1	0	0	124	90	69	6.9	7.1	7	106	10	0.78	182	71	54	119	6	0
2	54	24.8	89	1	1	1	0	0	0	136	70	60	7.1	7	7.05	91	11	0.77	198	83	51	141	5.7	1
1	42	42.0	133	2	1	2	1	0	1	150	79	60	6.5	6.6	6.55	101	6	1.16	165	111	34	116	5.3	1
1	52	21.7	80	1	2	1	1	1	0	140	88	62	7.7	7.8	7.75	146	11	0.89	166	106	75	77	6.3	1

1	56	23.0	85	2	2	2	0	0	0	126	87	63	8.5	8.1	8.3	97	15	0.97	184	86	56	114	5.5	1
1	57	23.4	86	2	1	1	0	0	0	112	78	70	7.9	8	7.95	80	23	0.85	193	77	54	130	5.4	1
1	54	24.2	84.5	2	1	1	1	0	1	141	91	53	9.3	8.8	9.05	109	18	0.96	239	104	48	178	5.8	0
1	63	21.0	78	1	1	2	0	0	1	107	72	62	8.3	8.1	8.2	95	13	0.92	185	218	49	103	5.4	1
1	56	21.0	78	1	2	1	1	0	0	132	93	81	7.7	7.5	7.6	115	12	0.94	217	97	86	134	5.8	1
2	62	22.9	87	1	1	1	0	0	1	111	72	72	7	7.1	7.05	94	11	0.74	168	85	61	102	5.5	1
1	43	26.0	89	1	1	2	1	0	1	141	97	55	6.7	6.6	6.65	90	11	0.75	191	262	50	104	5.4	0
1	56	25.4	96	1	1	1	0	0	0	120	82	56	7.4	7.4	7.4	95	14	0.99	173	91	62	102	5.8	0
1	40	22.7	82.5	1	1	2	1	0	1	132	87	77	7.3	7.2	7.25	96	12	1.15	183	220	39	117	5.7	0
2	74	23.5	85	1	1	1	1	1	1	126	72	71	10.4	10.2	10.3	130	17	0.77	139	167	34	82	7.1	1
1	54	25.4	100	2	1	1	1	1	1	140	86	74	6.9	7.1	7	142	13	0.69	130	135	35	80	6.8	0
1	57	26.0	93.5	1	1	1	1	0	1	130	96	61	7.1	7.3	7.2	89	19	1.07	171	231	39	82	5.4	0
2	59	18.0	68	1	1	1	0	0	0	117	83	65	7.9	8	7.95	104	14	0.62	205	79	92	116	5.6	0
2	64	35.2	105	1	1	1	1	0	0	139	82	73	8.8	7.9	8.35	92	11	0.82	201	150	49	128	5.9	1
1	48	23.8	86	1	1	1	0	0	1	116	82	73	7.6	7.7	7.65	100	10	0.81	191	226	38	121	5.9	0
1	59	18.5	65	2	1	1	0	0	0	109	67	60	9	9	9	85	16	0.97	131	77	51	74	5.6	1
1	42	22.4	86	1	1	1	0	0	0	111	78	64	7.9	7.7	7.8	92	16	1.06	185	36	59	136	5.8	1
2	57	16.9	68	1	1	1	0	0	0	118	65	54	8.2	8.4	8.3	91	11	0.76	195	82	83	106	5.8	1
1	52	31.2	113.5	1	1	1	1	0	0	165	108	51	6.5	6.7	6.6	106	18	0.94	183	39	63	124	5.4	1
2	54	27.6	83	1	1	1	0	0	0	118	67	50	7.3	6.2	6.75	101	15	0.76	194	88	63	120	5.8	1
1	56	23.7	82.5	1	1	1	1	0	1	124	94	65	7.8	8	7.9	90	23	1.11	143	56	49	96	5.5	1
2	51	18.7	68.5	1	1	1	0	0	1	109	70	62	7.4	7.4	7.4	83	13	0.75	240	37	73	175	5.1	1
1	60	26.8	94.5	2	2	2	0	0	0	132	89	60	7.9	8.2	8.05	114	14	0.87	205	112	59	134	5.9	1
1	69	22.0	77	1	1	2	0	0	0	123	84	85	11.2	9.3	10.25	87	18	1.07	157	55	55	105	5.6	1
1	56	27.1	92.6	1	1	1	1	0	1	122	85	66	6.3	6.8	6.55	114	19	1.07	199	165	63	129	6.4	1
2	54	21.2	78	1	1	1	0	0	0	124	85	55	6.6	6.5	6.55	87	12	0.57	189	32	45	143	5.8	0
2	54	30.5	94	1	1	1	1	1	1	161	88	68	7.2	7.3	7.25	119	12	0.62	163	134	53	97	7	0
2	69	24.0	83	1	1	2	1	0	1	135	84	86	9.5	9.3	9.4	88	14	0.63	174	36	77	101	6.1	1
1	57	23.5	83	2	2	1	0	0	0	130	84	66	7.1	6.7	6.9	99	14	0.98	202	73	47	153	6.4	1

2	58	20.8	80.5	1	1	1	0	0	1	133	84	69	8.9	8.4	8.65	88	10	0.55	164	59	68	96	5.8	1
1	52	23.1	83	2	2	1	1	1	1	133	83	56	8.6	8.1	8.35	213	14	1.05	176	276	46	89	7.6	0
1	53	24.5	86	1	2	1	0	0	1	109	80	54	7.8	7.7	7.75	112	20	0.96	243	151	46	177	5.5	0
2	57	21.6	77	1	1	1	0	0	0	93	56	61	7.4	7.5	7.45	105	15	0.79	195	43	66	113	6	1
1	49	24.0	94.5	2	1	1	1	0	0	167	115	83	9.4	9.4	9.4	102	10	0.84	220	154	61	148	5.8	0
1	51	29.0	93	2	2	1	1	0	1	116	73	62	6.3	6.5	6.4	100	19	0.99	172	148	46	115	5.6	1
1	45	22.8	80	1	1	2	0	0	0	126	85	63	6.8	6.9	6.85	95	15	0.88	144	58	74	74	5.5	0
1	53	29.3	98.5	2	1	2	1	0	0	129	88	58	8.2	9.4	8.8	100	9	0.99	184	135	53	115	5.6	1
2	44	29.1	97	1	1	1	1	0	1	162	105	73	7.5	7.2	7.35	103	6	0.78	238	146	54	166	5.6	0
1	66	26.4	92	1	1	1	1	1	1	146	91	77	6.9	6.9	6.9	129	13	0.84	155	177	60	81	6.5	0
1	51	21.9	76	1	1	1	0	1	0	116	73	61	7.1	7.2	7.15	128	16	1	162	52	61	87	6	0
1	56	24.9	83.5	1	1	1	0	0	0	138	89	64	7.6	7.3	7.45	105	14	0.91	205	72	67	133	5.5	1
1	40	25.7	91	2	1	2	0	0	0	123	74	51	6.4	6.5	6.45	87	9	1.07	219	94	58	145	5.2	1
2	56	25.1	90	1	1	1	0	0	0	122	74	54	7	6.8	6.9	99	20	0.68	226	31	92	142	5.6	1
1	61	20.2	75	2	2	1	0	0	0	132	89	59	13.5	13.6	13.55	98	20	0.9	187	32	84	115	5.5	1
1	59	24.4	87	2	2	2	1	0	1	134	90	56	9	8.5	8.75	94	11	0.94	221	76	60	137	5.6	0
2	55	19.2	67	1	1	1	0	0	1	116	77	66	8.2	8.3	8.25	111	19	0.66	198	103	69	112	6.2	1
2	56	24.8	82	1	1	1	0	0	0	104	68	61	6.9	6.7	6.8	104	13	0.71	220	153	48	155	5.9	0
2	52	20.7	70	1	1	1	0	0	0	125	77	59	8.5	8.6	8.55	92	16	0.81	167	32	87	90	4.9	0
1	42	25.1	92	2	1	2	0	1	1	116	72	69	7.5	7.3	7.4	171	22	0.91	218	449	46	123	7.2	1
1	51	22.3	87	2	1	1	0	0	1	123	77	60	9.1	9.1	9.1	100	19	0.81	227	148	49	163	5.9	0
2	34	20.1	70.7	1	1	1	0	0	0	110	66	55	5.9	5.7	5.8	83	13	0.75	208	59	81	120	5.2	0
1	32	24.5	80.8	1	1	1	0	0	1	124	74	79	6.6	6.7	6.65	79	11	1	339	47	59	274	5.6	0
1	37	33.6	111	1	2	1	0	0	1	134	89	67	7.6	7.7	7.65	99	14	0.92	216	97	52	165	5.9	0
1	50	26.6	94.5	1	1	1	0	1	0	115	73	57	8.2	7.8	8	159	13	0.86	121	41	40	78	6.7	0
1	53	25.3	87	2	1	1	0	0	0	133	88	83	10.7	7	8.85	108	9	0.93	175	135	54	112	5.7	0
1	60	34.5	115.3	1	1	1	0	0	1	122	76	56	6.5	6.3	6.4	103	10	1.09	219	281	39	133	6	1
1	69	23.9	86.5	1	1	1	0	1	0	137	83	57	7.5	7.7	7.6	143	17	1	178	171	42	112	6.3	0
1	63	24.6	85	2	1	2	0	1	0	126	77	63	8.4	8.1	8.25	135	19	0.9	165	39	56	115	6.9	1

1	49	25.9	95	2	2	1	0	0	1	118	81	78	8.9	8.4	8.65	98	14	1.12	174	243	42	108	5.4	1
2	57	20.2	74	2	2	1	1	0	1	134	92	72	9.9	8.1	9	95	13	0.65	173	62	84	88	5.5	0
2	28	18.6	73	1	1	1	0	0	0	101	66	70	6	6.1	6.05	74	8	0.73	210	45	104	111	5.3	1
1	27	22.1	90	1	1	1	0	0	0	124	83	62	6.3	6.8	6.55	72	9	0.82	192	71	58	127	5.3	1
1	74	25.0	92	1	1	2	1	0	1	150	93	68	10.5	8.5	9.5	115	14	0.83	168	86	70	96	5.7	0
1	76	24.7	93.5	1	2	1	1	0	0	121	89	73	9.6	9	9.3	121	17	0.87	186	58	77	111	5.6	0
1	42	24.5	77	1	1	2	1	0	0	160	109	68	6.7	7.1	6.9	91	10	0.73	176	53	59	112	5.2	0
1	49	24.8	92	1	2	1	1	0	1	132	97	70	7.2	7	7.1	106	8	0.75	190	240	38	128	5.5	0
1	50	26.5	99	1	1	2	0	0	0	118	83	53	6.9	7.2	7.05	89	11	0.74	171	55	45	118	5.5	0
2	50	17.1	65	1	1	1	1	0	0	131	93	70	8.9	8.3	8.6	98	9	0.59	170	46	80	91	5.4	1
1	68	22.5	79	2	1	1	1	1	1	138	98	60	8.7	8	8.35	117	20	0.93	164	149	41	106	8.6	1
1	45	23.8	89	1	1	1	0	0	1	128	83	76	6.5	6.4	6.45	85	16	0.83	206	184	54	131	5.8	0
2	40	23.1	74	1	1	1	0	0	1	124	73	62	6.8	6.9	6.85	78	8	0.71	239	74	58	183	5.4	0
1	57	26.9	90	2	1	2	0	0	0	132	82	50	7.5	7.7	7.6	91	22	0.89	154	119	45	93	6.2	0
1	41	20.2	77	2	1	2	0	0	1	109	69	52	7.3	7.2	7.25	88	16	1.06	280	112	55	213	5.4	0
2	55	19.8	81.5	2	2	1	0	0	0	106	69	47	7.8	7.6	7.7	96	14	0.68	235	58	86	159	5.4	1
1	63	23.1	90.5	2	1	1	1	1	1	135	91	75	9.7	9.9	9.8	131	8	0.65	125	59	48	76	6.7	1
1	55	26.5	92	1	2	2	1	0	1	146	103	58	7.9	7.8	7.85	92	15	1.27	251	240	52	177	5.6	1
1	64	24.2	89	2	2	1	1	1	0	131	89	65	10.5	9.4	9.95	360	17	0.93	195	172	65	120	11	0
1	66	24.8	83	1	1	1	1	0	0	152	99	58	6.5	7	6.75	107	12	0.92	187	127	57	119	6.3	1
2	58	20.8	77	1	1	1	0	0	0	117	84	68	8.2	7.6	7.9	87	10	0.57	215	36	97	124	5.5	1
1	65	24.3	89	1	2	2	0	1	0	117	79	61	7.9	8.1	8	141	7	0.78	193	51	64	133	6.5	0
1	50	25.8	93	1	2	1	1	0	0	146	92	91	8.8	8.6	8.7	101	9	0.84	195	187	40	128	5.4	0
1	57	26.1	98.5	1	1	1	1	0	0	141	90	58	10.6	8.2	9.4	95	14	1.02	236	164	57	154	6	0
1	51	25.0	90	2	1	1	0	0	1	108	81	66	7.3	7.2	7.25	91	17	0.97	261	140	49	193	5.6	1
1	43	29.4	107	1	1	1	1	0	0	159	108	103	8.7	8.2	8.45	100	20	1.02	215	133	45	156	5.9	1
1	53	24.6	84	1	1	2	0	0	0	127	81	63	6.9	6.9	6.9	102	13	0.94	217	142	45	158	5.4	0
2	55	33.2	111	1	1	1	0	0	0	129	80	72	5.7	6.1	5.9	95	12	0.6	198	139	50	128	5.8	1
1	60	26.3	94	2	1	1	1	1	1	136	95	88	9.6	9.4	9.5	189	16	0.87	228	324	41	155	7.5	1

1	76	26.0	96	1	1	1	1	1	1	132	74	56	8.8	8.4	8.6	180	15	0.85	123	176	31	74	8.7	0
2	71	22.8	80	1	1	1	1	0	1	152	87	66	8	12.3	10.15	122	19	0.6	173	148	51	100	5.8	1
2	56	30.0	103	1	1	1	1	0	0	141	86	67	8.7	9	8.85	93	8	0.72	166	68	55	105	6.1	0
1	63	29.1	98	2	2	1	1	1	0	141	97	73	8.9	8.3	8.6	128	15	1.01	204	170	47	142	6	1
1	58	23.4	82	2	2	1	0	0	0	115	87	73	9.4	8.1	8.75	100	15	0.88	219	128	44	154	5.6	1
2	68	24.6	85	1	1	1	0	0	1	129	75	66	8.6	8.8	8.7	96	11	0.56	110	94	33	72	5.6	1
1	56	28.0	94	2	2	1	1	0	1	143	89	100	8.2	7.6	7.9	88	14	0.83	144	202	31	90	5.6	1
1	58	24.0	90	1	1	2	0	0	1	131	88	58	7.6	7.7	7.65	118	18	0.82	221	222	59	124	6	0
1	68	30.1	108	1	2	1	0	0	0	137	83	49	8	7.9	7.95	110	10	0.79	195	78	59	131	6.1	1
2	65	23.6	89	1	1	1	0	1	1	124	75	68	9.3	9	9.15	174	7	0.61	159	139	42	100	10.3	1
1	66	26.4	97	2	2	2	0	0	0	133	89	54	8.7	8.7	8.7	98	18	1.03	203	122	47	147	5.5	1
1	58	28.1	96	2	2	1	1	0	0	152	96	48	7.8	8.1	7.95	102	10	0.81	217	154	46	155	5.7	0
2	56	23.5	89	1	1	1	0	0	0	108	78	59	6.8	7.2	7	110	17	0.56	218	108	66	136	5.8	0
1	46	24.5	88	1	2	1	0	0	0	116	78	71	6.3	6.5	6.4	104	14	1.16	163	127	52	99	5.3	1
2	59	21.4	81	1	1	1	0	1	0	124	77	56	7.5	7.2	7.35	106	11	0.62	169	76	67	99	6.5	0
1	65	25.6	94	1	1	1	1	0	1	144	96	74	8.4	8.7	8.55	93	14	0.88	175	123	37	124	5.5	0
1	57	20.5	79.5	1	1	1	1	0	1	134	93	81	8.2	8.4	8.3	90	18	1.09	250	70	67	180	5.5	1
1	33	30.5	100	2	1	1	0	0	1	136	89	85	6.9	6.6	6.75	85	14	1.04	248	159	40	190	5.8	1
1	53	27.9	97	1	1	2	0	0	0	125	87	69	6.3	6.3	6.3	96	13	0.93	199	44	53	148	5.6	1
2	44	24.3	83	1	1	1	0	0	1	117	79	61	7.1	7.1	7.1	90	9	0.58	226	83	46	172	5.8	1
1	49	26.5	91.5	1	2	1	1	1	1	142	105	78	8.7	7.4	8.05	190	17	0.66	243	177	49	169	8.9	1
2	55	24.4	84	1	1	1	0	0	0	121	78	57	7.8	8.1	7.95	101	14	0.62	191	127	53	122	5.2	1
1	48	23.5	90	2	1	1	1	0	1	135	97	75	7.6	7.9	7.75	103	11	0.93	245	116	42	185	5.9	1
2	67	21.9	76	1	1	1	0	0	0	118	72	60	7.9	7.6	7.75	107	7	0.61	225	75	80	137	6.1	0
1	44	23.0	76.5	1	2	2	0	0	0	115	65	59	7.9	7.9	7.9	89	12	0.99	194	91	58	133	5.3	0
2	51	21.7	75	1	1	1	0	0	0	126	82	66	7.9	7.7	7.8	89	14	0.59	145	34	53	92	5.9	0
1	45	22.8	84	1	2	1	0	0	0	117	79	69	7.7	7.7	7.7	103	16	0.95	206	107	54	157	6	1
2	46	22.6	84	1	1	1	0	0	0	109	71	64	6.8	6.8	6.8	98	9	0.63	178	75	61	109	5.3	0
2	41	23.0	90	1	1	1	0	0	0	111	77	65	7.5	7.4	7.45	98	12	0.91	208	67	81	115	5.6	1

1	58	23.3	85	2	1	1	0	0	1	133	89	76	8.7	8.9	8.8	112	17	0.91	249	148	49	178	5.8	1
2	54	21.2	76	1	1	2	1	0	0	136	96	100	8.7	8.6	8.65	102	13	0.62	200	116	57	128	4.8	0
1	62	20.5	71	1	1	1	0	0	0	126	88	57	6.9	7	6.95	90	13	0.92	154	41	73	83	5.3	1
1	59	25.0	94	2	1	1	1	1	1	122	84	72	8.7	8.1	8.4	163	11	0.74	234	277	34	150	7.6	1
1	35	29.9	104	2	2	1	1	0	0	145	104	58	7.4	7.1	7.25	107	13	0.76	181	151	45	114	6.1	1
1	53	21.1	80	1	1	1	1	1	1	124	90	63	7.4	6.9	7.15	199	12	0.71	147	161	34	92	7.5	1
1	47	28.7	100	1	1	1	1	1	1	145	106	60	7.3	7	7.15	126	10	0.63	133	266	37	66	6.5	1
2	58	25.5	86	1	1	1	0	1	1	132	79	69	9.5	9.3	9.4	159	16	0.66	185	98	48	123	7.9	1
2	62	22.8	78.5	1	1	1	1	0	1	158	101	69	8.4	8.1	8.25	120	18	0.62	238	104	59	157	5.5	0
1	43	24.0	91	1	2	1	0	0	0	116	82	62	7.9	8.1	8	93	12	0.81	210	73	43	154	5.4	0
2	62	26.7	86	2	1	1	0	1	1	127	70	52	7.9	7.7	7.8	110	17	0.89	174	80	48	118	6.6	0
2	52	19.3	73	1	1	1	0	0	0	101	70	62	7.6	7.7	7.65	80	17	0.62	146	48	58	87	5.4	1
1	55	26.7	93	1	1	1	0	0	1	128	82	66	7.1	7	7.05	86	12	0.88	202	93	38	147	5.1	1
1	50	25.7	84	1	1	2	0	0	0	126	85	58	7.3	7.1	7.2	96	14	1	197	57	60	129	5.8	0
1	59	28.9	102	1	1	1	1	0	1	127	90	70	7.1	7.3	7.2	109	15	0.8	143	64	33	101	6	1
2	56	22.9	81	1	1	1	1	0	1	140	98	59	9	8.4	8.7	85	10	0.7	211	204	49	123	5.8	0
1	34	24.4	98	2	2	1	1	1	1	162	107	63	7.2	6.5	6.85	136	16	0.83	231	240	41	152	6.5	1
1	46	21.8	86	1	2	1	1	0	0	136	93	69	7.5	7.3	7.4	125	14	0.84	169	122	53	99	5.3	0
1	39	27.6	97	1	1	2	0	0	1	130	77	64	7.1	7.1	7.1	107	18	1.29	241	66	48	183	5.5	1
1	33	24.9	87	1	1	1	0	0	1	134	89	60	7.8	8.3	8.05	98	10	1.04	187	237	33	125	5.5	0
2	51	23.4	79.2	1	1	1	1	0	0	131	89	66	7.3	7.2	7.25	94	17	0.68	183	39	82	106	5.5	1
2	54	23.6	81	1	1	1	0	0	1	118	87	68	7.6	8.5	8.05	88	14	0.65	228	54	59	166	5.5	1
1	48	28.7	101.3	1	1	1	0	1	0	137	88	55	6.6	6.9	6.75	151	11	0.77	151	83	54	91	6.8	0
1	38	29.6	104	1	2	1	0	0	0	125	82	73	7.2	7.4	7.3	108	17	0.96	207	182	40	137	5.6	0
1	40	22.3	85.5	2	2	1	1	0	1	133	92	67	8.4	8.3	8.35	102	14	0.93	172	263	27	116	5.6	1
1	53	25.6	89	1	1	2	0	0	0	124	85	56	5.9	6.1	6	81	14	0.88	194	52	67	126	5.4	0
2	49	21.0	72	1	1	1	0	0	1	123	78	69	7.7	7.7	7.7	97	10	0.68	321	111	80	198	5.6	0
1	51	22.1	90	2	1	1	1	0	0	138	96	81	9.2	9	9.1	105	13	0.89	157	133	40	103	5.7	0
2	49	39.9	126	1	1	1	1	0	0	160	90	93	5.9	4.2	5.05	121	6	0.52	176	60	59	115	5.6	0

2	49	21.3	71	2	1	1	0	0	0	114	70	63	7.6	7.4	7.5	100	10	0.75	126	48	50	72	5.6	1
1	60	22.5	88	2	1	1	0	0	1	136	87	63	8.8	8.8	8.8	100	16	0.78	158	109	36	104	5.8	1
2	50	21.5	73	1	1	1	0	0	1	128	89	77	6.2	6.1	6.15	87	11	0.89	235	76	64	164	5.1	1
1	54	22.2	88	1	1	1	0	0	1	114	78	82	7.4	7.4	7.4	104	13	0.72	153	49	36	114	5.6	1
1	30	32.8	113.5	2	2	1	1	0	1	139	97	79	6.9	7.1	7	88	11	0.72	242	363	40	145	5.6	1
2	53	25.2	89	1	1	1	0	0	0	121	77	64	6.9	6.8	6.85	95	8	0.62	186	47	70	113	5.4	0
2	51	26.9	89	1	1	1	0	0	0	111	78	72	6.1	6	6.05	84	14	0.8	166	47	58	104	5.6	0
2	56	25.5	90	1	1	1	1	0	1	130	97	66	8.7	8.6	8.65	105	17	0.71	238	54	72	161	5.7	1
2	51	22.8	83	1	1	1	0	0	0	122	83	66	6.8	7	6.9	71	13	0.72	185	47	59	127	5.3	1
2	54	20.7	75.5	1	1	1	0	0	0	118	65	64	8.1	7.9	8	92	10	0.67	232	67	71	147	5.4	1
2	49	23.9	81	1	1	1	1	0	1	141	74	71	8.3	8.5	8.4	83	11	0.6	220	64	80	130	5.4	0
1	58	26.7	94	2	1	1	0	0	1	117	83	70	7.9	8.1	8	97	14	0.94	250	91	56	180	6.1	1
2	56	20.0	70	1	2	1	0	0	1	114	77	68	7.2	7.3	7.25	106	16	0.72	289	66	110	168	5.8	0
2	55	22.7	78	1	1	2	1	0	0	141	92	55	8.1	8.1	8.1	95	11	0.66	211	160	46	148	5.5	0
2	55	17.3	64	1	1	2	1	0	0	145	105	69	9.1	8.8	8.95	85	12	0.79	209	71	82	118	5.6	1
1	58	26.7	97	2	2	2	1	0	0	132	94	66	8.9	8.6	8.75	95	16	0.94	230	75	64	148	5.7	0
1	61	28.0	93	2	1	1	1	1	1	161	104	53	8	8.1	8.05	125	14	0.87	197	72	49	140	7.3	1
2	53	27.3	89	1	1	1	0	0	1	124	82	58	7.1	6.8	6.95	96	17	0.62	247	85	65	169	5.7	1
1	49	22.5	74.8	1	2	1	1	0	0	130	97	86	6.3	6.3	6.3	94	10	0.97	226	49	67	153	5.7	1
2	53	24.9	89	1	1	1	1	0	0	141	95	65	6.5	6.6	6.55	88	18	0.57	225	46	95	129	5.6	0
1	53	21.5	76	2	2	1	0	0	0	110	81	50	8	8	8	103	12	0.84	166	99	81	77	5.4	0
2	50	24.4	88	1	1	1	0	0	0	131	83	67	6.2	6.5	6.35	95	8	0.64	177	60	56	109	5.7	0
2	55	26.1	88	1	1	1	0	0	1	136	82	57	7.4	7.3	7.35	94	11	0.7	260	79	59	191	6.1	1
2	76	27.9	96	1	1	1	0	0	1	138	83	86	10.2	10.5	10.35	104	18	0.63	198	170	58	119	5.9	1
2	64	24.8	86	1	1	1	0	0	0	132	86	79	7.3	7.6	7.45	112	17	0.64	205	111	53	140	5.6	1
1	51	29.6	104	1	2	1	1	0	1	139	87	68	7.2	6.6	6.9	109	14	0.64	137	111	50	79	5.7	0
2	43	21.8	82	1	1	1	0	0	0	102	72	73	7.5	7.7	7.6	92	10	0.58	198	92	51	136	5.7	0
2	63	26.0	89	1	1	1	0	0	1	113	68	73	7.1	7.2	7.15	112	12	0.6	239	95	55	169	6	0
1	66	25.9	95	2	2	2	1	1	0	135	90	55	7.8	8	7.9	111	13	0.73	181	81	68	110	5.8	0

2	68	23.6	82	1	1	1	1	0	0	139	88	91	10	9.3	9.65	100	9	0.61	191	45	70	115	5.6	1
2	74	26.7	95	1	1	1	1	0	1	135	86	90	9.1	8.4	8.75	89	14	0.57	190	126	69	105	5.6	1
1	52	21.2	78	1	1	1	0	0	0	110	78	65	7.5	7.5	7.5	123	12	0.95	159	74	62	100	6.3	0
2	64	21.9	74	1	1	1	0	0	1	122	86	55	6.4	6.5	6.45	89	12	0.73	249	52	105	146	5.9	0
1	64	24.5	83.5	1	1	1	0	0	0	131	89	58	7.9	8.2	8.05	98	11	0.85	170	39	65	104	5.4	1
2	48	22.5	81	1	1	1	0	0	0	124	79	68	7.4	7.5	7.45	94	10	0.66	229	103	88	135	5.4	0
1	55	25.4	94	2	1	1	1	0	0	133	96	66	7	7.4	7.2	109	16	1.01	197	91	66	129	5.5	0
1	48	31.5	114	1	2	1	1	0	1	147	94	53	7.1	7.6	7.35	96	10	0.93	237	340	49	83	5.6	0
2	52	21.2	74	1	1	1	0	0	0	103	54	62	7.8	8	7.9	76	19	0.78	208	37	83	124	5.3	1
1	46	32.1	113	2	1	1	1	0	0	156	108	54	6.7	6.8	6.75	102	12	0.84	206	127	43	151	5.7	1
1	68	25.9	95	1	1	1	1	1	1	137	88	56	9.1	8.9	9	129	9	0.81	206	64	50	151	6.4	1
1	54	26.1	89	2	1	2	1	0	0	130	94	72	8.9	8.5	8.7	100	15	0.96	173	80	49	119	5.6	1
1	48	26.1	85	1	2	1	0	0	1	128	86	66	6.5	6.4	6.45	90	12	0.8	241	52	81	166	5.4	0
1	58	27.7	99	1	1	1	0	0	1	115	77	58	7.2	7.2	7.2	101	12	0.83	220	200	34	158	5.6	1
1	51	26.6	94	1	1	1	1	0	0	144	98	76	9.3	8.1	8.7	118	12	0.7	155	52	60	90	4.9	1
1	47	25.3	87	2	2	1	1	0	0	153	96	75	8.6	8.2	8.4	108	12	0.65	175	136	74	95	4.9	1
1	41	25.1	90	1	1	1	0	0	1	125	86	52	7.3	7.1	7.2	97	16	1	193	374	33	103	5.6	1
1	49	25.1	91	2	1	1	1	0	0	124	94	71	8.3	8.1	8.2	103	11	1.01	187	87	56	125	5.6	1
1	57	23.2	89	1	1	2	1	0	0	146	88	64	6.6	6.8	6.7	93	14	0.85	200	147	53	134	5.4	0
2	60	19.8	77	1	1	1	0	0	1	131	74	85	9.2	9.1	9.15	100	14	0.63	232	104	93	127	5.6	1
1	56	26.5	96	1	1	1	1	0	0	125	86	62	7.2	7.2	7.2	108	14	0.87	122	63	52	69	5.5	1
1	64	22.8	83	2	2	1	1	1	1	114	85	76	8.5	8.5	8.5	120	11	0.78	112	76	53	59	6.5	0
1	51	26.1	95	1	1	2	1	0	1	107	75	63	6.5	6.3	6.4	103	16	0.98	182	193	30	123	5.4	1
1	51	25.4	89	2	1	1	1	1	0	145	108	56	8.1	7.5	7.8	180	18	1.01	213	103	53	151	7.5	1
1	68	22.9	91	2	1	1	0	1	1	121	77	69	10.1	10.1	10.1	225	15	0.88	139	140	34	94	7.7	0
1	46	19.3	77	2	1	1	0	0	0	106	78	55	9.1	9.3	9.2	89	17	1.05	197	103	63	124	5.4	0
1	59	27.4	98.5	2	1	1	1	0	0	155	93	71	7.6	7.7	7.65	108	23	0.9	204	105	44	146	5.7	0
2	51	24.0	83	1	1	1	1	1	1	125	97	78	7.1	7.1	7.1	139	12	0.4	261	124	65	178	7.2	1
1	59	25.5	92	2	1	1	1	1	0	125	89	68	8.2	7.5	7.85	120	18	0.84	160	103	50	92	6.6	1

2	58	23.1	81.5	1	1	1	0	0	0	106	72	56	6.7	6.6	6.65	106	17	0.75	202	110	52	134	6.1	1
1	45	24.7	95	2	1	1	0	0	1	108	72	82	7.3	7.3	7.3	93	16	1.09	219	215	34	150	5.6	0
2	41	22.4	76	1	1	1	0	0	0	123	83	60	7.4	7.4	7.4	86	11	0.7	231	70	97	127	5.5	1
1	56	24.6	94	2	1	2	0	0	0	124	89	53	7.4	7.5	7.45	106	20	0.81	156	186	44	93	5.7	0
2	65	23.4	85	1	1	2	0	0	0	104	68	72	7.7	7.7	7.7	75	11	0.75	199	44	41	156	5.8	0
1	60	21.6	82	1	1	1	0	0	0	108	78	77	9.3	9.3	9.3	105	13	0.81	206	70	88	116	5.4	1
1	35	24.8	91	2	1	2	0	0	0	138	81	66	4.5	4.7	4.6	89	15	1.09	197	53	94	104	5.4	0
1	59	25.2	90.5	2	2	1	0	1	1	128	88	63	8.3	8.2	8.25	143	13	0.97	260	166	40	194	6.8	1
2	56	23.7	80	1	1	1	0	0	0	114	71	63	8.2	8.4	8.3	103	11	0.62	209	122	57	134	5.6	1
1	56	22.4	92	1	1	1	1	0	0	139	91	61	8.7	8.6	8.65	108	20	0.89	170	110	41	111	5.9	0
2	50	35.5	105	1	1	1	0	0	0	116	80	71	5.7	5.7	5.7	76	8	0.64	187	58	60	118	5.5	1
1	58	21.5	79	2	1	2	0	0	0	127	84	65	8.3	8.2	8.25	119	19	0.94	178	60	71	104	6.2	1
1	45	24.9	92	2	2	1	0	0	1	130	85	47	7.8	7.3	7.55	97	14	1.01	223	221	49	142	5.5	0
1	44	23.6	85	2	1	1	0	0	0	118	81	60	8.4	8.2	8.3	88	14	0.91	213	166	46	140	5.4	0
1	60	26.3	87.8	2	1	1	1	0	0	141	95	65	8.3	7.5	7.9	94	16	0.81	157	188	45	83	5.9	0
2	66	21.8	78	1	1	1	1	1	1	148	98	102	9.8	9.8	9.8	194	15	0.69	214	92	43	168	8.2	0
1	52	31.6	100	1	1	1	1	1	1	128	76	84	5.5	5.9	5.7	131	10	0.89	107	55	37	66	7	1
2	49	20.2	78	1	1	1	0	0	1	110	77	61	7.2	7.5	7.35	89	15	0.84	242	62	73	159	5.4	1
1	52	23.9	88	1	1	2	0	0	0	111	69	52	7	7.3	7.15	100	13	0.89	209	61	60	142	5.7	0
1	63	28.4	99	2	1	1	1	1	1	116	75	49	4.8	4.8	4.8	115	19	1.05	177	236	27	118	6.6	1
1	47	22.3	79	2	1	1	0	0	0	117	75	66	7.5	7.6	7.55	99	12	0.8	227	163	40	150	5.4	1
2	59	29.7	100	1	1	1	0	1	0	116	79	59	7.5	7.5	7.5	238	12	0.7	165	111	51	99	10.2	0
1	59	23.7	89	2	1	1	1	0	0	146	88	52	9.4	10.9	10.15	69	16	1.11	139	41	51	83	5.4	1
2	81	19.7	82	1	1	2	1	0	1	165	78	62	10.7	10.3	10.5	100	13	0.47	171	64	80	88	6.4	0
1	60	25.8	96	1	2	1	1	1	0	130	91	61	8.1	7.5	7.8	133	19	1.03	203	148	41	144	6.2	1
1	52	24.1	89	2	1	1	0	0	1	122	82	61	8	8.4	8.2	86	9	0.86	188	165	38	132	5.4	0
1	51	23.9	88	1	2	2	0	0	0	119	82	62	6.4	6.7	6.55	106	13	0.8	205	72	65	119	5.7	0
1	51	26.7	97	2	1	1	0	1	1	121	84	55	7.8	7.5	7.65	161	12	0.94	244	305	31	164	8.6	1
1	68	22.5	81	2	2	1	1	1	0	128	92	59	8.2	8.1	8.15	136	13	0.7	193	49	61	123	6.4	1

1	50	22.2	90	1	1	1	0	0	0	121	77	58	7.5	7.4	7.45	104	9	1.04	192	62	70	122	5.4	0
2	43	19.7	71	1	1	1	0	0	0	116	82	72	7.2	7.3	7.25	90	10	0.65	187	39	77	108	5.4	0
1	50	27.0	93	2	2	1	1	1	0	139	100	64	7.9	7.6	7.75	122	10	0.85	201	150	50	132	6.5	1
2	57	23.6	80	1	1	2	0	0	1	124	78	50	8.4	8.6	8.5	106	17	0.81	261	89	66	181	5.7	0
1	51	25.2	92	2	1	1	0	0	0	110	71	44	8	7.8	7.9	104	13	0.89	180	179	47	108	5.8	1
1	55	25.0	91	1	1	1	1	0	1	138	87	68	7.7	7.6	7.65	98	17	0.88	200	117	51	133	5.8	0
1	93	20.2	79	2	1	1	0	1	1	117	69	77	13.2	12.5	12.85	344	16	1.22	180	229	40	105	16.4	1
1	56	26.0	92	1	1	1	1	0	0	115	87	68	7.8	7.8	7.8	86	10	0.88	175	94	45	118	5.5	0
2	56	25.6	88	1	1	1	0	0	0	119	73	58	7.4	7.5	7.45	95	12	0.53	210	105	48	157	6	0
1	46	26.8	90	2	2	1	1	1	1	155	104	86	10.7	10.4	10.55	237	14	0.56	202	370	68	106	8.7	1
1	49	22.7	86	1	2	1	1	0	0	122	90	72	7.9	7.6	7.75	100	10	1.02	172	150	42	103	5.4	0
2	43	20.7	78	1	1	1	0	0	0	111	70	78	7.1	7	7.05	88	11	0.66	211	116	60	125	5.6	0
1	52	22.7	87	1	1	1	1	1	1	123	78	71	7.4	7.7	7.55	180	13	1.16	162	126	35	106	8.1	0
1	60	21.0	80	2	2	1	1	1	0	137	87	56	8.8	8.6	8.7	136	13	0.83	204	154	63	115	5.4	0
2	57	21.0	78.5	1	1	2	0	0	1	134	81	63	7.9	7.5	7.7	104	9	0.6	245	166	52	166	5.6	1
1	45	39.2	130	1	1	1	1	1	1	157	106	65	5.7	5.6	5.65	139	14	0.87	211	286	35	140	7.4	0
1	56	23.8	85	1	1	2	1	0	0	128	92	60	7.1	7.2	7.15	98	11	0.92	164	61	50	110	5.6	0
2	59	24.8	92	1	1	1	0	0	0	114	74	74	7.3	7.6	7.45	100	8	0.61	191	71	57	124	5.8	1
1	58	34.9	112.5	2	1	1	1	1	1	149	103	71	8.9	8.8	8.85	80	20	1.37	121	96	52	61	6.1	0
1	35	22.5	78	1	1	1	0	0	1	124	76	56	7.2	7.1	7.15	95	15	1.09	248	164	62	158	5.4	1
2	63	24.0	78	1	1	1	0	0	1	133	72	61	7.6	7.6	7.6	102	11	0.65	176	45	57	110	5.4	1
1	39	22.8	85.5	2	2	1	0	0	1	117	76	61	8	7.9	7.95	107	8	0.77	189	422	38	105	6	1
2	39	21.6	79	1	1	1	0	0	0	128	71	41	6.9	6.9	6.9	84	11	0.64	153	47	71	80	5.1	0
1	42	24.5	92	2	1	2	0	0	1	117	72	52	7.1	7.1	7.1	124	12	1.13	186	156	39	121	5.5	1
1	61	22.7	84	2	1	1	0	0	0	106	73	76	9.1	8.7	8.9	95	14	0.93	194	110	41	137	6.3	0
1	53	25.1	95	2	2	2	1	1	1	130	91	56	7.4	7.4	7.4	127	15	1.01	166	301	34	88	5.5	1
1	55	23.5	84	1	2	1	1	0	0	136	83	65	6.7	6.6	6.65	89	13	0.78	162	57	48	110	6	0
2	63	28.2	91	1	1	1	1	0	1	147	78	63	8.4	8.7	8.55	92	10	0.69	290	70	71	210	5.9	1
1	56	24.0	88	1	1	1	0	0	0	126	87	64	7.8	7.9	7.85	106	16	0.99	193	86	61	126	5.6	0

2	55	44.0	134	2	1	1	1	0	1	160	110	59	6.8	6.6	6.7	101	13	0.63	199	150	39	141	5.7	0
1	56	23.5	86.5	2	1	1	0	0	1	125	83	58	8.3	8.3	8.3	115	16	0.71	277	157	55	198	6.4	1
2	66	24.9	87	1	1	1	0	0	1	135	85	65	8.4	8.4	8.4	95	23	1	238	74	62	169	5.6	1
2	53	25.5	88	1	1	1	0	0	0	121	71	56	6.3	6.3	6.3	97	15	0.74	224	122	48	154	5.6	1
2	51	19.0	70	1	1	1	0	0	0	123	72	60	7.2	7.3	7.25	96	17	0.74	183	55	88	97	5.4	0
1	55	22.8	84	2	2	1	0	0	0	119	88	61	8.6	8.1	8.35	103	14	0.88	197	79	53	131	5.7	1
2	54	23.4	85	1	1	1	1	0	1	102	64	50	7.1	7.2	7.15	102	14	0.61	135	67	72	66	5.7	0
2	65	22.8	87	1	1	1	1	0	1	156	95	64	9.7	10.1	9.9	105	17	0.69	174	126	75	84	6	1
1	60	26.4	93	2	1	1	1	1	1	128	82	73	7.9	8.3	8.1	179	18	1.15	110	155	39	54	7.5	1
1	59	24.0	88	1	1	1	1	0	1	124	84	65	9.5	9	9.25	93	16	1.05	166	47	58	109	5.5	1
1	49	28.1	96	1	1	2	0	0	0	127	80	54	6.4	6.7	6.55	92	16	1.1	153	119	41	100	5.5	0
1	54	32.2	106	1	1	1	1	0	1	128	87	72	6.4	6.4	6.4	110	17	1.09	147	133	38	94	6.2	0
2	67	20.2	84	1	1	1	0	0	0	137	71	50	6.9	6.9	6.9	92	11	0.65	226	53	72	146	5.7	0
2	58	22.3	78	1	1	1	1	1	1	142	85	73	8.2	8.1	8.15	130	11	0.62	177	66	53	118	6.2	1
1	63	24.3	87	1	1	1	1	1	1	135	93	59	10.2	10	10.1	206	17	1.1	202	462	31	92	9.5	1
2	43	17.0	57.5	1	1	1	0	0	0	125	68	62	7.5	8.1	7.8	107	12	0.67	187	64	58	125	5.4	1
1	53	25.1	89	2	1	1	0	0	0	113	80	60	7.4	7.4	7.4	86	13	1.24	202	55	41	156	5.9	1
2	51	24.5	85	1	1	1	0	0	0	104	69	68	6	6.1	6.05	94	6	0.65	239	113	66	147	5.4	1
1	49	25.9	95	2	2	1	1	0	1	119	82	71	7.1	6.9	7	104	11	0.87	214	299	34	130	5.5	0
1	55	27.2	89.5	1	1	2	1	0	1	133	94	64	9.6	13.5	11.55	106	14	0.91	163	149	44	99	5.5	0
1	52	20.7	74	2	1	1	0	0	0	88	60	54	7.2	7.1	7.15	91	13	0.84	162	77	50	102	5.4	1
1	46	25.2	90.5	1	1	1	0	0	0	122	85	55	6.8	6.9	6.85	96	13	0.86	201	68	82	118	5.8	1
1	52	23.4	89	1	2	1	1	1	1	143	103	50	9.4	9.2	9.3	179	11	0.78	233	162	42	172	7	1
2	53	24.0	82.5	1	1	1	0	0	1	102	74	83	7.4	7.3	7.35	100	16	0.7	271	131	55	189	5.6	0
2	55	16.0	61	1	1	1	0	0	0	132	83	73	7.8	8	7.9	101	13	0.72	160	95	54	88	5.6	1
1	73	28.5	102	1	1	1	1	1	1	150	90	72	9.4	9.3	9.35	142	9	0.86	135	134	39	79	6.2	0
1	64	31.1	104	1	1	2	1	0	1	131	88	61	8.5	8.1	8.3	105	13	0.8	154	219	43	83	5.9	0
2	68	21.6	86	1	1	1	0	0	1	123	89	70	9.4	9.2	9.3	106	19	0.86	203	116	53	131	6.2	0
1	72	19.3	76	1	1	1	1	1	0	135	92	90	15.5	16.5	16	150	41	1.38	151	75	54	91	8	1

1	55	24.2	85	1	1	1	0	0	0	112	74	66	7.2	7.4	7.3	94	21	0.92	190	77	64	119	5.8	0
2	41	28.3	98	1	1	1	1	0	1	139	89	56	7.4	7.4	7.4	96	15	0.6	244	51	103	136	4.8	1
1	36	31.8	94	1	1	1	1	0	1	152	107	90	6.8	6.7	6.75	94	18	0.97	254	146	49	177	5.5	1
1	53	26.7	97	2	2	1	1	1	1	123	92	70	8.4	8.1	8.25	132	14	0.83	157	187	34	98	6.4	1
1	56	23.8	84	1	1	1	0	0	0	138	89	83	7.9	7.3	7.6	92	13	0.73	178	70	57	112	5.5	0
1	65	28.0	98	1	1	1	1	0	1	150	88	62	7.5	6.7	7.1	91	14	0.86	184	133	42	127	5.5	0
1	44	28.7	100	1	2	2	1	0	0	140	94	57	6.6	6.5	6.55	108	15	0.92	202	184	48	136	5.6	0
1	67	14.4	63	2	1	1	0	0	0	117	74	56	9.3	9.4	9.35	94	13	0.78	215	61	92	120	5.3	1
1	38	26.6	92	2	1	1	1	0	0	154	96	50	6.3	6.5	6.4	93	15	1.1	213	101	72	124	5	1
1	60	23.6	86	2	1	1	1	0	1	145	89	59	8.2	8.4	8.3	105	9	0.93	207	201	51	129	5.5	1
2	45	21.1	75	1	1	1	0	0	0	114	74	62	7.5	7.4	7.45	95	10	0.67	170	63	64	95	5.5	1
1	32	28.7	99	2	2	2	1	0	1	141	96	68	6.6	6.6	6.6	103	12	0.88	188	321	30	111	5.4	0
1	48	24.3	81	1	2	1	1	0	0	127	89	63	7.3	7.3	7.3	92	16	0.91	164	102	52	99	5.1	1
2	82	22.4	84	1	1	1	1	1	1	158	89	66	9	9.1	9.05	115	14	0.65	170	135	52	104	6.5	1
1	51	29.9	106	1	1	1	0	0	1	120	78	56	6.2	5.9	6.05	99	14	0.86	232	111	46	166	5.4	1
1	33	30.8	104	2	1	1	0	0	1	134	86	71	7.6	7.3	7.45	102	10	0.95	198	241	27	136	5.4	0
1	52	27.5	94	1	2	2	1	0	1	138	100	67	6.5	6.2	6.35	99	18	0.84	265	105	50	195	5.7	1
2	65	26.2	89	1	1	1	1	0	0	150	89	71	8	7.9	7.95	115	10	0.61	179	194	54	106	6.1	1
2	61	21.2	82	1	1	1	0	0	0	131	84	68	8.8	8.4	8.6	97	12	0.55	205	118	57	130	6	0
1	65	21.6	82	1	1	1	0	0	1	122	80	72	9	9.3	9.15	119	19	1.23	273	64	58	203	5.7	0
1	53	28.4	100	2	1	1	1	0	1	139	92	98	6.5	6.2	6.35	98	15	0.8	244	157	45	174	6.2	0
1	57	24.8	87	1	1	1	0	0	0	121	81	69	6.2	6.5	6.35	98	16	1.04	209	116	51	132	5.4	0
1	39	24.0	90	2	1	1	1	1	1	122	91	81	8	8	8	162	11	0.81	181	157	59	98	8.1	1
2	37	20.0	79.5	1	1	1	0	0	0	134	79	84	6.8	6.8	6.8	88	8	0.61	144	51	49	89	5.4	1
1	46	26.5	94.5	2	2	1	0	0	1	127	86	64	8.1	7.9	8	113	11	0.84	283	294	54	185	5.2	0
1	68	21.9	80	2	1	1	0	0	1	111	79	71	8	8	8	108	14	0.94	190	142	37	133	6	0
1	48	24.9	90	2	2	1	1	1	1	148	98	66	8.9	8.7	8.8	141	13	1.11	159	216	39	98	6.9	1
2	61	21.4	82	1	1	1	0	0	0	116	80	68	7.3	7.7	7.5	125	14	0.65	216	116	86	108	5.6	0
1	46	21.9	82	1	2	1	1	0	1	156	106	83	8	7.8	7.9	103	18	1.15	197	122	62	124	5.9	1

1	59	24.7	90	1	1	1	1	1	1	145	100	60	7.6	7.7	7.65	117	17	0.93	143	168	37	90	7.2	1
1	58	24.0	88	2	1	1	0	0	1	114	75	62	7.8	7.3	7.55	86	12	1.08	151	117	48	94	5.7	0
1	67	24.5	92.5	1	2	2	1	0	1	119	81	56	7.6	7.9	7.75	87	20	0.95	117	89	56	59	5	1
2	37	19.3	73	1	1	1	0	0	0	121	79	70	6.7	6.5	6.6	113	13	0.5	196	43	80	117	5.5	1
2	52	19.5	68	1	1	1	0	0	0	120	66	54	7.9	7.9	7.9	95	18	1.16	213	64	94	115	4.8	1
2	59	25.6	89.8	1	1	2	0	0	0	114	74	69	7.2	7.6	7.4	91	16	0.63	205	82	52	139	5.6	1
1	56	26.1	88	1	2	1	1	0	0	121	92	63	9	8.7	8.85	95	12	1.04	196	81	51	139	6	1
2	37	18.6	68.5	1	2	1	0	0	0	110	67	61	6.8	7.1	6.95	84	10	0.64	203	40	98	112	5.4	0
1	37	24.4	88	2	2	1	1	0	0	142	91	63	7.9	7.6	7.75	90	12	1.2	218	101	65	152	5	0
2	53	23.1	79	1	1	1	1	0	1	137	96	68	8.8	8.5	8.65	87	16	0.78	202	149	50	130	5.4	1
1	50	24.4	84.3	1	1	2	1	0	0	129	96	53	7.9	7.7	7.8	95	14	0.94	236	169	71	153	5	0
1	58	26.3	85	1	2	2	1	0	1	161	109	56	6.5	6.7	6.6	105	12	1.14	236	214	62	131	5.4	1
1	63	23.2	86	2	2	1	0	0	0	132	87	68	9.4	8.3	8.85	98	11	1	234	111	77	150	5.3	1
2	62	22.1	89.5	1	1	1	0	0	1	131	83	63	8.7	8.7	8.7	97	10	0.74	226	53	60	160	5.7	1
1	51	21.4	82	2	1	1	0	0	0	119	79	70	7.6	7.8	7.7	99	11	0.94	217	148	47	154	5.8	1
2	29	20.8	69	2	1	1	0	0	0	119	69	59	7.3	7.1	7.2	88	10	0.54	174	83	52	114	5.1	1
2	48	22.9	78	1	1	1	0	0	0	110	68	51	5	5	5	90	9	0.66	201	108	66	127	5.4	0
1	68	25.3	87	2	2	1	1	1	1	143	94	83	8.8	8.8	8.8	126	19	0.96	209	74	79	130	5.7	1
2	56	19.8	78	1	1	1	0	0	0	120	74	73	7.8	7.8	7.8	93	11	0.75	206	42	58	147	5.3	0
1	61	27.4	90	1	1	1	1	0	0	147	95	59	8.4	8.6	8.5	104	17	1.11	195	57	51	141	6.4	1
2	58	24.1	85.5	1	1	1	0	0	0	119	82	71	6.5	6.7	6.6	90	13	0.76	193	83	63	113	5.6	0
1	63	23.4	87	2	1	1	1	0	1	121	85	59	8.8	9	8.9	100	15	1.03	141	104	58	79	5.5	1
1	74	22.7	80	2	1	2	0	1	1	119	75	56	9.3	9.4	9.35	83	7	0.74	217	223	40	144	6	1
1	61	24.9	93	2	1	1	1	0	1	135	101	63	8.4	8.3	8.35	92	19	0.98	253	117	39	199	5.9	1
1	36	18.3	76	1	1	1	0	0	0	121	88	64	7.4	7.4	7.4	85	19	1.07	210	88	49	155	5.4	0
1	49	25.1	95	1	1	1	0	0	1	130	85	61	6.6	6.1	6.35	107	14	0.95	172	154	39	116	5.5	0
2	61	27.3	95	1	1	2	1	0	1	142	100	57	8.9	8.4	8.65	104	11	0.61	191	64	86	114	5.4	1
1	42	26.8	92	2	2	1	1	0	1	154	110	61	7.7	7.4	7.55	102	12	0.9	288	558	45	153	5.7	0
1	45	26.4	91	2	1	1	0	0	0	124	88	72	7	6.7	6.85	84	12	1.07	190	125	64	118	5.2	1

1	54	21.8	78	1	2	2	0	0	0	113	81	59	7.6	7.6	7.6	98	9	0.91	186	50	48	129	5	0
2	54	20.5	71	1	1	2	1	1	1	141	79	58	8.6	8.5	8.55	133	12	0.69	180	84	53	118	6.5	1
1	46	26.6	97	2	1	1	1	0	1	159	104	66	7.9	7.7	7.8	121	14	0.82	253	274	71	169	5.8	1
1	57	21.1	82	2	1	2	1	0	0	152	111	88	9.5	8.8	9.15	124	13	0.85	227	163	57	159	5.6	0
2	66	25.0	90	1	1	2	0	0	1	108	71	56	9.1	8.4	8.75	92	16	0.73	153	134	57	91	5.5	1
1	62	21.7	78	2	1	2	0	1	1	127	85	79	10.9	10.9	10.9	96	20	1.02	138	54	65	69	5.9	1
2	59	18.7	72	1	1	1	1	0	1	120	82	56	7.4	7.5	7.45	90	17	0.7	203	97	92	109	5.4	0
2	53	27.2	88	1	1	1	1	0	0	138	92	56	6.6	6.5	6.55	85	12	0.67	198	66	63	142	5.7	1
2	59	22.5	83	1	1	1	1	0	0	159	97	68	7.7	7.5	7.6	94	14	0.77	234	119	65	149	5.4	1
2	60	18.4	70	1	1	1	0	0	0	120	77	61	7	6.9	6.95	88	6	0.56	177	41	84	96	5.7	0
1	63	21.4	83	1	2	1	1	0	0	118	80	54	7.9	8	7.95	99	16	0.84	189	103	67	123	5.8	0
1	35	26.7	94	1	1	1	1	0	1	134	92	77	6.9	6.7	6.8	124	21	1.18	201	370	38	119	6.2	1
1	70	23.5	87	1	1	1	1	1	1	140	92	56	7.6	7.8	7.7	134	17	0.98	169	151	55	100	6.9	0
1	58	25.9	92	1	2	1	1	1	1	137	98	70	11.1	11.1	11.1	181	15	0.92	201	199	37	140	7	1
2	58	23.1	76	1	1	1	1	0	1	119	85	72	8	8.1	8.05	105	13	0.69	174	237	36	88	5.7	0
2	67	20.9	73	1	1	1	0	0	0	103	65	67	7.4	7.5	7.45	92	12	0.68	180	137	81	61	5.6	1
1	55	28.6	102	2	1	1	1	0	0	145	100	50	6.4	6.4	6.4	97	15	1.17	226	199	40	149	5.9	1
2	72	25.8	93	1	1	1	1	0	1	165	102	64	7.5	7.6	7.55	96	8	0.73	253	107	77	155	5.3	1
1	67	25.2	91	1	1	1	1	1	1	112	76	63	7.3	7.6	7.45	109	15	1.06	157	148	64	82	6.5	0
1	61	26.4	97	2	2	1	1	1	0	140	88	69	8.4	8.7	8.55	147	13	0.93	178	165	49	108	7.2	1
2	49	26.6	88	1	1	1	1	1	1	153	104	68	8.7	8.4	8.55	113	14	0.51	226	191	35	167	6.5	1
1	51	20.0	76	2	2	1	0	0	1	110	75	80	7.7	7.5	7.6	92	18	0.84	248	191	54	170	5.2	0
1	42	26.4	90	2	1	1	1	0	1	130	92	70	7.3	7.1	7.2	108	11	0.86	177	360	30	109	6.1	0
1	70	27.2	95	2	1	1	1	0	1	135	92	65	8.4	8.3	8.35	98	18	1.15	209	277	45	108	6.1	0
2	43	19.7	72	1	2	1	0	0	0	116	82	63	6.3	6.8	6.55	91	12	0.71	164	57	63	106	5.4	0
2	63	20.3	71	1	1	1	0	0	0	115	78	58	6.7	6.7	6.7	108	9	0.66	201	82	66	129	5.6	1
1	78	24.7	92	1	1	1	1	0	0	135	86	54	9.7	9.3	9.5	90	18	1.01	183	92	48	122	5.2	1
1	53	22.9	87	1	2	2	1	0	1	136	94	56	7.6	7.7	7.65	97	18	0.89	239	150	69	160	5.4	0
1	57	28.8	93	1	1	1	1	0	0	140	80	75	7.1	6.9	7	86	14	0.85	167	60	73	94	5.6	1

1	45	22.1	78.5	2	1	1	0	0	0	107	65	43	7.5	7.6	7.55	95	16	0.89	153	59	67	90	5.3	0
1	47	29.0	98	2	1	2	1	0	1	131	86	72	6.7	6.3	6.5	95	8	0.8	148	143	66	72	5.8	0
1	60	26.0	90	1	2	2	1	0	1	140	83	46	8	8.1	8.05	105	16	1.09	260	53	87	180	5.5	0
1	48	26.1	89	2	1	1	1	1	1	120	85	80	9.1	8.6	8.85	294	14	0.77	312	329	32	234	11.5	1
2	53	26.1	82	1	1	1	1	0	1	134	95	71	7.4	7.7	7.55	103	14	0.64	248	82	78	173	5.8	0
1	55	24.5	88	1	1	1	0	0	1	131	85	82	7	7.1	7.05	105	12	0.91	233	100	60	168	5.4	1
1	42	25.2	87	2	2	1	0	0	0	122	83	51	6.2	6.4	6.3	94	18	0.94	213	71	65	154	5.7	0
1	61	31.9	99	2	1	2	1	0	1	126	77	65	6.2	5.8	6	108	12	0.94	277	173	54	182	5.4	0
1	62	25.7	87.5	1	1	1	1	0	0	154	105	52	8	7.1	7.55	101	18	0.95	192	77	61	131	5.4	1
1	57	29.4	99	1	1	2	0	0	0	134	84	57	7.3	6.6	6.95	97	15	0.96	169	81	69	93	5.5	1
2	57	20.9	77	1	1	1	0	0	0	121	79	49	8.3	7.8	8.05	87	14	0.59	238	79	64	151	5.4	0
1	62	24.4	88	2	1	2	1	0	1	144	93	65	9.4	9.7	9.55	113	19	0.91	207	231	54	115	5.5	1
1	62	23.0	90	2	2	2	1	0	0	142	93	70	8.8	8.8	8.8	100	18	0.99	210	74	57	138	5.2	1
2	60	24.0	87	1	1	2	1	0	1	134	89	61	9.6	9.2	9.4	91	9	0.65	234	110	56	160	5.5	1
1	59	24.1	92	1	1	1	1	1	0	126	87	64	8.2	8.1	8.15	129	12	1.22	210	95	62	148	6.5	1
1	49	23.7	83	1	1	1	0	0	1	126	78	58	6.9	7	6.95	109	16	0.89	227	172	53	166	5.8	0
1	54	22.7	83	2	2	1	1	1	1	129	91	70	6.9	7.1	7	128	18	0.92	289	97	69	216	5.9	0
1	55	26.4	87	1	2	2	0	1	1	129	89	51	8.9	8.3	8.6	140	14	1.18	206	158	38	144	5.8	0
1	41	29.0	103	2	1	1	1	0	1	148	98	63	7.6	7.8	7.7	105	14	0.93	184	243	41	123	5.5	1
2	54	21.5	78	1	1	2	0	0	0	121	75	55	7.2	7.2	7.2	95	12	0.66	202	53	56	142	5.4	1
1	64	22.5	80	2	2	1	0	0	0	126	79	67	9.6	15	12.3	95	13	0.71	208	62	63	144	5.6	0
1	60	26.3	96	2	1	1	1	0	1	140	97	82	9.3	14.4	11.85	109	17	0.97	276	207	51	205	5.8	0
1	63	23.2	88	2	1	1	1	1	1	144	90	67	9	8.8	8.9	272	11	0.9	106	152	31	64	12.9	0
1	52	26.8	89	1	1	2	0	0	0	126	88	53	6.6	6.8	6.7	106	14	1.05	199	157	47	138	5.7	1
1	54	23.2	82	1	1	1	0	1	1	127	83	64	7.5	7.4	7.45	135	11	0.99	160	84	63	97	6.6	1
2	58	23.0	87	1	1	1	0	0	0	125	88	78	8.7	9	8.85	111	12	0.69	167	129	49	105	5.4	0
1	51	29.0	95.5	2	2	2	1	0	0	141	98	73	4.7	5.1	4.9	113	11	0.86	185	159	40	136	5.6	0
1	56	24.1	84	2	2	1	0	0	0	113	78	69	6.7	7	6.85	101	10	0.66	157	149	44	97	6.4	0
1	48	23.5	86	1	1	2	1	0	0	145	94	59	6.9	7	6.95	94	17	0.91	182	42	73	115	5.4	1

1	52	24.5	92.5	1	1	2	0	0	1	125	78	77	7.1	7	7.05	106	13	0.96	166	93	45	119	5.1	1
2	46	28.9	95	1	1	1	1	0	0	124	92	81	7.8	7.5	7.65	102	11	0.61	216	132	56	156	5.9	1
2	49	25.2	82	1	1	1	0	1	1	116	77	69	7.8	7.5	7.65	110	10	0.57	229	84	56	174	6.6	1
1	69	26.6	86	2	1	1	1	0	1	162	105	82	8.4	7.8	8.1	110	11	1.02	159	217	29	102	4.7	1
2	47	19.2	69	1	1	1	0	0	0	127	82	55	7.4	7.5	7.45	91	11	0.72	169	83	109	62	5.3	0
1	35	26.8	93	2	1	1	1	0	1	131	97	77	6.9	6.9	6.9	115	13	1.02	222	241	41	159	5.7	1
1	59	27.3	97	1	1	1	1	1	1	145	93	63	7.7	7.7	7.7	129	20	1.3	118	101	41	62	7.9	1
1	52	30.1	104	1	1	1	1	0	1	143	99	95	6.7	6.7	6.7	111	13	0.94	144	82	58	90	5.8	0
1	54	24.3	88	2	2	1	0	0	0	106	75	63	9.2	9.1	9.15	96	16	1.01	195	58	71	128	5.6	0
1	54	23.2	81	2	2	1	1	0	0	149	108	90	9.3	10.4	9.85	113	15	0.68	216	86	66	152	5.4	1
1	58	24.1	83	1	1	1	0	0	0	129	80	63	7.3	7.6	7.45	102	19	0.85	159	74	40	114	5.6	1
1	47	25.1	80.5	2	1	2	1	0	1	142	89	72	6.8	6.7	6.75	104	15	0.94	183	171	38	134	5.7	1
1	56	22.6	84.5	2	1	1	1	1	1	137	96	62	7.2	6.7	6.95	104	13	0.85	218	198	66	137	6.8	1
2	41	21.0	75	1	2	1	1	0	0	133	91	96	5.5	5.7	5.6	96	8	0.54	163	37	79	87	4.9	1
1	54	26.8	93	2	2	1	1	0	1	134	96	62	7.6	7.6	7.6	95	18	1.16	187	369	36	106	5.4	0
2	57	22.4	77	1	1	1	1	1	1	146	98	69	8	7.1	7.55	158	9	0.57	221	165	53	150	10.3	0
1	49	26.1	93	1	2	1	1	0	0	138	102	64	6.2	6.3	6.25	114	14	1.11	173	124	45	120	5.7	1
1	54	25.6	91	1	1	1	1	1	1	146	108	79	8.7	9.2	8.95	152	13	1.01	179	170	42	118	6.7	0
1	58	22.4	83	2	1	2	0	0	1	121	79	57	7.3	7.3	7.3	79	14	0.9	214	163	32	162	5.5	1
1	59	25.2	87	1	2	2	0	0	0	116	83	63	7	7.3	7.15	95	16	0.95	203	53	47	152	5.1	1
1	61	24.7	86	1	2	1	0	1	1	136	79	47	7.6	7.7	7.65	107	22	1.29	196	76	85	114	6.5	1
2	50	20.2	69	1	1	2	0	0	1	121	82	90	6.9	7.4	7.15	88	13	0.72	253	51	70	182	6	0
2	56	19.2	67	1	1	1	0	0	1	124	78	63	7.2	7.5	7.35	105	13	0.74	247	61	68	174	5.9	1
1	52	22.8	79	2	1	1	0	0	0	136	83	57	6.8	7.2	7	100	14	0.91	189	140	64	115	5.4	0
1	59	21.7	79	1	1	2	0	0	1	124	69	54	7.9	7.9	7.9	92	43	1.05	153	74	25	113	5.1	1
1	47	25.2	85	1	1	1	1	0	1	140	81	66	7.2	7	7.1	97	14	1.06	147	108	34	99	5.4	0
2	74	22.3	78	1	1	1	1	1	1	143	73	69	10.5	10.8	10.65	131	22	0.87	168	246	48	90	7.5	1
2	64	24.0	82	1	1	1	0	0	0	113	66	59	6.7	6.8	6.75	82	17	0.73	158	127	73	72	5.6	0
2	45	20.8	78	1	1	1	1	0	1	128	90	67	7.2	7.2	7.2	94	12	0.62	283	48	64	215	5.5	0

2	63	23.4	85	1	1	1	1	0	0	158	102	101	8.8	9.1	8.95	104	8	0.58	206	114	67	125	5.9	1
1	75	23.4	90	1	1	1	1	0	1	137	90	67	9.6	9.9	9.75	111	15	1.11	195	277	36	122	5.7	1
1	37	24.5	81	1	1	2	1	0	0	149	98	91	5.9	6.2	6.05	90	15	1.01	135	47	41	95	5.7	1
1	57	20.6	85	2	2	1	1	1	0	162	105	96	10.1	9.8	9.95	150	20	0.73	123	152	59	52	7.3	0
2	26	23.1	77	1	1	1	0	0	0	106	71	62	7	7.4	7.2	95	9	0.7	148	56	66	80	5.4	0
1	50	22.1	86	1	2	1	0	0	0	120	77	50	7.2	7.4	7.3	99	14	0.85	209	125	45	157	5.6	0
1	57	23.7	90	2	2	1	1	0	0	136	95	76	7.3	7.4	7.35	101	13	0.75	196	130	42	143	5.5	1
2	54	21.3	73	1	1	2	0	0	0	138	78	56	6.7	7	6.85	89	12	0.7	207	98	69	134	5.4	1
2	50	22.1	79	1	1	1	0	0	0	122	76	52	7	7.1	7.05	89	10	0.62	203	130	65	127	5.4	1
1	55	21.7	81	2	1	1	1	0	1	142	101	61	7.8	7.7	7.75	100	13	0.92	235	241	37	153	5.8	0
1	51	21.6	84	2	1	1	0	0	1	114	80	57	7.6	7.7	7.65	103	19	1.12	251	123	40	200	5.5	0
2	54	26.6	88	1	1	1	1	0	1	140	88	70	7.8	7.6	7.7	104	13	0.78	227	172	41	169	5.4	0
1	46	21.7	82	2	2	2	1	0	1	154	108	67	7.2	7.3	7.25	87	13	0.94	257	191	65	172	5.3	1
1	63	25.1	94	2	1	1	1	0	1	143	101	72	8.9	9.7	9.3	109	14	1.25	227	130	58	164	5.8	1
1	53	34.4	112	1	1	1	1	1	1	150	86	50	8.3	8.2	8.25	234	12	0.9	164	106	37	106	8.8	1
2	53	37.4	104	1	1	1	0	0	1	131	86	69	6	5.5	5.75	93	16	0.76	243	132	48	174	5.7	1
2	59	22.4	79	1	1	2	0	0	0	117	72	54	7.6	7.9	7.75	99	15	0.74	146	52	50	92	5.4	0
1	58	32.6	107	1	1	1	1	1	0	138	98	82	7.8	8	7.9	126	8	0.8	137	58	63	67	6.5	0
1	54	21.2	78	1	1	1	0	0	0	113	71	62	7.1	7.3	7.2	100	11	0.91	205	36	80	130	5.6	1
1	46	23.7	83	1	2	2	1	0	1	125	97	65	7.1	6.9	7	91	13	0.91	251	180	76	154	5.4	0
1	45	26.1	84.5	2	1	1	1	0	1	149	98	59	7.7	7.3	7.5	101	17	0.86	208	136	32	164	6.1	0
2	41	25.4	93	1	1	1	0	0	0	121	84	71	6.7	6.7	6.7	88	9	0.62	229	126	80	139	5.2	1
1	40	35.3	110	1	2	1	1	0	0	150	100	86	5.9	6	5.95	104	12	1.01	207	137	78	116	5.5	0
1	54	25.2	87	2	1	1	1	0	1	132	91	75	7.6	7.8	7.7	100	19	1.18	228	246	38	113	6.1	0
1	39	25.7	92	2	2	1	1	0	1	142	87	57	7.5	7.5	7.5	100	12	1	273	165	58	135	5.3	1
1	63	21.6	79	1	1	1	1	0	0	147	102	70	8	7.7	7.85	99	12	0.92	194	60	49	129	4.8	1
2	56	23.7	79	1	1	1	0	0	0	138	84	68	7.6	7.6	7.6	84	11	0.68	201	102	74	105	6.1	0
1	60	24.7	87	2	1	1	1	1	0	148	101	60	9.3	9.5	9.4	113	17	1	209	174	42	146	6.2	0
1	68	24.5	94	1	1	1	1	1	1	122	82	63	8	8	8	126	13	0.8	127	64	54	70	6.9	1

2	57	21.1	79	1	2	1	1	0	1	140	81	52	7.2	7.5	7.35	93	11	0.78	197	42	90	102	5.7	0
2	56	19.5	72	1	1	1	0	0	1	121	86	66	7.4	7.4	7.4	98	14	0.67	237	132	51	160	5.7	1
1	72	20.7	82	2	1	1	0	0	1	139	85	62	9.2	9.4	9.3	89	21	1.13	260	184	64	163	6.1	0
2	40	18.4	65	1	1	1	0	0	0	119	80	67	7.4	7.6	7.5	94	11	0.56	163	70	54	103	5.4	0
1	65	26.2	97	2	2	1	1	0	1	144	86	55	9.5	9.3	9.4	90	12	1.02	125	103	45	61	5.9	1
1	50	24.8	87	1	1	1	0	1	1	127	87	101	7.1	7.1	7.1	170	9	0.69	143	144	58	73	10.1	0
1	43	26.2	90	2	1	1	1	0	1	131	90	51	6.8	6.5	6.65	86	10	0.96	204	152	37	140	5.4	1
2	83	22.1	79	1	1	1	0	1	1	138	86	71	11.7	11.4	11.55	134	6	0.62	196	383	45	106	7.6	0
1	47	23.2	88	2	1	1	0	0	1	112	81	66	7.1	7	7.05	83	16	1.21	235	62	51	167	5.6	1
2	60	26.3	92	1	1	1	1	0	0	140	80	85	7.9	8.2	8.05	106	10	0.66	221	62	77	133	5.4	1
2	49	18.9	72	1	1	1	0	0	0	117	72	67	7.2	7.3	7.25	103	17	0.8	199	99	70	116	5.2	0
1	65	26.5	91	2	2	1	1	0	1	143	89	55	8.6	9	8.8	111	16	0.96	205	210	37	133	5.7	1
2	61	19.4	76	1	1	1	1	0	0	150	96	74	7.8	8.5	8.15	92	20	0.88	173	25	59	113	5.7	1
1	43	25.0	90	2	1	1	1	1	1	148	102	85	7.2	7.3	7.25	136	8	0.91	157	136	27	89	5	0
1	46	23.5	85	1	1	2	1	0	0	150	95	45	7.4	7.3	7.35	93	13	0.91	180	137	57	102	5.3	1
2	40	23.7	80	2	1	1	0	0	0	111	67	75	5.1	5.1	5.1	82	14	0.62	156	42	64	103	5.1	1
1	55	27.8	102	1	2	2	1	0	0	146	94	66	9.2	9.7	9.45	98	12	1.14	215	147	49	131	5.4	1
1	49	23.8	90	1	1	2	0	1	0	130	84	66	7.9	7.9	7.9	144	14	0.7	161	142	46	97	7.5	1
2	46	18.4	69	1	1	1	0	0	1	114	72	73	6.9	7.3	7.1	88	12	0.65	243	67	112	102	5.3	0
1	46	26.3	91	1	1	1	1	0	1	143	87	62	7	7	7	87	15	1.04	202	73	35	155	5.3	0
1	43	20.5	76	1	1	2	0	0	0	118	78	52	6.8	6.8	6.8	85	11	0.9	239	39	92	136	5	0
1	48	27.0	96	1	1	1	1	1	1	165	70	76	7	6.9	6.95	145	13	0.84	176	228	63	78	6.5	1
2	59	19.7	70	2	1	1	0	0	0	105	73	65	7.1	7.1	7.1	90	14	0.67	170	69	67	89	5.8	1
1	56	36.9	120	2	1	1	1	0	1	132	91	63	7.1	7.5	7.3	106	17	0.89	201	126	27	152	6.3	1
2	58	26.2	83	1	1	2	1	0	0	132	95	74	8.1	7.7	7.9	96	16	0.84	217	63	76	122	5.8	1
2	79	28.9	95	1	1	1	1	0	0	148	100	60	9.7	8.8	9.25	117	17	0.7	235	138	58	144	6.4	1
2	56	25.0	82	1	1	1	1	0	1	156	95	70	8.5	8.8	8.65	101	14	0.76	225	71	61	150	5.9	1
2	38	23.2	78.5	1	1	1	0	0	0	108	68	87	6	6.1	6.05	88	11	0.6	195	34	46	139	5.3	0
1	48	32.7	111	2	1	1	1	0	1	140	103	69	6.8	6.9	6.85	95	19	0.97	154	330	35	76	5.5	0

1	60	22.9	81	1	1	2	0	0	0	112	77	58	8	7.9	7.95	86	15	0.98	160	40	40	125	5.4	1
1	50	32.6	104	1	2	1	1	1	0	166	114	64	7.8	7.4	7.6	126	16	0.84	187	112	54	116	6.1	0
2	55	21.1	79	1	1	1	0	0	0	113	69	73	7.9	7.8	7.85	100	14	0.69	176	127	65	85	5.7	0
2	47	18.7	74	1	1	1	0	0	0	119	71	67	7.3	7.5	7.4	98	10	0.67	210	67	64	133	5.3	1
1	55	22.6	82	1	2	1	1	0	1	136	89	74	7.6	7.5	7.55	92	9	0.72	161	122	54	95	5.5	1
2	58	20.8	78	2	1	1	0	0	1	103	66	70	7.1	7.1	7.1	96	14	0.71	256	148	69	139	5.4	1
1	72	24.2	92	2	1	1	1	0	0	147	104	63	10.4	10.7	10.55	89	13	0.99	205	146	42	124	5.3	1
1	62	32.0	110	1	1	1	1	0	0	145	99	76	9.1	8.9	9	99	12	0.94	206	66	48	155	5.4	1
1	71	23.4	88	1	1	1	1	0	1	143	90	55	9.2	9.1	9.15	97	13	0.83	146	81	66	65	5.4	1
2	48	24.1	79	1	1	1	0	0	1	118	80	58	6.1	6.2	6.15	90	13	0.71	299	106	79	180	5.3	0
1	59	23.3	92	2	1	1	0	0	0	136	85	97	7.4	7.9	7.65	86	18	0.98	237	48	48	115	5.6	1
1	43	28.2	94	2	1	1	1	0	1	159	95	76	8.1	8.1	8.1	107	15	0.88	197	519	38	92	5.5	1
1	65	26.3	92	1	1	1	1	1	1	106	75	65	7.9	8	7.95	150	17	1.12	184	134	38	120	6.7	1
2	51	20.8	70	1	1	1	0	0	0	113	70	57	7.9	8.3	8.1	87	11	0.71	217	70	61	144	5	1
1	58	26.7	94	2	2	1	1	0	0	150	96	75	9.3	9.5	9.4	103	12	0.92	183	69	50	115	5.7	1
1	67	23.9	90	2	1	1	1	1	0	139	92	84	10.2	10.2	10.2	186	20	1.34	138	46	65	72	6.9	0
1	62	21.8	80	2	1	1	1	0	1	156	106	63	8.6	8.9	8.75	99	12	0.97	179	135	44	118	5.4	0
2	59	19.5	69	1	1	1	1	1	0	123	78	66	7.8	7.7	7.75	99	13	0.77	203	105	59	123	5.4	1
2	56	21.5	70	1	1	1	0	0	1	129	81	70	6.7	6.7	6.7	113	10	0.65	253	43	106	130	5.6	1
1	61	23.2	88	2	1	1	0	1	1	130	82	50	8.1	8.4	8.25	137	16	0.93	130	122	39	70	6.8	0
1	58	25.5	98	2	1	1	0	1	1	123	82	75	8.5	8.3	8.4	213	15	1.05	190	212	50	109	9.4	1
1	63	31.6	107	1	1	1	0	1	0	127	86	75	6.4	6.3	6.35	116	12	0.97	178	114	47	117	6.5	0
1	58	22.6	86	1	1	1	1	0	0	130	91	63	7.4	7.4	7.4	89	14	1.02	205	47	73	129	5.2	1
1	75	22.4	85	1	1	2	1	1	1	146	80	66	9.7	9.7	9.7	202	18	0.9	109	56	34	71	7.6	1
2	56	31.6	98	1	1	1	1	0	0	143	98	66	8.2	8.3	8.25	77	17	0.51	198	196	50	123	6	1
1	39	20.9	79	2	1	1	1	0	0	140	86	62	6.8	6.9	6.85	96	12	0.98	209	92	110	93	5.1	0
1	41	18.9	75	2	2	1	1	0	0	138	91	83	6.8	6.7	6.75	107	8	0.89	208	159	90	108	5.4	0
2	65	23.1	80	1	1	1	1	0	1	140	89	70	8.1	8.7	8.4	94	20	0.72	244	87	61	160	5.4	1
2	74	21.2	80	1	1	1	1	0	1	156	94	46	9.3	9.4	9.35	89	18	0.51	218	81	73	128	5.7	1

1	54	20.0	74	1	2	2	1	0	0	131	94	56	7.5	7.6	7.55	98	19	0.98	161	63	43	111	5	0
1	64	26.7	92	1	2	2	1	0	0	143	98	59	7.1	7.5	7.3	90	16	0.89	205	75	75	115	5.4	1
2	53	23.7	88	1	1	1	0	0	1	107	70	67	8	8.2	8.1	92	12	0.73	276	531	39	93	5.8	0
1	59	24.8	91	1	2	1	1	1	0	140	92	70	7.6	7.6	7.6	130	17	0.77	207	32	68	131	5.4	1
1	51	24.6	83	1	1	2	0	0	0	129	83	53	7.1	7.2	7.15	94	13	0.81	177	65	51	108	5.3	1
1	57	21.6	84	1	2	1	1	0	1	125	87	83	7	7.1	7.05	92	12	0.93	191	37	56	135	4.7	1
2	60	23.0	82	1	1	1	1	0	0	124	79	65	7.9	8.3	8.1	92	16	0.75	239	28	82	151	5.6	0
1	63	21.9	76	1	1	1	0	0	0	124	82	76	7.8	8.1	7.95	108	14	0.96	218	93	58	139	5.8	1
1	55	24.5	92	2	2	1	1	0	1	142	100	54	6.1	6	6.05	107	15	1.18	233	164	49	168	5.7	0
2	61	20.4	80	1	1	1	0	0	1	127	81	72	8.9	8.6	8.75	92	12	0.73	246	130	54	156	5.4	1
1	41	25.0	91	2	2	1	0	0	1	131	87	50	7.4	7.5	7.45	113	12	0.75	180	236	48	99	5.2	0
2	69	21.3	83	1	1	1	0	0	0	109	77	65	8.6	8.6	8.6	104	13	0.84	184	49	63	108	5.4	0
1	47	26.8	95	2	1	1	1	1	1	113	79	66	7.4	7.6	7.5	60	24	1.32	163	153	46	92	6.8	0
1	58	21.5	79	1	1	1	0	0	0	121	78	72	6.7	6.9	6.8	72	14	0.7	195	48	47	133	5.6	1
2	57	22.0	79	1	1	1	0	1	0	125	81	67	7.1	7.3	7.2	239	13	0.56	199	122	43	151	6.8	0
1	56	25.0	88	1	2	1	1	0	1	144	92	78	6.9	6.9	6.9	98	13	1.01	240	71	67	144	5.4	1
2	62	28.8	92	1	1	1	1	1	1	113	79	71	7.8	7.5	7.65	124	14	0.77	259	116	44	196	6.5	0
1	60	23.9	90	2	2	1	1	1	1	138	93	67	9	9.1	9.05	170	17	0.92	213	187	35	144	7.7	1
1	51	27.9	95	1	1	1	1	1	1	126	84	58	6.9	6.7	6.8	139	13	0.99	229	166	44	157	6.5	1
1	44	18.3	75	2	1	1	0	0	0	90	60	56	8.1	8.5	8.3	88	12	1.09	149	43	64	72	5.3	0
1	53	26.6	81	2	1	1	0	0	1	132	88	70	7.1	7	7.05	99	17	0.88	230	53	68	176	5.4	1
1	53	25.0	93	1	2	2	1	0	0	141	102	56	8.1	7.1	7.6	92	15	1.04	215	96	56	126	5.5	1
2	47	23.2	81	1	1	1	0	0	0	116	60	56	6.9	6.8	6.85	96	15	0.67	183	70	59	103	5.4	0
2	72	22.8	86	1	1	1	1	1	1	138	90	64	8.5	8	8.25	173	12	0.61	186	119	48	111	10.7	1
1	68	23.9	87	1	1	1	0	0	1	126	71	60	7.7	7.9	7.8	124	15	0.9	239	100	51	169	5.8	1
1	66	24.9	94	1	1	1	1	1	0	146	89	53	8.4	8.7	8.55	144	10	0.8	237	137	51	111	6.7	0
2	54	22.9	80	1	1	2	1	0	0	138	101	59	8.8	8.8	8.8	92	11	0.84	197	126	64	111	5.5	1
2	70	21.6	84	1	1	1	1	0	0	147	89	65	8.3	8.3	8.3	96	18	0.67	186	96	54	104	5.6	1
1	49	20.5	75	1	1	1	0	0	1	114	69	60	7.1	7.2	7.15	95	8	0.8	266	99	91	147	5.7	1

1	46	23.2	89	1	2	1	1	0	1	138	92	59	7	7.3	7.15	105	10	0.96	253	187	44	175	5.4	0
1	49	28.0	97	1	2	1	1	0	1	130	85	70	6.6	6.7	6.65	107	10	0.76	140	112	46	84	5.6	1
1	58	26.1	95	1	2	2	1	0	1	136	92	79	8.4	8.1	8.25	111	13	0.97	173	105	57	105	5.4	1
1	60	26.3	94	1	1	1	1	1	0	156	103	65	8.1	7.2	7.65	118	12	0.96	216	86	44	152	6.5	0
2	61	21.8	79	1	1	1	0	0	0	131	89	60	9.4	8.4	8.9	86	13	0.7	230	167	76	127	6	1
1	62	24.4	86	2	1	2	1	0	0	148	100	57	9.4	8.1	8.75	94	12	1.01	224	187	56	121	5.3	1
1	56	23.6	91	1	1	1	0	0	0	110	74	60	6.8	6.9	6.85	93	14	0.97	196	125	52	114	5.4	0
2	48	29.3	99	1	1	1	0	0	0	120	70	69	5.1	5	5.05	95	7	0.56	159	60	55	96	5.9	1
1	47	26.8	93	2	2	1	1	0	1	150	95	58	7	6.5	6.75	105	15	0.92	174	218	40	59	5.4	1
1	65	24.4	88	2	1	1	1	0	0	154	95	53	8.5	7.5	8	111	15	1	205	163	41	133	5.7	1
1	47	22.9	83	2	2	1	1	0	1	127	86	58	6.4	6.6	6.5	114	11	0.82	177	83	66	102	5.8	1
1	47	25.6	88	2	2	1	1	0	1	130	87	72	6.5	6.3	6.4	110	12	1.12	167	261	68	66	5.4	1
1	53	20.8	73	2	1	1	1	0	0	133	98	68	8	8.1	8.05	101	12	0.95	192	60	71	99	5.3	0
2	65	20.2	75	1	1	1	1	0	0	160	96	70	9	8.9	8.95	109	13	0.63	216	60	90	112	5.7	1
1	43	28.1	98	2	1	2	1	0	1	137	93	69	6.1	6.1	6.1	95	11	0.78	271	148	44	189	5.5	1
1	46	24.8	91	1	1	1	0	0	0	127	86	77	7.2	7	7.1	95	13	0.92	227	99	53	145	5.1	0
2	67	25.4	91	1	1	2	0	0	0	115	72	57	9.5	9.2	9.35	103	10	0.64	220	128	65	120	5.8	1
1	69	25.7	86	2	1	1	1	0	1	145	95	127	9.8	8.2	9	102	13	1.09	216	399	41	116	5.7	0
2	68	20.4	73	1	1	1	0	0	0	135	76	53	8.6	8.6	8.6	90	19	0.8	232	59	63	157	5.7	0
1	60	19.6	72	2	1	1	1	1	1	151	96	76	8.4	9.1	8.75	146	20	1.42	167	98	65	80	6.5	1
2	41	20.0	77	1	1	1	0	0	0	93	63	78	6.7	6.5	6.6	86	11	0.72	237	38	81	138	5.4	0
1	66	25.0	92	1	2	1	1	0	0	140	96	45	8.4	8.1	8.25	103	14	0.98	224	129	47	150	5.4	1
2	76	22.2	82	1	1	1	1	0	0	147	90	59	9.8	9.8	9.8	95	12	0.81	216	81	66	127	5.4	1
1	58	29.1	101	1	2	1	1	0	1	145	97	54	7	6.9	6.95	86	14	0.96	247	59	81	165	5.5	1
1	65	15.9	65	2	2	2	1	0	0	131	92	54	9.7	9.4	9.55	64	27	0.71	169	62	70	92	5.1	1
1	79	22.2	82	1	1	1	1	1	1	144	76	53	10.8	10.3	10.55	132	10	0.87	213	171	45	134	6.4	1
1	61	30.5	99	1	1	1	1	0	1	121	89	98	6.8	6.6	6.7	116	11	1.22	193	201	46	141	5.6	0
1	54	19.0	74	2	2	1	0	0	0	123	83	63	8.2	8.5	8.35	118	19	0.86	200	62	72	115	5.7	1
2	48	19.8	75	1	1	1	0	0	0	109	70	61	6.5	6.7	6.6	95	8	0.68	194	100	71	109	5.3	0

1	50	31.2	95	1	1	2	1	0	1	150	93	62	6.9	6.7	6.8	97	20	0.79	247	156	49	165	6	0
1	52	27.1	91	1	1	2	0	0	0	126	74	52	7.4	7.4	7.4	105	16	1.07	216	199	44	126	5.6	1
1	48	21.6	78	2	2	2	0	0	0	114	82	59	7.1	7.4	7.25	100	10	0.93	223	131	66	133	5.3	1
1	55	23.1	83	2	1	1	0	0	0	120	83	59	7.4	7.4	7.4	88	12	0.86	171	123	53	130	5.8	0
2	70	21.2	83	1	1	1	0	1	1	132	82	70	8.5	8.2	8.35	154	19	0.7	143	80	49	82	7	1
1	67	23.8	94	2	2	1	1	1	0	128	92	76	9.4	9.2	9.3	175	13	0.74	238	150	62	147	10.8	0
1	54	24.7	90	2	1	1	1	1	1	126	84	60	7.2	7.2	7.2	137	15	0.78	114	95	46	57	7.5	1
1	45	25.0	93	1	2	1	1	0	0	119	78	64	7	7	7	113	21	1.11	207	166	45	137	5.6	0
2	69	23.0	87	1	1	1	1	1	1	139	87	71	9.4	9.3	9.35	124	19	0.76	156	154	37	100	6.8	0
1	36	38.0	112	1	1	1	1	1	1	127	64	63	4.5	4.1	4.3	150	15	1	228	146	37	163	8.1	1
2	51	21.5	74	1	1	2	0	0	0	112	82	58	7	7	7	90	11	0.62	209	70	70	117	5.4	0
1	53	25.9	84	2	2	1	0	0	0	124	80	66	7.1	7.4	7.25	99	10	0.95	185	122	62	113	5.8	1
1	54	26.6	90	1	1	1	1	0	1	149	91	67	6.7	6.7	6.7	115	16	1	259	308	54	149	5.9	1
1	53	21.5	79	1	2	1	1	1	0	131	91	52	7.4	7.3	7.35	125	14	1.07	148	80	79	64	5.8	1
1	48	24.6	95	1	1	1	1	0	1	140	112	82	8	7.9	7.95	106	20	1.16	235	153	40	177	5.5	1
1	55	27.3	91	1	2	1	1	0	0	145	94	66	8	7.9	7.95	100	11	0.79	232	128	59	147	5.5	1
1	53	23.2	86	1	2	2	1	0	1	137	97	74	7.5	7.6	7.55	105	15	1.06	158	190	51	82	5.5	0
2	50	27.1	90	1	1	1	0	0	0	139	84	75	7.3	7.1	7.2	94	12	0.69	211	159	41	132	5.8	0
1	55	25.9	94	1	2	1	1	0	1	111	75	57	7.5	7.5	7.5	98	18	0.98	173	96	70	91	6	0
1	55	25.3	94	1	1	1	1	0	0	134	90	64	7.2	7.3	7.25	110	19	1.08	205	138	47	127	5.5	0
1	55	22.9	81	1	1	1	0	0	1	122	85	60	6.9	7.1	7	96	19	0.98	198	65	47	136	5.9	1
2	42	21.6	84	1	1	1	0	0	0	105	71	64	7	7.1	7.05	81	9	0.52	159	84	59	86	5.4	1
2	49	24.0	82	1	1	2	0	0	1	129	83	58	5.8	5.9	5.85	107	14	0.74	255	188	60	158	5.9	1
1	56	24.2	89	1	2	1	0	0	1	129	85	75	8.1	8.2	8.15	111	12	0.87	248	192	57	153	5.7	0
2	57	28.5	90	1	1	2	1	1	1	142	96	51	8.9	8.5	8.7	118	14	0.6	170	134	42	107	6.3	1
1	61	26.2	89	2	1	2	1	1	1	163	74	59	9.7	9.3	9.5	163	10	1.01	159	144	32	109	7.4	1
1	56	23.5	91	2	2	1	0	0	1	117	77	53	7.9	8.1	8	108	10	0.9	192	219	43	111	6	1
2	62	19.6	73	1	1	2	1	0	0	143	99	63	7.5	7.3	7.4	81	16	0.6	211	48	64	133	5.4	0
1	54	25.3	84	2	1	1	1	0	1	150	111	75	8.6	9	8.8	103	12	0.84	241	101	50	157	6	1

1	67	27.2	97	2	1	2	1	1	1	132	75	63	8.1	8.2	8.15	163	15	0.94	152	88	67	63	7.2	0
2	71	23.5	82	1	1	1	0	1	1	138	74	68	9.4	9.4	9.4	109	12	0.62	150	92	50	82	6.7	1
2	65	23.5	83	1	1	1	1	0	1	132	81	60	7.8	7.7	7.75	106	11	0.77	234	179	44	151	5.6	1
2	63	21.4	81	1	1	1	1	0	1	142	75	56	8.1	8.4	8.25	96	17	0.62	246	92	65	158	5.8	1
2	73	22.5	81.5	1	1	2	1	1	1	145	79	68	9.6	9.7	9.65	145	13	0.69	270	431	47	127	6.9	1
2	57	23.7	83	1	1	1	1	0	1	139	87	62	8.4	8.2	8.3	91	11	0.88	212	123	56	125	5.6	1
2	55	20.8	76	1	1	1	0	0	0	102	67	67	8.5	8.5	8.5	103	9	0.57	173	88	59	96	5.4	1
1	45	23.5	80	2	2	1	1	0	0	138	93	53	7.2	7.1	7.15	108	12	0.9	227	156	49	142	5.3	0
2	49	20.5	72	1	1	1	0	0	0	122	87	79	7.2	7.2	7.2	92	9	0.61	171	125	56	87	5.6	1
1	50	24.8	91	1	2	1	0	0	0	123	83	71	7.5	7.7	7.6	117	13	0.72	191	187	42	122	6.2	1
1	52	23.0	82	2	2	1	1	0	1	147	89	58	8.3	8.4	8.35	104	12	0.86	246	164	61	152	5.6	1
1	48	21.8	83	2	1	1	0	0	1	102	76	55	8.6	8.8	8.7	83	14	0.84	249	56	79	156	5.3	1
2	42	22.2	78.5	1	1	2	1	0	0	159	99	70	4.8	4.9	4.85	85	7	0.62	185	62	69	104	5.1	0
1	72	30.5	104	1	1	2	0	0	0	130	77	53	8.3	8.4	8.35	99	17	0.76	197	68	79	113	6	0
1	55	25.9	95	2	2	1	1	0	1	135	98	66	7.5	7.4	7.45	122	13	0.77	174	260	46	98	6.2	1
1	48	30.5	99	2	2	1	1	0	1	136	94	63	8.3	7.9	8.1	119	11	0.76	207	294	43	127	5.6	0
1	49	25.1	88	1	2	1	0	0	0	115	76	61	6.8	6.7	6.75	92	13	1.01	220	84	49	142	5.6	0
1	56	22.4	84.5	1	2	1	1	0	0	130	86	68	6.1	6.1	6.1	110	14	0.95	130	100	54	66	5.4	0
1	68	25.5	89	2	1	1	1	1	1	154	104	59	9.7	9.5	9.6	121	18	1.18	168	56	55	108	6.7	0
1	64	24.0	97	1	1	1	1	0	1	117	86	67	8.1	8.1	8.1	88	18	1.21	113	83	38	67	5.8	0
1	63	27.1	93	2	1	2	1	1	0	116	74	68	7.1	7.3	7.2	153	15	1.33	146	174	42	75	8.7	0
1	60	28.1	103	2	1	1	0	1	1	129	82	64	6.7	6.6	6.65	112	12	0.93	174	55	48	118	6.7	0
1	87	20.6	82	2	1	1	1	0	0	170	104	36	9.6	8.6	9.1	84	24	1.03	196	41	65	117	5.4	0
1	63	21.9	77	2	1	2	0	0	1	113	76	51	8.5	8.3	8.4	84	15	1.05	198	108	57	127	5.2	0
1	68	28.7	102.5	2	1	2	1	0	1	123	79	69	7.3	7.4	7.35	93	14	0.87	144	124	38	91	6	1
2	70	23.7	90.4	1	1	1	1	0	1	141	84	58	8.6	9.3	8.95	90	14	0.59	278	96	57	84	5.9	1
1	63	22.8	84	1	1	2	1	0	0	125	86	63	9.4	9.4	9.4	97	19	0.99	178	73	65	100	5.7	1
1	68	26.5	92	2	2	2	1	1	1	146	88	74	9.3	8.9	9.1	146	18	0.94	157	81	77	81	6.5	0
2	66	21.1	76	1	1	1	0	0	0	114	83	84	9.1	9.3	9.2	104	14	0.74	194	93	46	122	5.6	0

2	67	18.8	72.5	2	1	1	0	0	0	120	79	51	7.4	7.4	7.4	104	19	0.72	185	75	57	110	5.5	1
1	51	22.7	84.5	2	2	1	0	1	1	122	80	56	8.4	8.1	8.25	125	14	1.13	193	72	73	110	6	0
1	73	26.1	90	1	1	1	1	1	1	142	90	62	10.3	9.9	10.1	124	18	0.86	191	98	35	137	7.2	1
2	58	24.7	92	1	1	1	0	0	1	115	73	57	7.7	7.6	7.65	94	14	0.74	212	151	65	120	6	0
1	71	21.0	79.5	2	1	1	1	0	1	138	91	65	8.8	8.8	8.8	94	14	0.97	240	71	52	162	5.5	1
2	50	18.7	70.5	1	1	1	0	0	0	97	61	63	7.2	7.2	7.2	79	15	0.74	189	68	52	118	5.5	0
1	56	28.4	90	2	2	2	1	1	1	141	92	53	8.6	8.5	8.55	169	15	0.91	151	129	68	65	5.4	0
2	59	25.3	86	1	1	2	1	0	1	139	80	52	7.8	8.8	8.3	80	15	0.75	147	43	63	75	5.4	1
1	53	20.7	80	1	1	1	0	0	0	115	71	74	7.6	7.6	7.6	91	16	0.96	155	60	79	76	6	0
1	57	22.0	76	2	1	2	0	0	0	126	78	57	7.1	7	7.05	82	13	1.03	223	67	82	134	5.4	0
2	79	23.5	90.5	1	1	1	1	0	1	142	74	51	8.3	8.2	8.25	104	17	0.91	228	163	61	134	5.9	1
2	48	21.8	82.5	1	1	1	1	0	1	138	91	50	6.9	7.1	7	103	10	0.65	229	103	51	170	5.8	0
2	55	23.3	82	1	1	2	1	0	0	133	91	67	7.3	7.3	7.3	100	12	0.54	200	99	52	134	5.6	1
1	53	21.6	84	2	2	1	0	0	1	122	88	68	9.5	9.3	9.4	123	8	0.52	244	237	82	128	5.6	0
2	56	21.3	87	1	1	2	0	0	0	127	84	75	8.3	8.3	8.3	95	13	0.74	208	95	77	110	5.4	1
1	68	22.6	93	2	1	2	1	0	1	144	93	62	8.8	8.5	8.65	92	16	0.86	162	59	60	130	5.3	0
1	64	24.4	87	2	1	1	0	0	1	129	88	53	8.1	7.8	7.95	116	15	1	192	210	36	123	5.9	1
2	55	22.3	77	1	1	1	0	0	1	126	86	63	6.7	6.4	6.55	90	16	0.63	232	74	94	164	5.6	1
1	44	24.0	86	2	1	1	0	0	0	108	66	62	8	7.8	7.9	108	18	0.82	232	152	42	54	5.8	0
1	84	21.7	87.3	1	2	2	0	1	1	132	80	66	9.5	9.1	9.3	116	18	0.89	96	78	34	91	5.8	1
1	65	23.6	89	2	1	2	1	1	1	132	92	68	8.7	8.4	8.55	131	23	0.72	157	75	55	114	6.2	0
1	38	36.8	113	1	1	1	1	0	1	147	93	59	6.2	5.8	6	105	14	0.75	208	126	44	94	6.4	0
2	49	23.6	86.5	1	1	1	1	0	0	117	81	69	6.8	6.7	6.75	95	11	0.81	184	49	53	151	5.4	1
1	51	23.1	85.7	2	2	2	1	0	1	142	90	61	7.9	8	7.95	96	15	1.08	189	101	91	135	5.4	1
1	49	25.4	95	2	2	2	1	0	0	112	70	53	7.2	7	7.1	123	25	0.98	220	107	72	89	5.9	0
1	52	24.7	85	2	1	1	1	0	1	126	85	59	7.3	7.4	7.35	98	8	0.86	174	187	59	139	5.4	1
2	61	23.1	81	1	1	1	1	0	1	130	88	67	7.1	6.9	7	88	17	0.67	227	121	58	115	5.5	1
1	59	25.8	91.5	1	1	2	0	1	1	115	76	64	8	7.7	7.85	123	14	1.13	190	71	51	74	6.6	1
2	58	21.9	81	1	1	1	1	0	0	165	92	66	8.6	8.7	8.65	85	18	0.78	201	54	66	123	5.4	1

1	64	24.3	95	2	1	1	1	1	1	144	85	85	9.4	9.1	9.25	170	14	0.92	142	65	62	99	7.2	1
2	52	18.7	69	1	1	1	0	0	1	120	74	67	7.2	7.2	7.2	85	21	0.91	185	67	72	119	5.6	1
2	63	19.7	75	1	1	1	1	0	0	115	71	59	7.5	7.6	7.55	75	18	0.56	204	43	80	110	5.6	1
2	52	19.9	77	1	1	1	0	0	0	102	69	65	7.5	7.5	7.5	93	17	0.68	183	60	66	120	5.1	1
1	54	24.5	83.7	2	1	1	0	0	0	111	73	63	6.8	6.9	6.85	101	15	1.08	192	68	55	159	5.7	1
1	50	22.3	76	1	2	1	1	0	1	128	93	66	6.8	6.6	6.7	94	14	1.17	239	76	65	117	5.6	1
1	47	26.5	91	2	1	1	0	0	0	127	85	73	7.3	7.1	7.2	105	15	0.79	191	83	66	103	5	0
2	74	25.5	84	1	1	1	1	0	1	128	79	53	8	8.3	8.15	101	22	0.99	171	109	58	99	5.7	0
1	55	23.6	89	1	2	1	1	0	0	125	90	64	7.3	7.5	7.4	93	12	0.97	174	75	75	136	5	0
1	52	22.4	76	2	1	1	0	0	0	112	77	64	8.8	8.9	8.85	84	14	1.11	205	109	56	122	5.1	1
1	77	25.2	94	2	2	1	1	0	0	123	74	50	9.2	8.7	8.95	109	25	1.14	177	80	41	122	5.2	1
1	74	20.7	85	2	1	1	1	0	0	133	78	62	11.1	10.6	10.85	96	14	1.09	145	57	42	106	5.5	1
2	70	23.8	88	1	1	1	1	0	1	140	88	53	8.8	8.7	8.75	99	16	0.71	252	252	55	170	5.5	1
1	55	23.1	87	2	2	2	0	0	1	124	85	68	8.3	8.4	8.35	106	10	1.06	209	209	70	124	5.5	0
1	60	24.1	92	1	1	1	1	0	0	126	91	58	7.1	6.7	6.9	102	16	1.04	181	181	41	103	5.7	1
1	61	25.7	90	1	1	2	0	0	0	119	87	66	7.9	7.8	7.85	93	24	0.79	156	36	65	89	6	0
1	55	23.7	90.5	2	1	2	1	0	1	143	93	52	6.5	6.3	6.4	101	12	0.91	228	228	54	166	5.6	1
1	58	21.5	79	1	1	1	0	0	1	108	70	39	7.5	7.5	7.5	92	25	0.71	211	211	61	128	6.1	1
1	47	21.4	78	2	2	2	0	0	0	127	89	61	7.4	7.3	7.35	95	17	1.03	148	148	57	74	5.3	1
1	48	27.9	93	2	1	1	1	0	1	152	101	72	7.2	7.2	7.2	97	12	0.95	255	255	45	180	5.5	1
2	66	28.4	88	1	1	2	1	1	1	161	87	93	7.8	7.9	7.85	128	15	0.92	288	229	47	197	5.6	0
2	54	24.0	83	2	2	2	0	0	1	132	80	46	6.8	7.1	6.95	95	20	0.75	250	250	68	167	5.6	1
2	48	21.6	81	2	2	1	0	0	1	98	65	58	7.5	7.4	7.45	93	10	0.67	208	208	79	110	5.4	1
1	51	25.0	93	2	1	2	0	0	1	128	86	67	7.8	7.7	7.75	99	9	0.9	185	185	32	73	5.6	1
1	48	28.1	96.5	2	2	1	1	0	1	118	77	49	7.5	7.8	7.65	107	16	1.04	155	155	41	95	5.5	1
1	56	24.2	80	1	2	1	0	0	0	127	86	60	6.2	6.4	6.3	94	17	1.08	174	174	60	104	5.1	1
1	48	23.2	88	1	1	1	0	0	1	129	83	60	6.3	6.1	6.2	89	20	0.91	202	202	38	138	5.7	1
1	60	23.8	87	1	1	1	1	0	0	127	95	62	9.4	8.4	8.9	104	12	0.95	156	156	67	83	5.4	1
2	53	21.5	80	1	1	1	0	0	0	139	84	99	8.1	6	7.05	94	12	0.63	174	174	64	105	5.2	0

1	46	25.5	100	2	2	2	1	0	1	156	104	87	6.7	6.3	6.5	103	12	0.74	179	179	43	106	5.9	0
1	53	25.1	86	1	1	1	1	0	1	133	91	58	7.1	7.3	7.2	104	10	0.8	246	246	45	160	5.5	1
2	53	21.2	77	1	1	1	1	1	1	136	96	56	7.8	7.5	7.65	122	14	0.65	168	168	48	103	7.1	0
1	53	21.3	79.5	1	1	2	0	0	1	107	76	58	6.8	6.7	6.75	106	15	0.9	212	212	49	144	5.7	0
2	64	20.5	74.5	1	1	1	0	0	1	117	79	65	7.4	7.7	7.55	84	13	0.9	214	214	57	135	5.4	1
1	58	29.6	105	1	1	1	1	0	1	134	92	73	7.2	7	7.1	108	16	0.9	195	195	45	122	6.1	1
1	48	28.3	98.2	2	1	2	1	0	0	119	83	68	6.1	6.1	6.1	109	11	0.87	189	189	58	105	5.9	1
1	51	27.6	96	2	1	1	1	1	1	144	96	65	10	9.4	9.7	142	9	0.93	211	211	67	131	6.2	0
2	74	22.5	86.8	1	1	1	1	0	0	117	74	65	8.9	8.9	8.9	92	17	0.68	188	188	49	114	5.7	0
1	48	25.8	91.5	1	1	1	1	0	1	131	93	65	8	8.2	8.1	102	16	0.83	218	218	51	145	5.7	1
1	52	24.9	86.5	1	1	1	1	0	1	130	90	81	7.5	7.5	7.5	93	17	0.96	157	157	60	90	5.4	1
2	59	20.3	67	1	1	1	0	0	1	105	74	56	7.5	7.6	7.55	87	14	0.65	267	267	86	173	5.8	0
2	73	25.2	98	1	1	1	0	0	1	120	87	54	7.2	7.1	7.15	87	16	0.74	215	215	36	140	5	0
1	47	22.1	80.5	2	2	1	0	0	1	130	88	59	8.3	7.9	8.1	96	17	1.11	211	211	54	118	5.7	0
1	48	25.8	89	2	1	1	0	0	0	123	83	50	6	6.2	6.1	101	17	0.89	189	189	82	109	5.5	1
1	47	24.2	86.5	1	2	1	1	0	1	125	87	67	7.9	7.5	7.7	102	15	0.82	201	201	43	126	5.5	1
1	62	22.4	95.5	1	2	2	1	0	0	124	85	79	9.9	10	9.95	108	21	0.78	151	151	51	66	6	1
1	48	24.4	90.5	2	1	1	0	0	1	129	81	58	6.9	6.8	6.85	103	16	1.11	202	202	44	136	5.8	1
1	57	22.9	84	1	1	1	1	0	1	135	90	86	8.2	7.2	7.7	112	15	0.7	136	136	50	73	5.5	1
1	80	22.0	83	1	1	1	1	0	0	131	64	50	9.6	9.8	9.7	114	23	0.79	132	132	52	75	5.9	1
2	73	22.4	84	1	1	1	1	0	1	131	76	50	9.6	9.7	9.65	84	21	1.01	220	220	84	120	5.7	1
2	63	23.9	83	1	1	1	1	1	1	129	89	69	8.6	8.3	8.45	124	18	0.95	183	183	50	101	6.1	0
2	65	22.8	80	1	1	2	1	0	0	147	92	59	8	7.7	7.85	97	15	0.91	209	46	57	138	5.8	0
1	47	20.3	77	1	1	1	0	0	1	104	69	59	7.4	7.3	7.35	80	11	0.77	228	228	53	131	5.4	0
1	53	21.7	84	1	1	2	1	0	0	127	93	64	8.5	8.4	8.45	100	15	0.88	148	148	40	93	5.3	1
1	66	25.3	93	1	2	1	1	1	1	121	81	67	9.1	8.9	9	113	20	0.87	209	209	57	118	6.8	1
1	53	26.0	90.8	2	1	1	0	1	0	115	77	56	7.9	8	7.95	137	11	0.98	177	177	44	119	6.7	1
1	51	23.5	84	2	1	2	0	0	0	126	86	60	8.3	8.3	8.3	102	18	0.81	183	183	46	123	5.4	1
1	54	25.2	93	2	1	1	1	1	0	133	78	63	7	7	7	154	19	0.99	147	147	55	83	7.1	1

2	71	24.0	86	1	1	1	1	0	1	144	88	79	9.1	8.9	9	93	12	0.54	233	233	48	148	5.6	0
2	57	21.1	83	1	1	1	1	0	1	151	83	55	7.2	7.2	7.2	80	16	0.9	231	231	77	152	5.1	1
2	51	26.7	91.5	1	1	1	0	0	0	116	75	67	6	6.2	6.1	92	16	0.8	183	183	66	107	5.5	1
2	60	20.3	72	1	1	2	0	0	0	113	66	53	7.6	7.7	7.65	94	14	0.63	188	188	63	109	5.4	1
1	52	29.4	96	2	1	2	1	0	0	119	88	72	6.1	5.7	5.9	101	18	0.9	173	173	50	108	5.6	0
2	52	21.0	79	1	1	1	0	0	1	125	82	71	6.9	7	6.95	118	13	0.63	284	101	71	201	5.9	1
1	51	27.4	92	2	1	1	0	0	0	118	77	56	7.7	7.9	7.8	100	18	0.95	145	145	40	85	5.4	0
1	49	26.4	91	2	2	1	0	0	1	123	80	78	7.4	7.3	7.35	80	12	0.91	250	104	45	112	5.3	0
1	64	32.9	104.5	2	1	1	1	0	1	106	66	47	6.7	6.9	6.8	103	15	0.96	113	113	51	59	5.2	0
1	58	25.4	88	2	2	1	1	0	1	142	92	69	9.1	8.7	8.9	100	10	0.77	160	160	57	89	5.9	1
1	50	29.4	104	2	1	1	1	0	1	124	92	58	8	7.4	7.7	111	13	0.82	255	255	33	86	5.4	1
1	57	26.8	93	1	1	2	1	1	1	135	87	49	7.6	7.6	7.6	101	20	1.11	138	138	35	78	5.6	1
1	59	27.0	89.3	1	1	2	0	1	1	133	87	53	7.5	7.3	7.4	127	16	0.86	192	97	48	129	6.8	0
1	48	22.8	87	1	2	1	0	0	1	131	87	57	7.3	8	7.65	121	16	1.26	170	190	39	104	5.4	0
1	48	26.0	91	2	1	2	0	0	0	127	79	58	7.4	7.3	7.35	85	14	0.94	210	101	98	99	5.1	1
1	47	22.9	87.6	2	1	2	0	0	1	114	68	66	8.1	7.9	8	101	17	1.1	258	142	69	174	5.6	1
1	74	22.1	80	2	2	1	1	1	0	144	81	52	9.6	9.5	9.55	147	17	0.96	172	63	59	105	7.7	1
1	68	30.5	104	2	1	1	1	1	1	146	79	84	13.5	11.9	12.7	189	12	1.01	125	133	38	74	9.5	1
1	57	26.4	88.5	2	2	2	0	0	0	136	89	63	7.3	7.2	7.25	111	20	0.74	179	139	42	117	5.6	0
1	52	22.6	89	2	1	1	0	0	0	124	89	71	6.9	6.9	6.9	91	14	0.9	163	100	52	90	5.4	0
1	66	27.3	93.5	2	2	2	1	0	1	135	95	61	9.9	9.3	9.6	101	18	0.9	158	293	57	67	5.5	1
2	64	19.4	74	1	1	1	0	0	0	108	73	60	7.6	7.8	7.7	95	18	0.6	170	74	54	98	5.4	0
1	52	23.8	86.2	2	1	1	1	0	0	138	88	49	8.3	8.1	8.2	113	21	0.96	180	80	48	128	6	1
2	63	20.3	72	1	1	1	1	0	0	138	90	74	7.8	7.7	7.75	96	17	0.55	206	58	62	129	5.2	1
1	60	26.6	99	2	2	1	1	1	1	130	91	64	7.2	7.2	7.2	112	16	0.96	133	81	45	77	6.4	0
1	65	26.7	88	2	1	1	1	0	1	127	90	80	7.6	7.2	7.4	106	13	0.83	172	60	58	111	5.9	0
1	56	26.1	94	2	2	2	1	0	0	139	93	64	7.7	7.2	7.45	100	13	0.89	143	93	41	92	5.4	1
1	47	23.0	89	2	2	1	1	0	0	142	95	54	8.1	8.2	8.15	88	16	0.95	187	51	61	113	5.4	1
2	57	22.2	82	1	1	1	1	0	0	147	90	57	7.4	7.4	7.4	85	14	0.81	220	88	54	158	5.2	1

2	53	21.8	84.2	1	1	1	1	0	1	159	104	60	8.7	8.2	8.45	103	15	0.62	288	207	43	209	5.9	1
1	67	23.1	85.5	2	2	1	1	1	1	116	81	70	9.4	8.6	9	130	12	0.64	181	60	69	105	6.5	1
1	63	17.3	67	1	1	2	0	0	0	114	81	74	9.5	9.1	9.3	99	18	0.92	159	47	77	86	5.4	1
2	52	20.0	76	1	1	1	1	0	0	134	97	59	9.8	11.1	10.45	88	16	0.66	129	150	49	58	5.7	1
1	53	21.1	85	1	1	1	0	1	1	127	86	69	7.2	7.6	7.4	148	12	0.88	142	130	35	84	7.4	0
2	48	19.5	74	1	1	2	0	0	0	128	83	56	7.2	7.2	7.2	98	14	0.87	204	53	95	103	5.8	0
1	50	27.3	94	2	2	2	1	0	0	120	86	63	6.7	7	6.85	96	19	1.28	194	136	67	105	5.4	0
2	52	22.2	80.5	1	1	1	1	0	1	130	86	64	8.3	7.5	7.9	87	8	0.7	145	90	65	67	5.2	1
1	53	25.7	89	2	1	1	1	0	0	111	70	56	7.6	7.9	7.75	87	11	1.01	171	94	49	104	5	0
1	52	22.4	79	1	1	1	0	0	1	110	73	55	7.5	7.3	7.4	98	12	0.98	114	59	31	80	5.4	1
1	60	29.7	99	2	2	1	1	1	1	134	86	64	9.8	9.6	9.7	123	22	2.1	127	207	41	52	7	1
2	52	21.0	75.8	1	1	1	0	0	0	115	67	91	6.9	7	6.95	98	18	0.76	215	56	61	152	5.6	0
2	51	20.6	80	1	1	1	0	0	0	118	76	68	6.7	6.5	6.6	102	8	0.58	205	64	46	148	5.6	1
1	46	26.0	97	2	1	1	1	0	1	139	95	54	7.7	7.7	7.7	106	12	0.91	155	142	27	97	5.4	1
2	52	19.7	78	1	1	2	0	0	0	126	81	53	8.2	8.1	8.15	94	13	0.72	227	52	76	129	5.8	1
1	59	23.9	84	2	2	2	0	1	1	125	81	61	7.3	7.6	7.45	116	15	0.69	190	54	81	106	5.7	1
2	68	20.4	74	1	1	1	0	0	0	114	73	58	9.3	9	9.15	90	16	0.81	168	116	73	117	5.4	1
2	55	21.6	75.5	1	1	1	0	0	1	108	64	50	6.7	6.9	6.8	86	16	0.69	233	59	90	130	5.5	1
1	63	21.1	82	1	1	1	0	0	0	119	84	57	9	8.9	8.95	109	19	0.98	203	101	52	128	5.3	1
2	48	21.6	70.2	1	1	1	0	0	0	127	78	87	6.3	6.5	6.4	104	14	0.71	201	146	69	108	5.6	0
1	73	21.9	80	2	1	2	0	0	1	124	79	56	8.4	8.3	8.35	98	16	1.06	216	43	71	123	6.3	1
1	71	23.3	85	1	1	1	0	0	0	131	73	59	8.6	8.7	8.65	82	16	0.89	192	38	56	140	5.2	0
1	59	22.9	91	1	1	1	0	1	1	118	75	64	8.4	8.3	8.35	111	18	0.98	114	73	34	62	7	1
1	61	24.8	88	2	1	2	0	0	0	124	82	58	6.8	6.8	6.8	91	21	0.89	224	178	52	137	5.4	1
1	65	27.4	98	2	1	1	1	0	0	117	80	55	7.5	7.4	7.45	95	12	0.81	159	159	60	91	5.4	0
1	64	25.6	97	2	2	1	1	0	0	126	93	60	6.1	6.1	6.1	112	18	0.71	182	182	64	107	6	1
1	66	23.5	85	2	1	1	1	0	1	122	78	58	9.5	9.1	9.3	106	25	1	168	168	53	91	5.4	1
1	57	22.8	83	2	1	1	1	0	1	145	109	86	9.6	9.7	9.65	100	11	1.08	163	163	38	109	5.9	0
2	66	21.6	77	1	1	2	0	0	1	127	76	68	9.1	9	9.05	92	15	0.71	222	222	76	127	5.7	1

2	60	23.7	87	1	1	2	0	0	1	104	73	57	9	8.9	8.95	101	18	0.76	229	229	67	150	5.9	1
1	48	25.9	96.5	2	1	1	1	1	1	114	86	80	8	7.8	7.9	126	13	1.21	189	189	39	122	6.5	0
1	74	26.6	91	2	2	1	1	0	1	149	92	48	8.2	7.5	7.85	115	16	0.84	179	179	57	92	5.6	0
1	53	23.4	83	2	1	1	1	0	1	118	87	71	7.5	7.3	7.4	111	11	1.05	148	148	41	93	5.5	0
1	51	22.5	85	2	1	1	1	0	0	150	88	57	7.5	7.5	7.5	106	16	0.8	174	113	44	113	5.7	0
1	67	25.0	96	2	1	1	1	0	1	140	85	74	9.9	10	9.95	103	18	0.95	140	140	50	71	5.7	1
1	65	26.4	96	2	1	2	1	1	1	140	86	56	7.9	7.9	7.9	165	17	0.78	165	94	67	88	7.3	1
1	51	26.1	94	2	1	2	1	0	1	122	97	66	8.7	8.3	8.5	107	19	1.01	236	236	36	129	5.6	1
2	49	22.8	82	2	1	2	1	0	1	159	102	68	7.1	6.8	6.95	125	13	0.63	163	293	35	69	5.4	1
1	48	19.0	80	2	1	1	1	0	1	104	63	56	9	9.3	9.15	89	18	0.93	224	102	46	166	5.4	1
1	75	22.9	89	2	1	1	1	1	1	124	79	63	12.1	10.9	11.5	123	26	0.97	147	47	62	84	6.5	1
1	79	21.4	87	1	1	2	1	1	1	145	89	93	10.3	10.5	10.4	157	15	0.86	108	121	51	42	7.2	0
1	54	23.5	80.5	2	2	1	1	0	1	123	94	62	8	7.4	7.7	121	15	0.96	226	96	62	149	6.3	1
1	55	24.4	89	1	1	1	0	1	1	116	86	70	6.8	7	6.9	112	22	1.29	306	96	51	240	6.9	1
1	73	21.9	82	2	1	2	1	1	1	117	63	73	9.9	9.8	9.85	129	17	1.01	180	107	61	107	7.1	1
1	59	24.5	86	2	1	1	1	0	1	129	99	58	6.8	6.6	6.7	103	23	1.18	168	75	53	108	5.8	0
1	38	25.3	89	2	1	2	1	0	0	120	78	63	6	5.9	5.95	97	15	0.79	163	52	56	106	5	1
1	50	29.4	106	2	1	2	1	0	1	132	83	56	7.1	7.2	7.15	89	10	0.75	151	144	49	88	5.9	1
2	51	21.8	75	1	1	1	0	0	0	116	75	71	6.9	7.2	7.05	98	10	0.66	216	38	66	155	5.8	1
2	50	27.0	88	1	1	2	0	0	1	135	79	65	6.4	6.2	6.3	103	15	0.68	207	261	53	111	5.5	0
2	66	24.3	81	1	1	1	1	0	1	139	90	66	9.3	8.7	9	87	13	0.66	170	118	61	98	6	1
1	58	27.0	90.5	2	2	2	0	0	1	122	89	73	6.9	6.3	6.6	91	21	0.91	211	99	61	128	5.1	0
2	47	22.0	72	1	1	1	0	0	1	100	68	72	6.8	6.9	6.85	88	14	0.62	261	46	63	178	5.4	0
1	73	27.3	94	2	2	2	1	1	0	135	85	66	9.8	10.2	10	133	13	0.76	201	105	60	129	6.5	1
2	54	18.8	76	1	1	2	0	0	0	126	87	45	8.1	8	8.05	88	14	0.68	203	79	58	127	5.3	1
2	63	21.8	88.5	1	1	2	0	0	1	110	68	59	8.3	8	8.15	103	14	0.56	175	139	64	94	5.8	1
1	54	20.3	77	1	1	2	1	0	0	121	84	68	8.8	9.1	8.95	113	10	0.78	212	130	49	134	5.9	0
1	60	23.5	79.6	2	1	2	1	0	1	129	85	63	8	6.9	7.45	94	12	1.11	209	53	62	88	5.4	1
2	61	23.1	91.5	1	1	1	0	0	1	137	79	57	8.7	8.4	8.55	101	12	0.62	184	99	69	102	5.8	1

1	64	24.0	88	1	1	1	1	1	1	140	88	51	8.6	8.9	8.75	139	13	0.78	179	92	50	113	6.9	1
2	69	24.1	84	1	1	1	1	0	1	132	78	68	10.8	11	10.9	105	12	0.76	192	65	64	120	6.3	1
1	49	24.7	82.8	1	2	1	0	0	0	136	85	71	7.5	7.4	7.45	99	8	0.84	155	63	70	76	5.1	1
1	45	23.1	86.5	2	1	2	0	0	1	130	80	68	7.3	7.1	7.2	107	13	0.81	195	133	35	142	5.3	0
1	66	21.7	82	1	1	1	0	0	1	123	86	57	8.1	8.1	8.1	89	17	0.97	133	64	45	82	5.7	1
2	71	23.2	83	1	1	1	1	0	1	140	75	73	9.7	9.5	9.6	94	10	0.49	191	212	48	114	5.7	1
1	52	23.4	90	1	2	1	1	0	0	120	85	73	7.5	7	7.25	88	15	0.93	191	79	56	127	5.6	0
1	49	22.7	83	1	1	2	1	0	1	136	94	81	7.3	7.5	7.4	110	11	1.02	254	42	57	179	5.7	1
2	60	21.4	79	1	1	1	0	0	1	124	79	62	8.4	8.9	8.65	89	14	0.69	249	145	57	153	5.7	1
1	57	21.5	81	2	1	1	1	0	0	120	78	49	7.9	8.1	8	96	12	0.82	165	40	55	103	5.4	1
1	48	23.6	89	2	1	1	0	0	0	113	63	47	8.2	8	8.1	93	11	0.98	203	51	58	127	5.4	0
1	47	26.5	92.9	2	1	1	1	0	1	127	83	68	8	7.8	7.9	116	15	0.96	239	142	43	173	5.4	1
2	77	22.4	83	1	1	1	1	0	1	144	86	62	9	8.8	8.9	90	19	0.9	201	122	96	89	5.6	0
1	66	28.0	102	2	2	2	1	0	1	148	92	56	8	8.1	8.05	115	14	0.8	162	54	34	117	6.1	0
1	56	23.6	80	2	1	1	0	1	0	134	86	68	10.7	9.7	10.2	246	20	0.98	178	76	102	68	7.3	0
1	52	24.9	91	2	1	1	0	1	1	118	81	60	7.7	7.7	7.7	117	18	0.79	159	94	46	96	6.5	0
1	75	22.9	88	1	1	2	1	0	1	133	79	51	8.5	8.6	8.55	91	25	0.93	172	89	59	102	5.4	1
2	64	29.1	99	1	1	1	0	0	1	125	79	60	7.3	7.5	7.4	85	11	0.56	126	86	42	75	5.7	1
1	58	25.2	87.5	1	2	1	1	0	0	122	86	62	7.3	7.4	7.35	112	16	1.08	161	86	55	98	5.6	0
1	57	22.9	91	1	1	1	0	0	0	116	79	59	8	7.9	7.95	99	13	0.95	174	88	41	112	5.7	1
1	58	26.5	93	2	2	2	1	0	1	133	82	47	7.9	7.8	7.85	112	16	1.01	197	153	38	129	6.1	1
1	66	23.5	104.7	2	1	2	1	0	1	147	96	90	8.7	9	8.85	113	15	0.95	161	76	48	109	5.9	1
2	63	21.2	76	1	1	1	1	0	0	107	67	51	9.8	9.8	9.8	91	14	0.7	188	69	60	117	5.4	1
1	63	26.3	90	2	2	1	1	0	0	137	90	59	8	7.9	7.95	98	16	1.03	178	171	43	113	5.6	1
2	58	19.6	72	1	1	1	1	0	0	125	84	64	8.5	8.4	8.45	87	11	0.62	178	85	63	102	5.9	1
1	56	24.8	84.5	2	1	1	1	0	0	112	81	65	7	6.9	6.95	90	19	0.93	186	71	56	113	5.5	1
1	53	24.8	90	2	1	2	0	0	1	119	77	59	8	7.6	7.8	100	13	1.05	245	123	58	160	5.4	1
1	61	25.4	96	1	2	1	1	0	1	141	90	58	9.3	9	9.15	102	18	0.94	226	72	76	139	5.8	1
1	58	27.5	101	2	2	1	1	0	0	112	73	52	8.1	8.2	8.15	100	23	1.05	103	58	41	54	5.9	0

1	60	28.0	94	1	2	1	1	0	1	155	97	54	9.2	8.4	8.8	96	21	0.96	188	205	36	126	5.8	1
1	45	23.0	87	2	1	1	0	0	0	130	85	79	6.7	6.4	6.55	99	15	0.95	218	123	69	129	5.2	0
1	67	23.3	91.5	2	1	1	1	1	1	185	108	71	10	10.1	10.05	134	18	1.14	134	89	40	94	6.2	1
1	74	23.6	88	2	1	1	0	1	1	119	73	66	9.3	9.1	9.2	178	14	0.92	167	143	49	92	6.3	1
2	56	23.2	82.2	2	2	1	0	0	0	116	75	69	7	7.1	7.05	102	18	0.71	179	90	45	118	5.5	1
1	56	24.8	90	2	2	2	1	0	1	128	87	68	8.8	8.1	8.45	103	13	0.87	184	69	52	126	5.6	0
1	47	22.6	80	2	1	2	0	0	0	120	83	61	7.8	8.2	8	97	10	0.87	196	149	49	121	5.9	1
1	73	23.0	88	2	2	1	1	0	1	117	78	73	8.7	8.6	8.65	104	10	0.98	136	109	43	83	5.8	1
1	36	25.2	96.5	2	1	1	0	0	0	130	87	63	6.5	6.6	6.55	99	11	0.66	163	84	48	114	5.3	1
1	58	18.1	75	2	2	2	0	0	0	116	76	76	8.5	8.3	8.4	78	14	0.74	231	47	85	138	5.2	1
1	55	24.7	82	2	1	2	1	0	1	128	91	61	7.2	7.4	7.3	85	12	1.07	142	139	38	90	5.2	1
2	71	25.1	88	1	1	1	1	1	1	133	80	63	9.1	9.1	9.1	103	13	0.69	158	128	57	81	6	1
1	58	25.6	91	2	2	1	1	0	0	139	92	56	7.2	7.1	7.15	100	19	0.92	178	58	78	100	5.4	1
1	67	21.6	87	2	1	2	1	0	1	189	117	80	10.7	10.3	10.5	119	16	1.01	179	167	55	98	5.8	0
2	58	20.7	81	1	1	2	1	0	0	108	73	63	8.4	8.2	8.3	108	15	0.62	176	66	66	108	5.3	0
2	58	23.6	87	1	1	1	1	0	0	130	73	72	7.5	7.6	7.55	100	16	0.75	174	111	52	100	5.6	0
1	51	19.3	70	1	1	2	0	0	0	110	67	57	7.4	7.4	7.4	83	10	1.04	182	72	61	108	5.3	1
1	51	25.1	92	2	1	2	1	0	1	118	81	56	6.9	6.7	6.8	124	14	0.79	175	87	63	107	5.9	0
1	31	24.0	89.5	2	2	2	0	0	0	116	74	60	6.4	6.2	6.3	90	12	0.8	187	90	70	115	5.4	0
2	55	22.5	79	1	1	2	1	0	0	155	98	54	7.2	7.3	7.25	100	13	0.58	231	57	90	128	5.2	0
1	62	25.7	92	2	1	2	0	0	0	118	79	52	7.8	7.5	7.65	89	17	0.94	168	107	44	113	5.5	1
1	72	21.5	88	2	1	1	1	0	1	101	66	64	7.6	7.9	7.75	89	16	1.05	208	260	34	124	5.9	1
2	70	21.0	75.5	1	1	1	1	0	1	143	89	62	8.1	8.2	8.15	118	24	0.7	157	143	51	92	6.1	0
2	66	30.5	105	1	1	1	1	1	1	125	79	71	7.3	7.1	7.2	103	12	0.66	137	138	49	56	6.5	0
1	68	22.5	86	1	1	2	1	0	0	133	85	64	7	7.4	7.2	109	16	1.03	202	74	71	121	5.4	0
2	62	20.8	79	1	1	1	0	0	1	126	87	57	9.2	8.3	8.75	109	11	0.61	245	131	48	177	6	1
1	61	24.6	92	2	2	2	0	0	0	118	85	56	9.1	9.3	9.2	99	13	0.85	184	88	59	110	5.5	1
1	69	21.5	78	2	1	2	1	0	0	137	94	66	9.9	9.4	9.65	93	25	0.98	134	124	40	71	5.8	1
1	59	25.5	85	1	1	1	1	0	0	158	114	87	8.7	7.7	8.2	106	17	1.03	188	104	46	129	5.6	0

1	55	28.1	92	1	1	1	0	0	1	129	83	58	7.7	7.5	7.6	100	14	1.01	258	85	77	164	5.6	1
1	49	23.2	83	2	2	2	0	0	0	120	82	72	8.2	8.2	8.2	91	16	1.04	197	130	46	125	5.4	1
1	58	24.4	92.8	1	2	2	1	0	0	130	93	74	8.6	9	8.8	98	14	0.87	225	94	47	156	5.5	0
2	76	23.0	79.5	1	1	1	0	0	0	131	84	56	10	9.4	9.7	93	31	0.6	228	45	83	140	5.6	1
1	54	23.1	86	2	1	1	0	0	0	125	87	63	8.6	8.4	8.5	111	13	0.89	215	112	61	127	5.6	1
1	56	23.5	83	1	1	1	0	0	1	108	76	65	7.8	7.5	7.65	98	17	0.99	182	251	31	102	5.5	0
1	56	24.9	84	2	2	2	0	0	1	117	80	60	8.1	7.9	8	104	14	1	180	411	32	87	5.5	1
1	66	23.0	84	1	1	2	1	0	1	142	93	62	9	9	9	108	13	1.22	223	68	60	162	6.4	1
2	53	22.3	79	1	1	2	0	0	0	107	73	74	7.2	7.1	7.15	87	15	0.8	233	57	55	159	5.9	1
1	67	24.9	91	2	1	2	1	0	1	141	86	53	8.3	8.3	8.3	95	14	1.1	126	70	50	76	5.6	1
2	43	20.7	81	1	1	2	0	0	0	116	76	62	6.2	6.2	6.2	91	9	0.65	198	56	66	119	5.3	1
1	60	22.7	91	1	1	2	1	0	1	153	102	78	10	10.1	10.05	101	13	0.84	144	60	65	66	5.5	1
1	55	23.4	85	1	1	2	0	0	0	114	78	67	8.4	7.9	8.15	98	17	1.04	202	66	77	115	5.4	1
2	66	21.0	74	1	1	2	0	0	0	113	71	61	8.8	8.7	8.75	93	15	0.66	212	69	75	122	5.4	0
1	55	23.4	88.8	2	1	2	0	0	0	98	61	64	7.9	8	7.95	98	16	1.29	221	116	48	143	5.4	1
1	68	27.4	100	1	1	1	1	0	1	137	86	63	9.8	9	9.4	102	18	0.78	119	77	44	71	5.9	1
1	50	26.2	88.8	1	1	2	0	0	1	116	78	60	7.3	7.3	7.3	99	10	0.98	212	98	61	125	5.4	1
1	51	24.6	86	2	1	2	0	0	1	129	84	64	7.5	7.4	7.45	102	13	0.94	176	100	64	94	6.1	1
1	59	28.4	96.5	2	1	2	0	0	1	129	79	49	8.2	8.1	8.15	122	17	0.81	230	138	42	168	6.4	1
1	54	24.1	87.5	2	1	2	0	0	1	120	79	60	8.1	7.9	8	91	13	1.07	190	215	41	122	5.8	1
1	56	22.9	82	2	1	1	0	0	0	130	82	57	7.9	7.7	7.8	118	16	0.81	167	59	49	103	5.8	1
1	48	25.3	88	2	1	2	0	0	0	103	71	53	8	7.8	7.9	88	12	0.75	167	142	51	91	5.9	1
1	46	29.7	96.5	1	1	1	1	1	1	162	103	90	9.2	11.5	10.35	201	19	1.46	199	314	39	110	8.6	1
1	41	25.6	84	2	2	2	1	0	1	148	92	74	7.2	6.9	7.05	97	14	1.14	251	304	50	159	5.4	0
1	50	22.9	86.5	2	1	2	0	0	0	115	82	64	9.3	9.2	9.25	98	16	0.86	234	118	50	158	5.5	0
1	47	23.9	81.5	2	2	1	0	0	1	120	81	62	7.4	7.2	7.3	110	16	0.93	224	249	60	126	5.8	1
1	51	22.7	81.5	2	1	1	1	0	0	140	95	66	7.1	6.9	7	87	12	0.96	228	149	48	153	5.7	1
1	60	23.4	79	2	2	2	1	0	0	130	88	65	7.7	8	7.85	108	13	0.88	160	80	54	103	5.8	0
1	58	24.7	95	2	1	2	0	0	0	116	80	64	7	7	7	101	11	0.8	207	164	58	134	6	1

1	54	21.4	86.5	2	1	1	0	0	0	100	72	59	7.1	7.1	7.1	98	10	0.87	142	64	75	67	5.4	1
2	66	22.3	74	1	1	1	0	0	0	107	73	55	7.2	7.2	7.2	87	16	0.67	198	63	71	115	5.4	1
1	59	26.1	91.5	2	2	2	1	0	1	110	81	61	7.2	6.9	7.05	100	18	0.83	159	169	39	103	5.1	0
1	77	27.1	91	2	1	2	1	0	1	155	80	58	9.6	9.6	9.6	118	15	1.04	126	103	46	69	5.9	1
1	49	22.8	78.2	2	1	1	0	0	0	128	88	57	6.8	6.9	6.85	94	16	1.07	234	55	69	153	5.3	0
1	58	24.9	85	2	1	2	1	0	0	130	91	63	7.6	7.6	7.6	103	11	0.99	103	108	55	136	5.6	1
1	51	25.3	93	2	2	1	0	0	0	131	86	54	8	8.1	8.05	99	10	0.84	195	106	52	119	5.5	0
1	60	17.0	64.5	1	1	1	1	0	0	132	90	118	8.7	9	8.85	118	14	1.08	172	66	84	89	5.8	0
1	58	23.6	88.5	2	2	2	1	0	0	109	76	71	6.6	6.6	6.6	96	13	0.84	201	73	68	123	5	0
1	43	26.9	98	1	1	1	0	0	1	116	84	62	6.9	6.7	6.8	94	16	1.15	206	136	55	143	5	0
1	50	22.4	78	1	1	1	0	0	0	117	81	64	7.9	7.9	7.9	98	12	0.69	176	68	63	102	5.1	1
1	58	23.4	86.5	2	2	2	0	0	1	128	79	62	10.1	9.1	9.6	109	17	1.11	141	122	57	80	6.4	1
1	53	22.2	86	2	2	2	1	0	1	130	82	94	9.4	8.6	9	109	10	0.78	155	272	50	81	6	0
1	60	23.7	95.5	2	2	2	0	0	0	129	83	63	6.8	7	6.9	106	11	1.07	235	169	60	152	5.7	0
1	53	22.5	86	1	1	1	0	0	1	126	88	72	7.6	7.6	7.6	99	12	0.93	201	65	60	130	5.8	1
1	41	24.9	88	2	2	2	1	1	1	120	88	58	6.9	7.1	7	171	15	1.35	199	254	36	116	6.5	1
1	72	24.8	86	1	1	1	1	1	1	150	85	42	9.3	9.3	9.3	136	12	0.82	124	102	45	64	6.8	1
1	75	25.1	91.5	1	2	2	1	0	0	131	81	64	9.4	9.3	9.35	107	21	1.06	211	73	69	137	5.4	0
1	62	24.2	86	2	2	2	1	0	1	132	84	73	9.2	9.2	9.2	105	16	0.86	175	79	110	65	5.6	1
2	59	20.8	78	1	1	1	0	0	1	124	79	55	8.2	8.3	8.25	99	24	0.67	240	55	60	164	5.5	0
1	58	27.2	96	2	1	1	1	1	0	142	99	61	8.3	8.1	8.2	128	13	1.08	187	115	67	104	6.5	1
1	51	21.5	80.6	2	1	2	0	0	0	99	64	49	8.1	8	8.05	77	19	1.07	183	137	44	119	5.5	1
1	45	22.8	85	1	1	1	1	0	1	139	91	81	7.3	7.5	7.4	108	14	0.91	223	116	48	159	5.4	1
1	66	21.8	86	1	1	2	1	0	0	130	93	73	8.7	8.9	8.8	99	15	0.89	190	102	45	132	5.5	0
1	52	22.7	82	2	2	2	0	1	0	113	68	54	8.2	8.1	8.15	141	15	0.84	172	56	76	88	6.7	0
1	63	24.6	89	2	1	2	1	1	0	115	71	53	8.7	8.9	8.8	115	14	0.82	196	147	45	130	6.8	0
1	57	26.1	92	2	2	2	1	0	1	142	90	56	8.3	8.1	8.2	109	14	1	166	81	64	86	5.5	1
2	51	21.6	77	1	1	1	1	0	1	139	102	80	7.6	7.9	7.75	95	14	0.68	218	85	62	143	5.6	1
1	63	24.9	80.5	1	1	2	1	0	0	146	99	69	8.4	8.1	8.25	97	15	0.9	179	82	49	115	5.4	1

1	60	25.5	95	2	2	2	1	0	1	156	87	65	8.1	8.1	8.1	113	14	0.93	150	112	46	95	5.9	1
1	45	23.9	87	1	2	1	1	0	0	127	92	73	11.3	12.9	12.1	104	11	0.98	181	86	40	125	5.4	1
1	55	23.4	83.5	2	1	2	0	0	1	118	81	58	7.7	7.9	7.8	101	11	0.68	230	511	36	130	5.8	1
1	59	24.5	87	2	1	2	1	0	1	162	109	76	10.2	9.5	9.85	103	13	0.89	193	227	38	110	5.8	0
1	63	25.1	91	2	1	2	1	0	1	145	93	52	8.2	8.6	8.4	102	16	1.03	217	99	75	125	5.4	1
1	56	27.8	96	1	1	1	0	0	0	131	85	67	6.8	6.8	6.8	125	15	0.89	220	88	57	146	6.1	1
1	45	23.3	84	2	2	2	0	0	0	130	85	54	7.2	7.3	7.25	107	15	0.91	212	102	65	129	5.7	0
2	59	21.2	75	1	1	2	0	0	0	122	72	57	8.1	7.8	7.95	92	11	0.68	198	50	72	116	5.4	1
1	45	25.1	95	2	1	2	1	0	0	144	99	74	5.6	5.8	5.7	96	12	0.94	197	168	45	125	5.5	0
1	54	23.3	85	2	1	2	0	1	1	122	89	75	7.3	7.2	7.25	128	19	0.87	192	155	36	133	5.7	0
1	52	26.5	91	2	2	1	1	0	0	125	91	54	7.8	7.7	7.75	97	12	0.99	207	52	60	142	5.4	1
1	58	23.7	87.5	2	1	2	1	0	1	127	78	54	8.1	7.8	7.95	96	17	0.99	154	135	36	100	5.4	0
1	51	21.5	80	2	1	2	1	0	1	123	84	62	7.7	7.8	7.75	104	15	0.84	173	212	44	100	5.6	1
1	55	27.0	97	2	2	1	1	0	0	140	93	71	7.4	7.6	7.5	115	10	0.64	164	52	72	88	5.7	1
1	63	25.6	93.7	2	1	2	0	0	1	128	88	60	8.8	8.4	8.6	107	15	0.85	172	97	59	103	5.4	1
2	59	26.6	90.5	1	1	2	0	0	1	117	73	62	7.3	7.4	7.35	102	15	0.49	246	116	46	180	5.8	1
1	52	24.0	84	1	1	2	1	1	1	134	104	73	8.8	7.8	8.3	137	12	0.9	222	233	45	148	7.1	1
1	48	26.0	97	2	1	2	0	1	1	114	85	70	7.9	7.8	7.85	164	13	0.79	199	159	38	143	9.7	1
1	65	29.0	111	2	1	2	1	0	0	112	78	78	8.5	8.1	8.3	114	18	0.94	202	78	57	136	5.7	1
1	60	28.4	110.5	2	1	1	1	1	1	123	89	72	7.9	7.6	7.75	113	13	0.91	238	133	58	158	5.8	0
1	50	23.2	82.5	1	2	2	1	0	1	126	91	65	6.5	6.7	6.6	106	9	0.64	190	541	35	81	5.7	0
1	50	26.4	97	1	1	2	1	1	1	149	95	72	8.1	7.9	8	140	14	0.76	193	266	41	130	7.1	1
1	50	22.5	75.5	1	1	2	1	0	0	149	98	61	8.9	7.8	8.35	89	17	1.03	235	114	58	135	5.7	1
2	47	22.9	84.2	1	1	2	0	0	0	113	82	65	7.6	7.7	7.65	81	11	0.76	197	133	57	116	5.4	1
1	57	23.6	82.8	2	1	2	0	0	0	107	74	54	8.1	7.7	7.9	98	18	1.06	213	149	47	144	5.7	1
1	56	23.2	84.8	2	1	2	1	0	1	120	78	93	7.1	7.1	7.1	111	16	1.45	198	133	50	138	5.6	0
1	69	23.7	94.1	2	2	1	1	1	1	131	88	62	8	8	8	106	14	0.76	138	90	79	59	5.9	1
1	49	27.0	90.8	1	1	1	1	0	0	126	75	56	7.1	7.3	7.2	101	14	0.94	208	69	57	150	6.2	0
1	60	25.6	91	2	2	2	0	1	1	134	95	60	8.6	8.3	8.45	121	17	1.09	145	112	54	73	7.4	0

2	51	23.1	81	1	1	2	0	0	0	123	70	53	7.1	7	7.05	94	12	0.88	223	62	64	148	5.4	0
1	74	23.2	88	2	1	2	1	0	0	169	100	77	9.2	9.3	9.25	95	17	0.97	182	90	40	114	5.6	1
1	59	24.7	87.5	2	1	1	1	0	0	127	79	52	7.6	7.4	7.5	100	20	1.02	188	47	71	119	5.6	1
2	52	26.5	93.5	1	1	2	1	0	1	144	79	71	6.2	6	6.1	87	13	0.65	260	123	71	159	5.4	0
1	58	24.7	89	2	1	2	0	1	1	126	87	77	9.9	9.6	9.75	141	19	1	163	107	53	106	6.5	0
1	50	25.4	92	2	1	1	0	0	0	107	64	63	6.7	6.4	6.55	91	15	0.7	114	47	47	66	5.1	1
1	62	26.3	95	2	1	2	1	1	1	140	95	61	9.9	10	9.95	118	11	0.6	235	126	44	171	6.5	1
1	61	27.1	95	2	1	2	1	0	1	125	84	64	9.8	9.8	9.8	104	19	1.02	188	109	34	135	5.9	1
1	50	23.7	86	1	2	2	1	0	0	119	75	62	6.5	6.9	6.7	96	10	0.9	161	106	87	70	5.3	0
1	46	27.2	96.5	2	2	2	1	0	0	150	84	46	8.6	8.5	8.55	105	14	1	183	117	49	125	5.6	1
1	58	25.7	89.5	2	1	2	0	0	0	115	75	75	7	6.9	6.95	89	14	1.01	217	92	62	140	5.3	1
1	56	22.7	83	1	1	2	0	0	1	123	81	52	8	7.9	7.95	95	15	0.85	183	66	80	106	5.5	0
2	64	21.2	78	1	1	1	0	0	1	134	81	63	6.9	6.8	6.85	103	16	0.66	196	45	62	135	5.5	1
1	53	24.4	87.5	2	2	2	1	0	0	128	90	64	7.3	7.2	7.25	85	12	0.79	183	145	71	86	5.8	0
1	60	25.3	90	2	1	2	1	0	1	146	93	66	8	7.8	7.9	105	19	0.73	225	122	54	163	5.4	1
1	49	22.5	87.5	2	2	1	1	1	1	133	98	70	7.7	7.9	7.8	133	15	0.93	193	211	56	110	5.5	0
1	51	24.3	87	1	1	1	1	0	1	140	98	66	7.7	8	7.85	103	15	1.01	181	134	76	100	5.8	0
1	56	28.4	96.7	2	1	2	0	0	1	121	84	55	6.6	6.6	6.6	100	20	0.98	241	106	47	171	6	0
1	68	25.2	93	1	1	2	1	0	0	132	88	62	9	9.1	9.05	114	17	0.87	180	122	41	114	5.9	0
1	53	24.7	83	2	1	2	1	0	0	139	97	50	6.3	6.1	6.2	111	10	0.97	211	182	41	138	5.6	0
1	77	22.0	82	2	1	1	1	1	1	147	95	76	11.3	10.1	10.7	137	19	1.05	159	140	35	96	7.4	0
1	73	23.4	88	2	1	2	1	0	1	134	79	77	10.8	10.2	10.5	113	21	1.33	180	220	38	112	5.7	0
1	51	23.3	86	2	2	1	0	0	0	117	87	58	8.1	7.7	7.9	92	18	0.97	179	127	62	96	5.3	0
2	60	27.6	93	1	1	1	0	0	0	133	87	58	7.3	7.2	7.25	97	20	0.97	208	181	44	136	5.7	1
2	51	21.7	73	1	1	2	0	0	0	107	67	53	7.5	7.6	7.55	92	13	0.64	172	73	60	105	5.5	1
1	52	27.1	92	2	2	2	0	1	0	115	77	59	7.7	7.5	7.6	120	14	0.76	189	68	44	141	6.4	1
1	72	25.3	92	2	1	2	1	1	1	139	78	56	9.8	9.5	9.65	147	23	1.29	130	81	60	64	8.1	1
1	55	26.4	98.5	1	1	1	1	0	1	144	92	76	7.7	7.2	7.45	108	15	1.11	195	176	39	132	5.7	1
1	49	24.7	88	2	2	1	0	0	0	137	88	66	7	7	7	94	21	0.98	219	128	55	151	5.4	1

2	44	19.2	71	1	1	1	0	0	0	109	74	61	7.2	7.2	7.2	89	11	0.59	189	103	57	102	5.3	0
1	58	22.7	82	2	1	2	0	0	0	135	81	55	7.3	7	7.15	87	20	0.77	166	83	56	99	5.3	1
1	57	23.3	86.5	2	1	2	0	0	0	130	83	66	7.1	7.4	7.25	106	15	1.02	228	101	63	157	5.4	1
1	52	26.1	89	2	1	2	1	1	1	108	69	69	6.9	6.6	6.75	136	15	0.91	147	124	48	83	6.9	0
2	48	21.4	75	1	1	2	0	0	0	115	74	69	6.7	6.5	6.6	83	14	0.68	212	51	75	127	5.5	0
1	54	27.8	103	2	1	2	1	0	1	140	103	74	7.6	7.5	7.55	97	11	0.85	238	241	40	156	5.6	0
1	67	23.1	84	1	1	2	0	0	0	130	82	62	8.5	8.2	8.35	107	14	0.96	206	137	64	126	5.4	1
1	57	22.9	80	2	1	2	0	0	0	121	82	66	7.5	7.4	7.45	110	18	0.94	182	71	63	105	5.6	1
1	47	21.6	83	2	1	1	1	0	1	124	92	86	7.8	7.6	7.7	96	14	0.86	225	107	46	165	5.5	1
1	52	20.0	71	2	1	2	0	0	0	101	70	60	7.7	7.6	7.65	94	7	0.77	202	109	62	120	5.6	1
1	61	25.6	91	2	2	2	1	1	1	147	98	78	9.3	8.8	9.05	128	14	0.98	186	95	47	127	7	1
1	49	19.0	78	1	1	2	0	0	1	124	79	61	7	7.1	7.05	92	12	0.8	151	146	29	100	5.3	1
2	52	18.4	68	1	1	2	0	0	0	114	72	58	8.8	8.7	8.75	86	12	0.58	163	54	73	81	5.7	0
1	56	25.8	87.5	2	1	2	0	0	1	112	75	59	6.8	6.8	6.8	102	15	0.85	188	210	49	115	5.3	0
1	45	26.4	89	2	2	2	1	0	1	102	75	64	7.4	7.6	7.5	109	19	1.02	279	146	61	208	5.5	0
2	56	22.3	81.5	1	1	1	0	0	1	118	87	63	8	7.6	7.8	97	10	0.61	173	113	50	106	5.8	0
2	49	20.3	77	1	1	2	1	0	0	124	71	69	7.5	7.6	7.55	96	13	0.6	169	80	55	105	5.8	1
1	64	23.5	87	2	1	2	0	1	1	136	85	55	9.2	9	9.1	123	11	0.92	207	106	52	139	6.4	0
1	53	26.0	91	1	1	1	1	0	0	139	101	72	8.4	8.3	8.35	85	11	0.74	214	99	49	151	5.3	1
1	49	28.0	95.6	2	2	2	1	0	1	130	90	66	7.5	7.2	7.35	103	17	1.15	143	102	72	65	5.4	0
1	51	22.4	81.5	1	1	2	0	0	0	137	82	55	7.6	7.7	7.65	96	18	1	177	140	55	105	5.4	0
2	66	21.2	79.8	1	1	2	0	0	1	124	81	63	8.6	8.2	8.4	99	17	0.6	176	179	50	94	5.5	0
1	57	26.5	94	2	1	2	1	0	1	129	89	91	10.3	11.1	10.7	105	13	1.08	202	66	51	143	5.9	1
1	61	26.7	94	2	2	1	1	0	0	129	90	54	9.3	9.2	9.25	101	12	1.01	168	106	54	102	5.7	1
1	58	25.2	95	2	2	1	0	0	1	128	88	55	8.7	8.5	8.6	115	19	0.84	241	133	61	155	6	1
1	58	22.1	84	2	1	2	0	0	1	126	83	60	7.8	7.8	7.8	94	15	0.98	278	137	55	185	6.1	0
1	63	24.6	88.8	2	2	2	1	1	1	165	100	78	8.8	9	8.9	101	14	1.11	156	192	31	89	4.7	1
2	56	25.2	90	1	1	2	0	0	1	110	78	73	8.5	8.1	8.3	96	15	0.75	254	126	48	180	5.9	0
1	54	25.4	91.5	1	1	2	0	0	1	123	80	69	7.1	6.7	6.9	107	10	0.9	163	111	39	105	6.2	1

1	47	22.2	82.6	2	1	2	0	0	1	118	83	48	7.2	7.2	7.2	92	17	1.04	243	92	47	181	5.4	1
1	52	20.4	76	2	1	2	0	0	0	122	71	63	7.5	7.4	7.45	94	13	0.84	135	53	46	82	5.4	1
1	41	24.1	87.5	2	2	2	0	0	1	124	88	68	6.4	6.2	6.3	93	10	0.87	278	352	51	178	5.4	1
1	49	28.4	98	1	1	2	0	0	0	124	79	59	6.7	6.9	6.8	99	19	1.04	185	100	58	100	5.4	0
1	43	25.3	92	2	2	2	1	0	1	138	105	75	8.3	8.2	8.25	119	13	0.87	179	241	49	102	5.7	0
1	71	19.5	78	1	1	2	0	1	1	128	81	67	9.7	9.3	9.5	120	17	0.88	121	76	62	60	6.1	1
1	58	23.9	85	1	1	1	1	0	1	128	92	60	7.6	7.4	7.5	108	18	1.25	174	173	59	105	5.8	1
1	50	22.5	80	2	1	2	0	0	1	109	78	68	8.2	8.3	8.25	118	8	0.92	238	119	54	164	6	0
1	56	24.6	85	2	2	1	0	0	1	103	70	49	6.7	7	6.85	94	14	0.99	256	99	45	191	5.4	1
1	68	25.0	94.5	2	2	2	1	1	1	169	92	55	8.9	8.9	8.9	124	13	0.79	190	203	58	99	6.6	0
1	49	25.0	89	2	2	2	0	0	0	115	74	71	6.5	6.4	6.45	96	11	0.99	182	56	48	133	5.6	0
1	43	27.2	98.5	1	1	2	1	0	0	139	91	71	7.2	7	7.1	96	14	1.12	172	120	49	111	5.4	0
1	53	18.6	83.5	2	1	2	0	0	1	119	80	61	8	7.8	7.9	93	13	1	253	173	58	177	5.7	0
1	49	25.7	90	2	2	1	1	0	0	155	101	72	8.8	9	8.9	86	8	0.69	234	105	71	142	5.4	1
1	50	23.8	82.5	2	2	2	1	0	0	121	85	64	7.9	8	7.95	107	13	1.01	195	82	70	115	5.3	1
1	69	24.3	87	1	1	1	1	1	1	134	91	57	8.4	8.3	8.35	123	11	0.83	174	137	47	89	6.4	1
1	51	24.6	86.4	1	1	2	0	0	1	120	81	64	6.5	6.6	6.55	88	12	0.82	182	225	49	114	5.3	1
1	58	19.9	78.5	1	1	2	0	0	1	111	73	86	8.1	8	8.05	108	12	0.75	222	79	54	145	5.6	1
1	60	23.5	88	2	1	2	0	0	1	123	95	67	9.1	8.8	8.95	103	12	0.92	206	228	41	134	5.6	0
1	54	25.0	92	2	2	1	1	1	1	132	86	72	7.8	7.7	7.75	153	14	1.01	160	93	67	86	6.2	0
1	51	23.1	83	2	1	2	0	0	0	122	86	66	9	8.8	8.9	88	17	1.05	193	85	63	121	5.4	1
2	64	25.6	85	1	1	1	0	0	1	132	86	86	8	7.8	7.9	112	20	0.74	258	205	51	165	5.8	0
1	53	24.1	90	1	1	2	1	0	1	125	96	62	7.2	7.1	7.15	107	17	0.98	143	81	44	86	5.5	1
2	62	24.5	85	1	1	2	0	0	0	134	84	55	6.9	6.9	6.9	95	17	0.82	202	129	49	124	5.1	0
1	58	26.5	98	2	1	2	0	0	0	130	81	51	8	7.6	7.8	102	16	1.08	209	91	66	137	5.5	0
2	55	25.5	84.8	1	1	2	0	0	1	122	82	52	7	7.1	7.05	93	12	0.68	240	111	50	177	5.7	0
1	54	23.8	86	2	2	2	1	1	1	126	90	68	8.4	8	8.2	132	12	0.7	156	69	54	96	5.9	1
1	57	23.4	81	2	1	2	0	0	0	119	71	44	7	7.4	7.2	104	16	1.07	208	81	53	139	6	1
1	50	27.6	92	1	1	2	0	0	0	129	84	53	7.5	7.8	7.65	115	13	1.03	199	157	47	132	6	1

1	50	23.2	90.5	2	1	2	0	0	1	114	88	50	9.3	9.3	9.3	103	14	0.82	184	223	42	114	5.4	0
1	50	20.0	83	2	1	1	0	0	0	118	81	58	8	7.5	7.75	90	10	0.79	180	163	47	111	5.2	0
1	54	31.4	108	2	2	2	1	0	1	140	93	63	6.4	6.8	6.6	97	11	1	147	213	36	86	5.9	1
1	64	23.1	84	2	1	2	1	0	0	120	84	57	8.5	8.7	8.6	88	15	0.86	205	93	48	148	5.3	1
1	54	23.8	85	1	1	2	0	0	1	136	96	61	6.5	6.7	6.6	95	11	1.15	168	427	37	72	5.3	0
2	56	20.1	75.5	1	1	2	0	0	1	105	66	58	7.3	7.5	7.4	93	12	0.62	194	209	55	110	5.1	1
1	52	23.4	81.5	2	2	2	0	1	1	110	73	61	7.1	7.2	7.15	106	26	0.82	149	110	41	96	6	1
1	35	22.9	82.5	2	2	2	0	0	1	121	72	56	6.8	6.6	6.7	90	10	0.79	241	147	68	156	5.4	0
2	56	19.8	72	1	1	2	1	0	0	133	91	72	7.6	7.5	7.55	92	18	0.9	204	35	88	117	5.9	0
1	72	22.6	87	2	1	2	1	0	1	119	87	62	8.5	8.6	8.55	114	17	0.86	166	95	64	96	5.8	1
2	63	22.1	79	1	2	2	1	0	1	112	81	73	8.8	8.9	8.85	106	17	0.66	199	49	117	76	6.3	0
2	49	25.4	92	1	1	2	1	0	0	146	93	88	7.4	7.3	7.35	94	13	0.69	194	102	72	109	5.3	0
1	51	21.2	79.2	2	1	2	0	1	1	129	86	63	7.5	7.3	7.4	112	11	0.9	123	161	34	71	5.8	0
1	53	25.7	89	2	1	1	0	0	1	126	86	60	6.7	6.5	6.6	99	15	0.87	124	232	54	158	5.3	1
2	49	19.9	75	1	1	2	0	0	1	117	81	84	6.9	6.9	6.9	95	11	0.57	246	142	67	150	5.5	0
1	65	25.5	84	2	2	2	0	0	1	104	63	44	7.9	8.2	8.05	116	12	0.85	196	87	55	138	6.3	1
2	51	31.6	96	1	1	2	0	0	0	128	78	60	5.6	5.8	5.7	101	10	0.63	218	77	55	148	5.9	1
1	60	23.6	92	1	1	1	0	0	1	122	80	74	7.1	7.3	7.2	108	10	0.78	231	110	42	173	5.4	1
1	54	24.4	90	2	1	1	1	0	1	155	100	58	9	8.1	8.55	108	13	0.95	154	178	47	88	5.6	1
1	54	27.6	93.5	2	2	2	1	0	0	154	88	63	7.4	7.2	7.3	104	21	1.11	191	182	59	116	5.5	0
1	54	23.4	85	1	2	2	1	0	1	120	86	68	6	6	6	94	11	0.76	254	120	66	160	5.6	1
2	53	21.6	75	1	1	2	0	1	1	113	78	62	8.7	8.6	8.65	137	15	0.77	217	236	43	138	6.9	1
2	54	22.9	81	1	1	1	0	0	0	131	73	63	7.6	7.8	7.7	88	11	0.57	210	102	59	140	5.6	0
1	50	24.6	86	2	2	2	0	0	1	121	79	56	6.6	6.6	6.6	107	24	0.84	195	300	43	98	5.7	1
1	54	24.5	89.6	2	2	2	1	0	1	140	92	72	7.6	7.5	7.55	93	17	0.93	269	160	66	181	5.5	0
1	45	28.7	93.5	2	2	2	1	0	1	134	96	77	9.9	6.7	8.3	97	9	0.9	202	209	51	129	5.1	0
1	50	21.5	81.3	2	2	1	1	0	1	129	93	84	9	8.2	8.6	115	13	0.73	202	256	59	114	5.4	1
1	55	22.2	89	2	2	2	1	1	0	157	111	78	9.9	8.9	9.4	146	11	0.76	173	139	42	115	6.7	1
1	65	22.5	84	2	1	2	1	1	1	126	77	56	8.1	8.4	8.25	129	20	1.33	156	52	53	100	6.8	0

1	56	22.7	79	2	1	1	0	1	1	122	84	78	8.1	8	8.05	165	10	0.86	192	100	43	99	7.5	0
1	49	29.8	94	2	2	2	0	0	0	127	77	61	6.6	6.5	6.55	106	15	0.76	167	139	50	105	5.7	1
2	53	21.4	78	1	1	2	0	0	0	115	74	56	6.8	7	6.9	84	22	0.75	186	46	75	94	5.4	1
1	52	23.7	81	1	1	1	0	0	0	115	78	56	7.9	8.1	8	85	12	0.94	149	120	48	81	5.5	0
1	59	25.0	85	2	2	2	0	0	0	124	82	61	7.1	7.2	7.15	94	14	0.68	195	90	65	123	5.4	0
1	50	22.3	82	2	2	1	1	0	0	121	89	68	7.6	7.6	7.6	96	14	0.77	210	109	80	111	5.4	0
1	44	24.9	93	2	2	2	0	0	0	121	80	73	7.3	7.2	7.25	110	17	0.83	164	126	54	99	6.1	0
1	57	28.4	94.5	2	2	2	1	0	0	156	100	67	6.2	6.2	6.2	112	9	0.79	170	168	51	95	5.7	1
1	65	32.0	104	2	1	1	0	0	1	132	79	58	6.2	6.2	6.2	115	23	1.18	189	251	30	112	6.3	1
1	62	25.3	91	1	1	2	0	0	1	129	82	61	8	7.9	7.95	85	16	1.11	151	87	48	92	5.6	1
2	61	27.8	87	1	1	1	1	0	1	127	85	62	7.7	8.2	7.95	77	20	0.7	178	75	55	115	5.6	1
1	55	28.4	101	2	1	2	1	0	1	124	84	58	7.8	7.4	7.6	97	21	1.19	217	300	41	129	5.5	1
2	42	19.8	72	1	1	2	1	0	0	137	97	73	7.6	7.3	7.45	97	8	0.79	213	52	58	134	5.5	0
1	52	25.9	87	2	1	2	1	0	1	140	86	79	6.6	6.5	6.55	119	12	1.01	218	237	40	142	5.6	0
1	58	25.9	89	2	1	2	1	1	1	128	91	86	8.2	7.7	7.95	165	11	1.07	150	87	49	92	7.4	0
2	52	20.7	72.2	1	1	2	0	0	0	120	84	61	6.5	6.6	6.55	94	13	0.67	209	132	56	131	5.4	1
1	55	21.4	82	2	1	1	1	0	0	126	90	58	8.9	8.9	8.9	92	14	0.88	216	103	58	145	5.4	1
1	72	26.8	93	1	1	1	1	1	1	159	97	68	9.8	9.2	9.5	265	37	1.58	182	336	30	95	11.8	1
2	58	21.2	75.8	1	1	2	0	0	1	118	69	61	7.7	7.6	7.65	96	18	0.53	204	50	77	104	5.4	1
1	59	28.5	93	1	2	2	1	0	1	174	105	68	10.5	11.9	11.2	93	19	0.83	201	107	50	137	5.7	1
1	60	23.2	84	2	2	2	1	1	1	135	89	68	9.5	9.2	9.35	150	8	0.8	189	56	42	126	8.5	1
1	58	23.7	82	2	1	2	0	0	1	120	81	55	7.8	7.8	7.8	114	16	0.94	239	62	60	162	6.1	1
1	59	25.7	88	2	1	2	0	0	0	122	72	56	7.9	8.5	8.2	95	12	0.93	217	159	40	138	5.8	1
1	52	28.3	96	1	1	1	0	0	1	118	76	55	8	7.8	7.9	108	11	0.86	147	111	52	84	5.8	0
1	57	27.1	94	1	1	2	1	0	0	144	103	54	7.8	7.6	7.7	94	14	0.99	151	121	40	70	5.4	0
1	59	28.3	93	2	2	2	1	0	0	140	93	65	7.6	8.1	7.85	108	13	0.88	177	109	51	119	5.6	1
1	60	21.4	75	1	1	1	0	0	0	106	69	51	7.9	8.3	8.1	80	19	1.02	180	64	48	113	5.9	1
2	53	21.7	80	1	2	2	0	0	1	98	62	50	7.8	8	7.9	93	21	0.77	229	70	71	135	5.7	1
2	55	19.9	76.8	1	1	2	0	0	1	131	77	60	9.4	8.5	8.95	94	10	0.7	244	153	55	165	5.4	0

2	51	21.6	81	1	1	1	0	0	1	123	71	58	6.9	7.1	7	86	14	0.74	219	139	43	147	5.8	0
1	44	23.0	80	1	2	1	0	0	1	130	81	58	8.2	7.9	8.05	106	11	0.83	127	38	39	79	5.7	0
1	53	23.4	85.5	2	2	2	1	0	1	127	87	63	8.2	8.5	8.35	119	17	0.97	190	172	59	110	6.2	0
1	51	24.3	90	2	2	1	0	0	0	115	82	77	7.9	7	7.45	95	8	0.89	235	165	55	157	5.4	1
1	51	26.1	98	2	2	2	0	0	0	121	76	72	6.9	6.8	6.85	113	13	0.75	193	130	57	116	5.5	0
1	53	24.0	83	2	1	1	1	0	1	126	89	59	7.4	7.3	7.35	112	16	0.91	149	207	43	83	5.7	1
2	61	25.4	90	1	1	1	0	0	0	126	75	71	7.9	7.8	7.85	105	18	0.71	205	66	85	120	5.4	0
1	51	22.3	85	2	2	2	1	0	1	136	91	62	7.9	7.7	7.8	94	11	0.88	209	212	46	130	5.5	1
2	59	24.5	85.5	1	1	1	0	0	0	117	75	59	7.4	7.5	7.45	97	11	0.67	199	80	70	121	5.1	1
1	51	26.4	92	2	1	2	0	0	0	119	76	62	7.8	7.7	7.75	104	17	1.06	227	100	42	157	5.8	1
1	57	24.9	95.6	1	1	2	1	0	1	135	96	84	8.2	7.9	8.05	116	10	0.66	239	284	51	155	5.6	1
1	58	26.3	91.5	2	1	2	1	0	0	133	85	55	8	7.4	7.7	110	16	0.91	202	115	60	122	5.6	0
1	47	25.5	94	1	1	1	1	1	1	140	101	67	7.9	7.3	7.6	112	23	1.11	197	205	50	108	6.5	0
1	55	21.5	80	2	2	2	0	0	0	115	70	54	7.3	7.2	7.25	94	18	0.92	214	161	51	131	5.4	1
1	53	23.3	83	2	1	2	1	0	1	129	82	46	7.3	7.2	7.25	92	12	0.93	181	63	76	95	5.9	0
1	53	22.0	80	1	1	1	1	1	1	140	94	65	7.5	7.8	7.65	135	17	1.18	256	157	60	159	6.9	0
1	51	23.2	85	2	2	2	0	1	1	113	80	66	8.3	8.2	8.25	148	16	0.76	105	65	44	64	7.4	0
2	60	23.8	84.8	1	1	1	0	0	1	125	75	54	7.3	7.7	7.5	101	18	0.73	214	90	55	150	5.6	0
1	52	25.2	90	1	1	2	0	0	0	120	80	55	8.2	8.3	8.25	95	10	1	161	55	66	93	5.8	1
1	44	23.0	87.6	2	1	2	0	0	1	129	76	65	7.9	8.2	8.05	113	14	0.95	146	228	45	83	5.4	0
1	52	23.1	92	2	2	1	1	0	0	133	94	65	8	8	8	104	15	0.84	168	149	52	103	4.8	0
2	58	22.2	75	1	1	2	0	0	0	116	64	46	7.9	7.8	7.85	97	13	0.71	215	98	77	126	5.5	0
1	67	24.4	88	2	1	1	0	0	0	119	77	59	14.8	11.2	13	102	16	1.03	196	159	47	125	5.9	0
1	70	26.2	92	2	2	2	1	0	0	141	91	47	6.6	6.8	6.7	103	15	1.14	197	79	68	124	6	0
1	48	25.3	90.5	2	2	1	1	0	0	145	99	72	7.5	7.6	7.55	98	14	0.82	222	134	48	155	5.4	1
1	65	23.3	90	1	1	2	1	1	1	137	84	65	9.4	9.3	9.35	136	16	0.79	179	165	45	104	6.1	0
1	52	23.1	83	2	2	1	0	0	0	130	86	70	7.2	7	7.1	97	15	1.09	182	79	49	122	5	1
1	72	21.1	78	2	2	2	0	1	0	120	75	54	9.8	10.2	10	164	22	0.69	159	68	67	84	6.5	0
1	70	23.1	87.2	2	2	2	1	0	1	149	95	69	7.9	7.8	7.85	107	13	0.74	166	130	48	107	5.9	1

1	46	24.6	85	1	1	2	0	0	1	114	75	62	7.7	7.7	7.7	98	13	0.83	153	348	35	91	5.2	0
1	49	29.4	95	2	2	2	1	0	1	169	115	66	6.8	6.9	6.85	102	12	0.77	271	167	52	187	5.3	0
2	54	24.6	86	1	1	1	1	0	0	136	91	59	6.8	6.4	6.6	86	17	0.62	190	61	58	114	5.6	1
1	49	29.9	103.3	2	2	2	1	0	1	134	93	72	7.5	7.2	7.35	95	16	0.96	216	362	44	108	5.3	1
1	54	21.9	78.4	2	1	1	0	0	0	110	75	67	8.8	9	8.9	84	13	0.96	207	41	56	153	5.1	1
1	50	23.5	83	2	1	1	1	0	0	117	84	52	7.2	7.2	7.2	89	23	0.99	172	74	41	122	5.3	0
2	53	28.3	93	1	1	1	1	0	0	133	90	58	7.6	7.5	7.55	116	18	0.6	207	161	48	143	6.2	1
1	63	21.6	80	2	2	1	1	0	0	135	104	88	9.6	9.1	9.35	88	11	0.72	168	58	82	74	5.3	1
1	60	24.0	85	2	1	2	1	1	1	148	98	64	11	10	10.5	115	17	0.9	176	181	38	110	6.1	1
1	49	23.4	85.5	2	1	2	1	0	1	142	96	60	7.2	7.2	7.2	104	11	1.05	238	98	43	169	5.6	1
1	49	30.4	104	2	1	2	1	0	0	147	111	65	7.3	6.8	7.05	108	17	1.11	180	156	48	121	5.4	1
1	58	22.5	85	2	1	2	0	1	0	120	85	60	8.1	8.7	8.4	128	16	0.97	169	95	66	96	6.5	1
1	56	28.7	93	1	1	2	0	0	0	130	85	74	7.9	7.9	7.9	117	14	0.71	201	164	46	127	5.6	1
1	63	24.6	87	2	1	2	0	0	1	122	79	55	8.6	8.7	8.65	91	23	0.89	224	135	40	160	5.5	1
1	46	24.2	85	2	2	1	0	0	0	130	85	70	6.8	7	6.9	96	15	0.72	191	52	67	100	5.4	0
1	54	25.0	90.5	1	1	1	1	0	0	132	97	61	8.7	8.6	8.65	99	13	0.82	235	91	78	153	5	0
1	74	21.5	83	1	1	2	1	1	1	145	90	56	9.5	9.6	9.55	121	16	0.7	192	108	54	118	7.2	1
1	63	25.4	89.5	2	2	1	1	0	1	143	101	70	9.5	8	8.75	94	21	0.93	186	219	46	113	5.3	1
1	54	23.5	86	2	1	1	0	1	1	122	83	64	7.3	7.3	7.3	137	13	0.77	203	102	63	128	6.5	0
1	54	20.6	76	2	1	2	0	0	0	123	83	53	6.4	6.4	6.4	99	21	0.96	200	71	84	94	5.5	0
1	55	26.7	89	2	1	2	1	1	0	133	93	71	8.4	8.4	8.4	147	17	0.95	142	94	53	78	7.7	0
1	45	24.2	87.5	1	1	1	0	0	0	107	76	73	8.9	8.4	8.65	93	12	0.91	228	105	43	157	5.6	1
1	47	24.0	86.7	2	1	1	0	0	1	126	82	74	6.4	6.6	6.5	94	14	0.93	241	71	56	174	5.7	1
1	49	21.4	81	1	1	2	0	0	0	106	72	56	6.8	6.8	6.8	87	19	1.12	166	72	52	104	5.4	1
1	45	25.5	89	2	2	2	0	0	1	131	89	69	7.6	7.4	7.5	99	14	1.28	287	212	59	181	5.6	1
1	58	22.2	80	2	1	1	1	0	1	149	108	59	8.2	8	8.1	97	18	1.16	187	138	56	105	5.9	0
1	47	21.7	79.6	2	1	1	0	0	0	101	67	56	7.4	7.7	7.55	93	12	0.9	193	88	48	131	5.7	0
1	55	28.4	101	2	2	1	1	0	1	137	84	56	6.6	6.8	6.7	122	18	0.99	183	167	53	109	6.1	1
1	48	25.5	93	2	2	1	0	0	1	129	81	64	6.7	6.7	6.7	107	11	0.78	176	116	45	121	6	1

1	57	22.1	83	2	1	2	0	0	1	108	76	57	7.9	7.7	7.8	90	11	0.92	232	116	41	178	5.9	1
1	56	26.9	92	2	2	2	0	0	0	112	65	50	6.9	6.7	6.8	88	16	0.98	212	70	67	132	5.2	1
1	50	23.7	87.5	2	1	2	0	0	1	106	71	62	7.9	7.9	7.9	94	15	0.97	129	60	37	86	5.5	1
2	52	27.4	86	1	1	2	1	1	1	148	89	61	9.7	8.4	9.05	127	20	0.62	211	258	49	131	7.8	0
1	54	32.4	109	2	2	2	1	1	1	135	88	76	6.4	6.9	6.65	157	14	0.89	176	162	54	98	6.3	0
1	56	27.1	99	2	1	2	1	1	0	105	71	48	7.2	7.2	7.2	129	16	0.81	164	86	49	102	6.6	1
1	56	25.9	83.8	2	2	2	0	0	1	113	75	62	6.9	7	6.95	105	15	1.03	248	79	80	156	5.1	1
1	54	26.4	94.5	2	1	2	0	1	1	129	85	48	7.3	7.4	7.35	134	12	0.81	155	147	39	93	6.6	1
2	53	28.8	95	1	1	2	0	0	1	118	82	74	5.7	5.9	5.8	89	10	0.69	165	80	50	99	5.7	0
2	58	22.1	75	1	1	2	0	0	0	111	76	56	7.7	7.6	7.65	87	14	0.68	199	107	68	108	5.6	1
1	50	22.8	83	2	1	2	0	0	0	133	86	59	7.5	7.6	7.55	114	22	0.77	184	80	49	121	6.3	0
1	52	26.4	93.5	2	2	2	1	1	1	135	92	62	7.2	7.1	7.15	140	13	0.99	249	432	35	146	6.5	1
1	49	26.9	94	2	1	2	0	0	0	128	86	66	7.4	7.3	7.35	106	11	0.96	228	110	49	153	5.5	1
1	51	22.0	82	2	2	1	0	0	0	122	87	60	8.8	8.3	8.55	107	15	0.94	206	62	55	137	5.4	1
1	49	25.5	83	2	1	1	1	0	1	144	107	67	7.1	7.1	7.1	94	15	0.83	229	152	53	162	5.9	1
1	71	26.8	89	2	2	2	0	0	1	132	90	63	7.9	8.4	8.15	106	16	1	204	92	57	128	6.1	1
1	45	23.2	86	2	1	2	0	0	0	112	68	60	8	7.9	7.95	101	13	0.82	183	141	49	116	5.9	0
1	55	23.7	87	1	1	2	0	0	1	126	86	58	6.9	7	6.95	98	15	0.8	211	215	35	144	5.4	1
1	60	24.9	93.5	2	1	2	1	1	1	155	95	68	8.4	8.4	8.4	141	17	0.89	126	45	58	61	6.6	1
1	58	25.7	89	2	2	2	1	0	1	137	88	48	7.9	7.9	7.9	104	17	0.91	241	180	76	145	5.7	1
1	55	21.6	83	2	1	2	0	0	0	115	69	58	7.2	7.1	7.15	91	14	0.87	236	178	58	153	5.2	1
1	56	27.0	90	1	2	2	1	0	0	169	96	57	7.8	7.8	7.8	114	15	0.8	155	60	44	110	5.7	1
2	54	21.6	80	1	1	2	1	0	0	140	86	64	7.2	7.2	7.2	91	14	0.68	183	69	69	114	5.4	0
1	53	22.7	82.8	2	2	2	1	0	0	137	90	99	9.8	8.6	9.2	87	13	1.17	170	60	48	111	5.4	1
1	44	24.1	86.1	1	1	1	0	0	0	113	76	67	6.5	6.5	6.5	77	15	0.99	215	119	47	156	5.2	0
1	53	28.4	108	2	1	2	0	0	0	120	89	65	8	7.7	7.85	95	25	1.16	208	158	41	142	5.8	1
1	55	22.5	83.5	2	1	2	1	0	0	137	88	68	8.6	7.8	8.2	111	14	0.96	202	109	54	132	5.5	1
1	48	26.2	91.5	2	1	1	1	0	1	119	82	58	7.3	7.2	7.25	98	15	0.79	123	103	50	57	5.5	0
1	64	20.9	76	2	1	2	0	0	0	136	93	75	7.9	7.8	7.85	107	13	0.77	180	95	44	134	5.4	0

1	61	27.0	93	1	1	1	1	1	1	133	90	70	10.3	9.4	9.85	111	13	0.89	127	133	42	86	8.5	1
1	61	26.5	96	2	1	2	0	0	1	122	82	60	7.8	8.1	7.95	97	16	0.98	193	124	36	139	6.3	1
1	58	27.3	92.5	2	1	2	1	1	0	143	89	72	7	6.7	6.85	197	14	0.65	185	93	61	111	8	1
1	52	24.1	94	2	2	2	1	0	0	125	93	66	7.2	7.1	7.15	114	15	1.04	214	140	51	141	5.9	1
1	58	20.2	80	1	1	2	0	0	1	114	81	58	8.2	7.7	7.95	83	18	0.93	179	291	41	74	5.4	0
1	50	25.9	90	2	2	2	1	0	1	161	105	66	7.4	7.4	7.4	90	14	0.96	253	201	63	151	5.3	1
1	52	26.0	93.5	2	2	1	1	1	0	145	97	52	8	8.2	8.1	151	20	0.89	207	182	54	127	6.9	1
1	60	30.5	103	2	1	2	1	0	1	140	88	61	9.2	8.9	9.05	87	17	0.78	159	78	38	112	6.4	1
1	49	26.4	94	2	2	1	1	0	1	121	89	68	7.2	6.9	7.05	102	14	1.06	202	276	37	136	5.6	1
1	48	25.8	90	1	2	2	1	0	0	119	85	63	7	7	7	99	14	0.98	164	121	46	101	5.4	0
2	54	21.6	71	1	1	2	0	0	0	115	75	70	7.4	7.7	7.55	94	22	0.8	195	67	51	141	5.6	1
1	58	24.5	87	2	1	2	0	0	1	121	79	54	7.5	7.3	7.4	75	13	0.99	212	57	57	139	5.6	1
1	50	24.4	89.5	2	2	2	1	0	0	124	83	52	7.7	8.2	7.95	101	10	1.11	202	89	72	111	5.4	0
1	57	26.2	91	2	2	2	0	0	1	119	89	59	8	7.8	7.9	97	12	0.77	281	115	73	194	5.6	1
1	57	22.0	82.5	1	1	1	1	0	1	131	90	67	8.9	8.5	8.7	97	18	1.16	185	102	58	112	5.5	1
1	49	26.7	91.5	2	2	1	1	0	0	132	90	64	6.6	6.5	6.55	102	14	0.89	202	85	54	125	5.7	0
1	54	23.3	85	1	1	2	1	0	1	140	92	86	7.1	6.8	6.95	102	12	1.17	172	109	38	118	5.7	1
1	55	24.3	85	2	2	2	1	0	1	131	90	53	9.2	9.1	9.15	86	14	1.26	213	184	38	138	5.6	1
2	54	24.4	81.5	1	1	2	1	0	1	142	88	68	7.3	7.3	7.3	98	14	0.69	198	190	45	115	5.9	1
2	56	17.5	63	1	1	2	1	0	0	131	90	62	7.8	8.2	8	94	7	0.57	174	57	63	94	5.4	1
1	44	26.4	94	1	1	2	1	0	1	137	93	64	7.3	7.6	7.45	108	12	1.08	207	130	36	153	5.9	0
1	48	29.7	99	2	1	1	1	1	1	143	87	70	7.3	7.5	7.4	148	10	1.03	150	135	44	99	7.5	1
1	48	25.4	89	2	1	2	0	0	0	107	75	56	7.2	7	7.1	79	11	0.91	142	74	46	79	5.6	0
1	43	26.5	93	1	1	2	0	0	1	136	94	73	6.3	5.9	6.1	110	15	0.89	176	147	44	119	6	0
1	57	26.7	86	2	2	2	1	1	1	141	95	58	6.7	7.1	6.9	141	17	0.98	182	147	50	124	6.5	1
1	52	30.8	103	2	2	2	1	0	1	119	85	53	6.9	6.7	6.8	85	12	0.91	185	132	35	127	5.5	0
1	53	24.1	88	2	2	1	0	0	1	126	87	63	8	8.4	8.2	107	14	1.14	202	407	38	94	6.1	1
1	55	24.3	88	2	1	1	1	1	0	136	95	69	7.9	7.6	7.75	134	13	0.93	229	194	46	155	7.8	0
2	57	18.8	69	1	1	1	0	0	0	107	67	70	7.9	8	7.95	83	16	0.71	176	45	69	103	5.7	1

1	55	24.7	88	2	1	2	0	0	1	107	74	60	8.2	8.5	8.35	101	15	1.05	186	142	38	125	5.5	0
1	55	27.4	92	2	1	2	0	1	1	126	82	61	7.6	7.5	7.55	114	17	0.85	184	80	59	109	6.7	1
2	47	30.4	113	1	1	1	0	0	1	134	84	65	7.2	7.4	7.3	112	10	0.51	179	79	54	114	5.4	0
1	58	26.2	90	2	1	2	1	0	1	143	90	66	7	7.2	7.1	123	14	1.15	242	151	53	167	5.9	1
1	59	24.2	89	1	1	2	0	0	0	109	67	57	7.4	7.4	7.4	89	16	0.91	169	126	41	109	5.3	1
2	58	22.6	87.5	1	1	1	0	0	0	111	71	49	7.4	7.4	7.4	88	13	0.58	202	118	49	126	5.9	0
1	60	23.5	82	1	1	2	0	0	1	132	89	70	7.2	7.3	7.25	106	13	0.87	146	53	69	86	5.6	1
1	62	24.7	89	2	1	2	1	0	0	145	95	78	8.4	8.2	8.3	112	24	1.12	210	188	48	143	6	0
1	55	26.3	93	2	1	1	0	0	1	112	81	65	7.5	7.4	7.45	90	13	0.91	244	144	47	157	5.8	0
1	48	26.4	94	2	2	1	0	0	0	132	87	58	7.4	7.5	7.45	105	13	0.98	169	134	54	96	5.4	1
1	49	27.4	91	2	1	1	1	0	0	126	87	68	5.8	5.8	5.8	93	11	1.02	187	155	47	120	4.9	1
1	59	23.3	80	2	1	1	0	0	0	114	76	55	7.4	7.6	7.5	99	20	1.01	135	101	43	77	5.4	1
1	54	24.3	81	2	1	2	1	1	1	125	89	57	6.8	7.1	6.95	111	9	0.85	135	95	52	75	6.3	0
1	60	23.0	83.6	2	2	1	1	0	0	132	95	50	7.9	7.8	7.85	94	15	0.74	165	48	69	102	5.6	1
1	55	21.2	83	2	1	2	0	1	0	117	79	57	8.2	8.3	8.25	127	18	0.85	184	162	48	117	6	0
1	58	22.3	80	2	1	2	0	1	0	137	89	55	8.4	8.2	8.3	130	16	0.95	209	51	99	109	6.1	0
2	57	22.7	79	1	1	2	1	0	0	138	109	84	8.9	8.6	8.75	103	15	0.59	210	60	65	132	5.6	1
1	47	20.4	70	1	1	2	0	0	1	109	75	51	7.8	8	7.9	84	14	1.08	241	60	57	156	5.5	0
1	53	22.1	84.5	2	1	2	0	0	1	121	88	64	7.7	7.5	7.6	97	11	0.93	175	95	41	122	5.9	1
2	55	23.1	86	1	1	1	0	0	0	136	84	59	6.8	6.6	6.7	92	11	0.57	216	162	56	138	5.8	1
1	59	21.8	78	2	2	1	0	0	0	120	82	55	7.2	7.3	7.25	98	16	1.15	232	163	43	158	5.5	1
2	54	26.1	85	1	1	2	1	1	1	139	91	69	6.6	6.4	6.5	110	14	0.7	194	80	36	153	6.2	1
2	52	18.7	66	1	1	2	0	0	1	105	69	61	7.9	7.9	7.9	78	19	0.62	256	116	62	150	5.5	1
1	56	23.7	88.2	2	1	2	1	0	0	110	79	63	7.2	7.2	7.2	86	21	0.97	185	68	66	112	5.1	1
1	66	22.7	84	2	1	2	1	0	1	137	90	77	7.9	7.9	7.9	108	14	0.88	205	166	37	150	5.3	0
1	51	21.6	82.5	2	2	2	1	1	0	128	83	69	8.6	8.8	8.7	127	11	0.89	179	131	91	91	6.3	1
1	52	18.5	70.5	2	2	1	0	1	0	124	84	76	8	7.8	7.9	138	11	0.91	186	120	81	98	5.7	1
2	74	23.5	83	1	1	2	1	0	1	126	72	63	10.2	10	10.1	91	24	1.06	146	97	45	89	5.6	0
1	49	19.2	74.5	2	2	2	0	0	1	111	84	59	8.6	8.7	8.65	107	17	0.98	146	266	48	81	5.2	0

1	49	28.1	96	2	2	1	1	0	1	128	87	65	6.5	6.3	6.4	97	12	0.92	237	156	47	160	5.4	1
1	51	26.3	89	1	2	2	0	0	0	129	88	50	5.9	5.9	5.9	108	20	1.01	177	58	50	119	5.4	1
1	56	22.6	84	2	2	2	0	0	0	119	70	57	7.9	7.8	7.85	107	13	0.81	216	177	45	129	5.5	1
1	66	26.0	98	2	1	1	1	0	0	135	94	65	7	7	7	95	15	0.94	163	68	60	103	5.4	1
1	59	23.9	91	2	2	2	1	0	1	144	102	55	9.6	8.7	9.15	92	14	1.2	245	113	45	161	6.1	1
1	48	26.7	89	1	2	2	0	0	0	137	86	71	7.2	6.8	7	92	13	1	170	70	42	119	5.8	1
2	71	21.9	84	1	1	1	1	0	1	119	74	61	9.2	9.1	9.15	82	12	0.57	146	136	45	77	5.5	1
1	60	20.8	82	2	2	2	0	1	0	127	85	67	8.8	9.2	9	156	16	0.83	135	193	41	67	7.5	1
2	52	17.7	67	1	1	2	0	0	0	127	79	86	6.9	6.8	6.85	97	13	0.51	207	31	117	95	5.4	1
1	53	24.7	88	2	1	2	1	1	1	116	79	73	7.3	7	7.15	128	14	0.91	216	212	48	138	6.4	0
1	54	21.4	77	2	1	2	1	0	0	105	73	55	7.6	7.4	7.5	123	16	1.1	108	89	40	60	5.8	1
1	53	24.8	87.5	1	1	2	0	0	1	123	85	56	7.7	7.8	7.75	92	12	1.08	198	203	46	112	5.9	0
1	61	27.8	104	1	1	2	0	0	0	117	83	48	8.9	9.2	9.05	118	15	1.05	151	83	47	86	6.1	1
1	56	23.5	83.5	2	1	1	1	0	0	106	75	56	6.6	6.5	6.55	87	17	1.08	135	49	48	86	5.3	1
2	56	24.1	86.5	1	1	2	0	0	0	122	89	81	9.4	8.5	8.95	96	15	0.89	228	74	63	152	5.8	0
1	56	24.8	93.5	2	1	2	1	0	1	134	95	68	8	7.7	7.85	90	17	0.79	163	137	38	105	5	1
2	58	22.5	79	1	1	1	0	0	0	106	76	60	7.6	7.7	7.65	93	19	0.74	231	81	70	148	5.5	1
1	53	26.7	98.5	2	1	2	0	0	1	119	82	65	7.1	7.1	7.1	98	10	0.9	199	153	34	135	5.5	1
2	51	19.2	74	1	1	2	0	0	0	123	77	59	7.2	7.2	7.2	88	12	0.63	201	80	63	119	5.4	0
1	50	25.8	91	2	1	2	1	1	1	103	71	62	7.3	7.4	7.35	261	14	0.77	196	137	34	140	12.6	0
1	54	25.5	80	2	2	2	1	0	0	133	90	63	7.3	7.1	7.2	104	14	0.76	211	89	74	121	5.6	0
1	49	25.4	92	2	2	1	0	0	0	119	72	69	7.5	7.5	7.5	107	16	0.9	193	112	43	125	5.8	1
1	42	25.9	89.3	2	2	1	0	0	1	128	87	77	7.1	6.9	7	101	15	1.06	234	82	49	177	5.7	0
2	51	21.2	77	1	1	2	0	0	1	102	65	55	6.6	6.6	6.6	86	15	0.64	236	98	55	163	5.2	0
1	59	26.2	88.5	2	1	1	1	0	0	134	96	72	8.8	8.4	8.6	105	19	0.85	196	137	58	112	5.1	1
1	48	23.5	84	2	2	2	0	0	0	123	85	82	7.1	7.2	7.15	93	13	0.85	209	137	68	126	5.7	0
1	55	25.8	88	2	1	1	1	0	1	112	75	56	8.9	8.7	8.8	100	18	0.96	149	80	55	89	5.8	1
1	49	24.2	92	2	2	2	1	1	1	128	91	67	7.6	7.5	7.55	114	16	0.62	161	66	39	118	6.6	0
1	53	26.1	90	1	2	2	0	0	0	112	77	46	7.5	7.6	7.55	102	12	1	197	121	67	124	5.8	0

1	69	22.2	83	2	1	2	0	0	0	135	82	63	9.3	9.1	9.2	101	11	0.83	187	73	66	116	5.5	1
1	52	21.1	81	2	2	2	0	0	0	115	69	58	8	7.7	7.85	92	17	0.91	178	113	50	104	4.9	1
1	47	25.1	91	2	2	2	1	0	1	147	91	64	8.5	8	8.25	102	17	0.67	245	158	67	164	5.3	0
1	54	26.0	96.5	2	2	2	0	0	0	116	81	50	7.9	8.3	8.1	107	19	1.07	224	193	46	156	5.6	1
1	53	27.4	91.5	1	2	1	0	0	0	125	83	57	6.2	6.3	6.25	98	14	0.94	213	90	46	149	5.6	1
1	53	28.7	103	1	1	2	0	0	1	123	85	72	7.8	7.2	7.5	108	19	0.97	261	211	51	180	5.6	1
1	53	20.4	79	2	1	2	0	0	1	120	79	68	7.9	8.1	8	97	13	0.91	187	170	39	124	5.4	1
1	57	24.3	82.5	2	1	2	1	0	0	133	96	69	7.3	7.6	7.45	91	11	0.97	183	87	50	126	5	0
1	73	25.0	95	2	1	2	0	0	0	116	85	67	13.4	10.8	12.1	90	19	1.27	179	98	45	119	6.1	1
1	50	30.3	104	2	2	2	1	0	1	105	75	79	8.4	8.4	8.4	109	12	1.15	215	540	35	96	5.6	1
1	55	23.9	84.5	2	2	2	1	0	0	109	72	60	6.6	6.6	6.6	97	15	1.28	197	157	43	118	5.4	1
1	48	24.4	91	2	2	2	0	0	0	130	89	61	7.3	7.1	7.2	107	19	0.98	141	82	47	78	6.3	1
1	43	24.9	89	1	1	1	1	0	1	132	87	68	6	6.2	6.1	93	8	0.86	122	153	39	69	5.5	0
2	44	20.9	78	1	1	1	0	0	0	111	75	57	5.6	5.8	5.7	95	11	0.68	170	98	54	93	5.8	0
1	54	22.9	87.5	1	1	2	0	0	1	122	81	61	8.5	8.2	8.35	91	19	0.95	250	99	45	190	5.4	0
1	48	27.1	96	2	2	2	1	0	0	148	102	72	7.4	7.2	7.3	112	15	0.79	223	43	65	155	5.8	0
2	47	21.5	71	1	1	2	0	0	0	98	66	68	7.1	7.1	7.1	93	10	0.57	198	47	57	125	5.7	0
1	50	26.1	88.2	1	1	2	0	0	0	112	76	71	7.5	7.6	7.55	97	11	0.88	209	104	56	141	5.3	1
1	56	26.4	91	1	1	1	1	0	0	132	92	54	7.7	7.6	7.65	106	17	0.82	176	83	58	107	5	1
2	49	21.5	81	1	1	2	0	0	0	116	66	54	5.8	6	5.9	94	15	0.59	176	32	66	86	5.4	1
1	56	22.6	80.4	2	1	2	0	0	0	127	81	75	7.8	7.8	7.8	98	13	0.94	231	56	61	156	4.9	1
2	64	28.5	97.5	1	1	1	1	1	1	141	90	61	7.4	7.2	7.3	155	14	0.68	130	117	45	72	7.9	0
1	48	24.7	83.5	2	1	1	0	0	1	122	77	65	6.8	6.7	6.75	101	19	1.03	243	202	47	173	5.5	0
1	48	27.4	104	2	2	2	0	0	1	124	82	93	7.1	7.1	7.1	116	18	0.78	285	295	90	169	6	1
1	50	28.9	103	2	1	2	1	1	0	121	79	54	8	8	8	131	16	0.92	191	173	46	123	6.7	1
1	48	26.8	95	1	1	2	1	0	1	131	90	79	6.8	7	6.9	102	12	1.17	160	125	47	100	5.8	0
2	66	34.3	107	1	1	1	1	0	1	154	88	60	7.1	7.5	7.3	95	12	0.58	198	133	81	87	6	0
1	49	24.7	85	2	1	2	0	0	1	118	81	58	8.4	8.3	8.35	92	12	0.84	124	84	39	75	5.3	1
1	46	29.0	91	2	1	1	1	0	0	133	96	77	7.1	7.1	7.1	109	10	0.73	173	198	48	111	5.3	1

1	47	29.3	102	2	2	2	0	0	0	133	87	64	7.4	7.6	7.5	103	13	0.87	153	92	49	95	5.2	0
1	66	23.2	89	2	1	2	0	0	1	123	79	78	7.9	8	7.95	90	14	0.89	189	128	37	126	5.3	0
1	52	22.5	89	2	1	1	0	0	1	129	80	64	7.7	7.4	7.55	92	13	0.98	270	449	35	136	6	0
1	50	24.3	91	2	2	2	1	0	1	119	81	73	7.9	8	7.95	95	14	0.95	146	94	71	75	5.6	0
1	54	29.4	101	2	2	1	1	0	0	140	89	60	9.8	7.3	8.55	110	12	0.91	152	147	44	88	5.9	1
1	39	24.7	82	2	1	2	0	0	0	121	71	51	6.1	6.3	6.2	88	18	0.94	182	79	48	122	5.4	1
1	48	23.8	90.4	2	2	2	1	0	0	134	94	71	6.8	6.5	6.65	119	10	0.79	154	195	45	80	5.5	1
2	48	21.8	74	1	1	1	0	0	1	111	75	51	7.4	7.3	7.35	100	10	0.67	250	64	62	180	5.1	1
1	58	25.3	92.4	2	1	2	0	0	1	101	75	52	7.8	7.9	7.85	110	14	0.88	168	114	54	84	5.9	1
1	52	23.5	90	2	1	1	0	0	1	112	74	56	7.8	7.7	7.75	90	15	0.78	128	187	27	70	5.6	0
1	49	25.4	89	1	2	2	1	0	0	135	94	67	8.2	7.7	7.95	100	13	1	196	160	47	125	5.6	0
1	59	23.7	85	2	1	1	0	0	0	109	74	63	7.4	7.1	7.25	85	14	1.01	186	81	42	129	5.7	1
1	55	25.4	90	2	1	1	0	0	0	121	85	72	7.7	7.2	7.45	87	13	0.99	205	161	46	134	5.4	1
2	64	20.4	80.5	1	1	1	1	0	1	142	90	93	9	8	8.5	88	12	0.6	163	76	57	98	5.6	0
1	49	27.0	93.5	2	2	2	1	0	0	128	93	63	6.3	6.4	6.35	104	14	0.88	217	127	48	148	5.4	1
1	54	27.5	96.5	2	2	2	1	0	1	133	85	58	8.1	7.6	7.85	112	19	0.85	149	226	43	75	5.5	1
1	51	23.3	86	2	1	2	1	0	1	135	93	73	7.7	7.7	7.7	119	12	1.04	210	305	43	109	5.7	1
1	50	20.8	75.5	1	1	1	0	0	1	119	84	70	7.6	7.6	7.6	92	17	0.81	160	386	31	59	5.6	0
1	57	22.6	86	2	1	2	1	1	1	110	76	85	7.5	7.6	7.55	121	8	1.01	128	208	41	64	7.2	0
1	53	25.4	88	2	1	1	0	0	0	110	75	67	6.9	7	6.95	92	14	0.99	173	74	52	111	6	0
1	51	27.4	88.5	2	1	1	1	0	1	135	86	66	7.2	7.3	7.25	87	18	1	187	218	37	127	5.4	1
1	50	26.5	89	2	1	2	0	0	1	134	89	66	7.8	8	7.9	107	18	0.88	278	185	41	213	5.8	1
1	71	25.5	93.5	2	1	1	0	0	1	127	82	64	8.3	8.4	8.35	112	23	0.91	169	100	39	119	5.3	1
2	51	21.2	79	1	1	1	0	0	1	110	76	75	6.7	6.6	6.65	87	8	0.72	254	132	81	140	5.7	1
1	53	22.7	81.5	1	1	1	0	0	0	122	80	60	7	6.8	6.9	97	11	0.85	216	109	47	152	5.3	1
1	44	28.4	95.5	2	2	2	1	0	0	137	90	62	6.7	6.3	6.5	105	11	0.91	214	148	50	151	5.7	0
1	69	24.9	91.5	2	1	2	0	0	1	123	83	63	8.1	8.5	8.3	97	15	1.03	173	88	68	96	5.2	1
1	51	28.4	101	2	1	1	0	0	1	138	92	71	6.8	6.6	6.7	99	14	0.96	194	214	49	118	5.6	0
1	46	25.3	86.5	2	1	2	0	0	0	127	84	67	6	6	6	96	12	1.01	186	162	52	110	5.4	1

1	38	24.0	94.5	2	2	2	0	0	0	134	78	60	7	6.8	6.9	91	12	1.09	203	149	53	127	5.2	0
1	41	28.1	98.7	2	1	2	1	0	0	141	85	62	5.9	5.8	5.85	107	17	0.72	185	126	46	128	5.4	1
1	56	25.4	86.4	2	1	2	1	0	0	130	95	73	7.6	7.6	7.6	104	13	0.89	181	128	53	116	5.1	1
1	42	26.4	96	2	1	2	1	0	1	124	85	75	7.2	7.3	7.25	97	14	0.89	142	148	38	94	5.5	1
1	40	23.5	84.5	2	1	1	0	0	0	123	81	52	6.8	6.5	6.65	95	15	0.88	177	95	49	113	5.3	1
1	35	22.9	85.5	2	1	2	0	0	0	119	72	65	5.9	6.1	6	84	12	1.05	177	83	66	100	5.1	1
1	49	24.5	84	2	1	2	1	0	0	142	94	63	6.4	6.4	6.4	99	20	0.98	194	88	61	115	5.3	0
2	66	19.9	73	1	1	2	1	0	1	140	84	73	8.4	8.7	8.55	103	11	0.61	257	87	76	163	5.8	1
1	49	22.7	85	2	1	2	0	0	0	114	75	60	6.7	6.7	6.7	89	13	0.83	220	138	47	155	5.4	0
1	54	24.0	92	1	1	2	1	0	1	120	85	62	7.3	7.3	7.3	94	15	0.99	179	126	38	127	5.3	1
1	52	28.6	96	2	1	2	1	0	1	145	97	63	8	8.3	8.15	101	19	1.23	261	392	41	148	5.5	1
1	54	25.6	90	1	1	2	1	0	1	131	94	57	6.5	6.3	6.4	102	19	0.99	188	143	56	112	5.4	0
1	53	22.8	86.9	2	1	2	0	1	0	131	88	58	7.8	7.9	7.85	142	13	0.75	213	166	41	149	6.4	1
1	46	22.3	80	2	1	2	0	0	1	115	74	59	7.4	7.8	7.6	94	10	0.76	179	79	81	86	5	1
1	65	21.0	78	2	1	1	1	0	0	152	93	64	10.5	9.9	10.2	95	15	1.22	151	79	59	85	5.1	0
1	70	26.7	103	2	2	2	1	0	0	148	86	58	10.3	10.5	10.4	112	9	1.02	200	95	97	97	5.6	1
2	59	26.3	83.5	1	1	2	1	0	1	138	88	71	7.2	7.4	7.3	92	17	0.78	317	95	80	218	5.6	1
1	52	23.7	86	2	2	2	0	0	1	114	69	53	7.6	7.7	7.65	92	14	1.04	199	213	37	130	5.6	0
1	54	26.0	93	2	2	2	1	1	0	140	95	62	9.3	9.2	9.25	143	11	0.77	203	111	61	134	5.7	1
1	59	30.5	96	1	1	1	1	0	1	112	79	58	6	5.8	5.9	105	22	1.32	138	58	44	89	5.7	0
1	45	26.1	88	2	2	2	1	0	0	128	94	61	7	7	7	109	18	0.9	172	151	49	110	5.5	1
1	54	29.6	92	2	2	2	0	0	1	130	85	62	7.1	6.7	6.9	103	16	1.05	247	129	70	148	5.9	1
1	57	30.3	103	2	1	2	1	1	1	143	103	66	7.7	7.9	7.8	134	11	0.78	174	202	43	118	6.3	0
1	50	25.4	89.5	2	2	1	0	0	0	120	84	62	7.6	7.2	7.4	96	17	1.08	188	190	41	124	5.4	0
2	66	20.0	77	1	1	1	1	0	1	137	97	89	7.7	7.8	7.75	101	16	0.63	213	51	82	125	6	1
1	66	20.8	77	1	1	2	0	0	1	101	75	53	9.1	9.1	9.1	96	13	1.03	158	83	53	95	5.5	1
1	54	26.1	91.5	2	1	2	0	0	1	109	74	67	6.6	7	6.8	108	17	0.89	272	152	70	177	5.7	1
1	44	29.4	111	2	2	1	0	0	0	127	85	72	7.9	7.5	7.7	91	14	0.9	191	156	43	126	5.4	0
1	47	22.3	82	1	1	2	0	0	0	121	77	65	8.1	7.8	7.95	85	12	0.95	196	107	60	124	5.3	1

1	54	24.7	95	2	2	2	1	0	0	126	93	93	6.6	6.4	6.5	113	11	0.87	181	145	75	86	6.4	0
1	53	26.3	92.5	2	1	2	0	0	1	121	83	59	7.2	7	7.1	94	19	0.91	185	106	40	136	5.4	0
2	51	18.5	65.8	1	1	2	0	0	0	108	58	44	7	7	7	80	10	0.73	177	85	56	107	5.3	0
1	57	23.8	84.5	2	1	1	0	0	0	109	71	43	9	9.2	9.1	91	23	0.88	194	82	50	127	5.1	1
1	45	29.4	103	1	1	1	1	1	0	129	91	71	6.8	6.9	6.85	147	9	1.03	190	109	52	128	7.3	1
1	51	30.2	105	2	2	2	0	0	1	122	84	74	7	6.5	6.75	95	14	0.96	236	126	48	175	5.7	0
1	43	27.8	97	1	1	1	0	0	1	130	83	56	7.1	7.2	7.15	108	14	0.92	250	96	59	181	5.5	0
1	55	27.0	90	1	2	2	1	0	0	129	87	64	7.8	7.7	7.75	122	14	1.18	153	172	41	95	5.9	0
2	50	20.8	78	1	1	2	0	0	1	109	72	59	8.4	8.4	8.4	107	13	0.62	255	156	53	191	5.8	0
1	50	24.1	89.5	2	2	1	0	0	0	127	81	60	7.5	7.2	7.35	99	12	1.02	202	175	48	126	5.4	1
2	64	24.6	88	1	1	1	1	0	1	111	79	66	7.6	7.2	7.4	95	18	0.65	288	135	58	206	5.1	1
1	48	24.9	97.5	2	1	2	1	0	1	134	91	57	6.7	6.6	6.65	88	14	0.9	166	109	51	111	5	1
1	70	26.5	90	2	1	2	1	0	1	119	88	71	8.6	8.8	8.7	104	15	1.02	182	142	36	121	5.7	0
1	55	25.0	89	2	1	1	1	0	0	123	73	61	7.6	7.6	7.6	94	16	0.85	208	129	41	147	5.6	1
2	56	26.6	90	1	1	2	1	1	0	161	94	57	7	6.9	6.95	107	13	0.59	199	140	50	125	6.6	1
1	63	27.1	101	2	1	2	1	0	0	137	88	65	8.3	8.4	8.35	88	11	0.83	192	89	52	124	5.4	0
1	45	23.9	88.5	2	1	1	1	0	0	138	97	60	6.7	6.7	6.7	107	17	0.98	192	102	63	119	5.6	1
1	63	28.7	96	2	2	2	1	1	1	121	84	75	11	10.2	10.6	138	15	0.94	151	95	67	83	5.9	1
2	74	23.8	90	1	1	1	1	0	1	131	79	66	10.5	11.4	10.95	100	18	0.71	263	120	72	164	5.8	0
1	38	26.6	85	2	1	1	1	0	1	152	98	53	5.9	5.8	5.85	95	11	0.93	162	297	35	104	5.5	1
1	51	24.9	90	2	2	2	0	0	0	115	79	58	7.1	7.2	7.15	93	17	0.9	214	104	60	146	5.6	1
1	45	21.3	75.5	1	1	2	0	0	0	120	76	55	6.3	6.1	6.2	91	16	0.94	184	117	44	131	5.8	1
1	71	24.4	87	2	1	2	0	0	0	127	84	58	7.2	6.6	6.9	80	21	0.99	200	87	75	114	5.1	1
1	48	24.3	92	2	2	2	1	1	1	142	99	69	7.3	7.4	7.35	116	11	0.8	158	175	51	86	6.5	1
1	42	24.9	84	2	1	2	0	1	1	121	81	68	6.8	6.5	6.65	136	19	0.86	174	236	52	98	7	0
1	51	25.4	87	2	1	2	1	0	1	118	83	60	8.2	7.9	8.05	95	16	0.94	136	102	32	99	5.6	1
2	50	19.6	74	1	1	2	0	0	1	112	71	61	7.1	7.3	7.2	95	16	0.6	210	67	62	142	5.9	1
1	52	22.0	84	1	1	1	0	0	0	118	83	68	7	6.9	6.95	90	13	0.82	209	138	55	117	5.4	1
1	60	25.6	93	2	1	2	1	0	1	139	94	58	8.1	8	8.05	99	13	1.04	184	251	43	96	5.3	1

2	50	24.8	85	1	1	2	0	1	1	126	82	62	5.9	6	5.95	105	5	0.57	256	210	52	160	5.8	1
1	50	23.9	93	2	1	1	0	1	1	117	78	74	7.6	7.5	7.55	109	16	0.91	146	135	49	82	6.8	0
1	50	24.4	80.5	2	1	2	0	0	0	123	80	43	7.1	7.2	7.15	103	12	0.97	145	49	43	91	5.7	1
1	50	25.0	85	2	2	2	1	1	1	125	83	78	6.7	6.5	6.6	129	18	0.85	215	283	53	109	6.3	0
2	64	22.6	82	1	1	2	1	0	1	146	89	80	8.2	7.8	8	86	20	0.74	229	77	58	160	5.5	1
2	41	20.2	78	1	1	1	0	0	0	111	80	71	7.8	7.6	7.7	88	13	0.68	193	76	56	128	5.2	1
1	51	26.8	99	1	2	2	1	0	0	138	104	55	9.5	8.3	8.9	104	17	1.16	182	84	44	130	5.6	1
1	56	22.6	83.5	2	1	1	1	0	1	130	89	61	7.7	7.5	7.6	94	20	1.07	244	121	74	153	5.5	1
2	52	20.8	77	1	1	1	0	0	0	113	79	70	7	7	7	53	15	0.55	158	26	64	85	5	0
1	44	26.6	91	1	1	2	1	0	0	140	100	70	6.8	6.7	6.75	98	17	0.96	179	120	45	119	5.5	0
1	53	26.0	85	2	1	2	0	0	0	112	74	54	7.5	7.4	7.45	87	15	0.83	176	76	45	117	5.7	0
1	56	28.1	101	2	1	2	0	1	0	110	76	64	7.5	7.4	7.45	121	13	0.88	174	132	46	101	6.6	1
2	53	22.2	75.5	1	1	1	1	0	1	140	78	49	6.9	7	6.95	91	21	0.76	243	103	54	163	5.9	0
1	64	18.7	77	2	1	2	0	1	1	114	78	63	9.5	9.4	9.45	158	22	0.7	167	87	49	109	7.5	0
1	50	23.1	85	2	2	1	0	0	0	117	74	71	8.2	8.1	8.15	94	13	1.02	225	153	68	132	5.3	1
1	43	20.2	77	1	1	1	0	0	0	108	65	69	6.7	6.9	6.8	91	13	0.86	146	36	65	68	5.6	1
1	62	23.8	89	1	1	2	1	0	1	157	89	48	10.7	10.2	10.45	80	10	0.95	149	62	46	98	5.8	1
1	61	26.9	95	1	1	1	1	0	0	160	116	70	8.9	8.6	8.75	95	16	1.06	181	86	47	127	6.1	0
1	52	25.0	89.5	2	2	2	1	0	1	134	90	75	7	7.3	7.15	93	12	1.02	225	153	38	163	5	0
1	54	24.6	91	2	1	1	0	0	0	119	80	54	7.5	7.6	7.55	90	15	0.98	215	168	41	146	5.4	1
1	57	22.5	86	1	2	1	1	0	1	120	78	69	8.8	8.6	8.7	99	17	0.96	231	69	58	170	5.6	0
1	55	25.1	90	2	1	2	1	1	1	115	76	62	6.5	6.7	6.6	118	14	1.05	202	261	42	125	6.2	0
1	51	20.3	76	2	1	2	0	0	0	124	79	59	7.8	7.8	7.8	88	12	0.98	186	91	65	99	5.2	1
1	56	27.1	91	2	2	2	1	0	1	125	79	59	6.1	6.2	6.15	116	16	0.77	163	152	50	96	6.4	1
2	43	19.7	76	1	1	2	0	0	0	116	76	44	7.1	7.1	7.1	98	15	0.69	201	86	63	121	5.3	0
1	53	27.2	92	2	2	2	1	0	0	131	87	60	7.1	7	7.05	86	16	1.11	175	83	52	114	5.3	1
2	50	21.4	81	1	1	2	0	0	1	116	76	67	7.1	7.1	7.1	120	7	0.48	191	205	44	106	6.3	0
1	55	24.6	90	1	1	1	0	0	0	118	82	69	6.6	6.7	6.65	110	12	0.8	196	187	47	127	5.3	0
2	49	24.3	84	1	1	1	0	0	1	120	77	54	8.1	8.5	8.3	91	10	0.62	171	424	36	73	5.4	0

2	58	23.7	90	1	1	1	1	0	1	124	88	60	8.9	8.8	8.85	99	17	0.58	227	104	41	168	5.5	1
2	50	23.2	82.5	1	1	1	1	0	0	116	95	86	6.3	6.3	6.3	108	12	0.65	226	131	56	142	5.5	1
1	49	24.7	86	2	2	2	0	0	1	111	83	69	7.3	6.8	7.05	89	15	0.93	237	136	33	167	5.4	1
2	49	18.7	79.3	1	1	2	0	0	0	113	68	68	7.2	7.3	7.25	94	13	0.55	236	90	68	146	5.4	0
1	48	23.8	82	1	1	1	0	0	0	116	80	58	7.6	7.4	7.5	83	14	1.09	188	178	43	118	5.4	0
1	49	24.4	91	2	2	2	1	1	0	141	102	79	8.7	8.2	8.45	155	19	0.72	165	139	57	99	7.3	1
2	55	23.6	82	1	1	1	0	1	0	103	65	65	6.5	6.4	6.45	112	12	0.72	204	165	55	128	6.5	1
1	43	23.3	85	1	2	2	0	0	0	130	84	63	7	6.8	6.9	96	14	1.2	196	77	66	109	5.5	1
1	55	26.3	94.5	1	2	2	1	0	1	128	89	60	6.8	6.7	6.75	108	12	0.83	201	220	45	133	5.5	0
1	47	24.5	87	1	1	2	0	0	1	107	75	53	6.2	6.2	6.2	98	16	0.91	213	83	66	135	5.4	1
2	48	26.3	106	1	1	2	1	0	0	156	90	73	6.5	6.1	6.3	112	20	0.64	179	109	62	95	6	0
1	51	23.9	84.9	2	1	2	1	0	1	115	77	73	6.9	7.1	7	95	17	1.15	234	124	54	155	5.5	1
1	50	23.1	89.3	2	1	1	1	0	0	128	82	52	7.7	7.3	7.5	105	18	1.1	191	124	44	112	5.8	0
1	54	24.7	87	2	2	2	0	0	1	123	85	67	8.2	8.1	8.15	115	22	0.82	152	79	38	107	4.9	0
1	45	24.2	84.5	2	1	2	0	0	1	123	85	65	7.3	7.2	7.25	87	14	1.03	229	493	38	82	5.2	0
1	41	25.0	81	1	1	2	0	0	0	119	79	63	7	7.2	7.1	96	15	1.17	207	134	54	120	5.8	0
1	53	22.9	84.5	1	2	2	0	0	0	122	78	73	7.6	7.8	7.7	102	13	0.85	217	67	73	128	5.3	1
1	58	22.7	88	2	2	2	0	0	0	116	76	79	6.8	7.2	7	74	11	0.84	169	137	59	85	5.9	1
1	53	24.0	90	2	1	2	1	0	1	106	74	64	7.5	7.5	7.5	109	13	1.06	164	100	62	90	5.7	0
1	47	20.8	82	2	2	2	0	0	0	108	77	64	8.6	8.2	8.4	85	11	0.71	172	44	75	86	5.1	1
1	35	33.8	113	2	1	1	1	1	1	165	104	66	7.3	7.3	7.3	147	17	1.23	195	328	44	105	9	0
2	44	22.4	79	1	1	1	0	0	0	120	84	64	7.6	7.7	7.65	93	9	0.61	182	128	40	118	5.1	0
1	42	24.8	88.2	2	2	2	1	0	0	122	77	63	6.5	6.6	6.55	101	9	0.86	222	145	50	153	5.3	0
1	58	27.0	98.5	2	2	1	1	1	1	137	91	74	8.3	8.3	8.3	120	16	0.74	149	87	52	78	6	0
1	49	31.4	101	2	1	2	1	0	0	137	80	64	7.2	7.2	7.2	98	14	1.13	177	177	47	100	5.4	1
1	39	25.1	92.2	2	1	2	0	0	1	114	82	66	8.3	8.1	8.2	89	18	0.84	169	209	32	119	4.8	1
1	60	22.9	86.9	1	1	2	0	0	0	129	77	59	8.3	8.2	8.25	93	14	0.86	165	83	85	124	5.1	0
1	56	23.2	84	1	2	2	1	0	0	138	88	63	6.2	5.8	6	76	15	1.2	127	90	43	74	5.1	0
2	52	20.5	69	1	1	1	1	0	1	112	79	55	7.5	7.8	7.65	89	19	0.64	197	65	86	105	5.6	0

1	48	24.8	88	2	2	2	0	0	1	116	85	72	6.8	7.2	7	110	19	0.95	166	113	37	114	5	1
1	56	22.7	85.8	2	1	1	0	0	1	122	86	60	6.9	6.9	6.9	93	11	0.71	182	85	61	116	5.2	1
2	52	24.1	82	1	1	2	0	0	1	123	78	68	6.9	7	6.95	112	11	0.6	203	264	39	124	5.9	0
1	50	24.2	81.5	2	1	1	1	0	1	127	91	73	9.3	8.3	8.8	91	20	1.18	224	77	51	174	5.4	1
1	47	26.5	91	2	1	2	0	1	0	129	81	70	7.2	7.2	7.2	129	11	0.78	156	146	42	98	6.5	1
1	46	25.4	85	2	1	2	0	0	0	133	87	65	6.9	6.7	6.8	91	11	0.81	169	104	53	101	5.4	1
1	52	24.8	86.6	1	1	1	1	0	0	140	89	67	6.7	6.5	6.6	96	18	0.72	172	114	44	120	4.8	0
1	48	27.4	91.5	1	1	2	0	0	1	115	78	55	6.6	6.7	6.65	113	19	1.16	237	112	57	168	5.6	1
1	44	29.2	101.6	2	2	1	0	0	1	121	75	50	7.8	7.5	7.65	118	18	0.88	223	230	43	146	6.1	1
1	50	25.6	89	1	1	1	1	0	0	134	97	64	10.8	7.3	9.05	116	12	1.01	189	164	45	134	6	1
1	69	20.6	79.5	1	1	1	1	1	1	111	70	94	9.7	9.7	9.7	92	13	0.64	150	71	56	85	5.5	1
1	61	22.8	84	1	1	1	1	0	1	122	75	79	7.4	7.3	7.35	96	16	1.06	197	188	41	124	5.3	1
1	43	22.7	88.8	2	1	1	1	0	1	140	100	70	8.4	8.2	8.3	123	14	0.93	194	101	33	148	5.8	1
1	61	25.1	85.5	2	1	1	0	0	0	134	88	69	9	9.1	9.05	79	13	1.06	191	73	53	134	4.9	1
1	48	25.2	87.8	2	1	1	0	0	0	118	80	62	7.3	7.1	7.2	88	14	1.02	229	150	53	156	5.5	1
2	61	26.8	85.5	1	1	1	0	0	1	119	79	78	8.5	8.1	8.3	106	15	0.57	238	114	49	170	5.5	0
1	59	25.7	89	2	1	2	1	0	1	130	89	74	7.7	8	7.85	103	14	1.16	232	115	46	172	5.3	1
1	58	29.4	95.3	1	1	2	1	0	1	124	83	50	7.1	7.1	7.1	102	14	1.15	227	137	46	166	5.4	0
2	53	17.2	68	1	1	2	1	0	0	151	92	68	8.1	8.1	8.1	88	14	0.84	178	96	53	113	5.5	0
2	58	21.0	76	1	1	1	1	0	0	123	81	76	7	7.1	7.05	102	15	0.53	181	46	52	122	6.1	0
1	50	24.0	82	1	1	1	0	0	0	123	79	68	6.3	6.5	6.4	94	13	0.93	174	63	56	97	5.4	0
1	49	21.5	81	2	1	2	0	1	0	113	76	66	7.9	7.9	7.9	164	24	0.82	136	76	52	73	9.4	1
1	48	24.7	95	2	1	1	0	0	1	128	88	60	7	6.8	6.9	96	15	0.89	200	235	40	132	5.5	1
1	50	24.4	89	1	1	1	1	0	1	130	96	76	7.4	7.3	7.35	98	11	0.99	217	156	49	146	5.5	0
1	44	20.4	73	2	1	2	0	0	1	112	80	75	8.1	8.3	8.2	97	11	0.84	170	95	37	117	5.7	1
2	50	20.8	75.5	1	1	2	0	0	0	115	67	64	7.4	7.6	7.5	95	10	0.77	215	95	64	126	5.5	1
2	42	19.7	77.5	1	2	1	0	0	0	117	78	56	7.8	7.5	7.65	85	14	0.65	206	73	65	137	5.2	0
1	48	24.1	83	2	2	2	0	0	1	117	74	67	6.4	6.4	6.4	95	10	1.01	174	55	53	125	5.3	1
1	54	24.8	84	2	1	2	0	0	0	123	87	68	7.4	7.5	7.45	110	17	1.09	224	108	42	156	5.8	1

1	47	21.5	83	2	2	2	0	0	0	123	75	58	6.6	6.5	6.55	97	13	0.84	168	102	51	95	5.2	0
1	46	23.0	78	2	2	2	0	0	0	119	75	64	7.2	7.3	7.25	95	15	0.92	183	101	48	128	5.5	1
1	53	24.3	90.5	1	1	2	1	0	1	132	90	68	7.2	7.5	7.35	94	12	0.84	140	52	48	83	5.6	0
1	58	23.3	81	2	1	2	0	0	0	128	82	60	7.3	7.8	7.55	95	10	0.86	185	78	69	111	5.6	0
1	48	25.7	92	2	1	2	0	0	1	131	89	83	6.4	6.3	6.35	109	15	0.78	199	237	35	145	6.4	0
1	54	26.6	92.5	2	2	2	0	0	0	124	82	66	6.9	6.9	6.9	93	20	1.06	154	96	43	94	5.4	1
1	57	26.2	97	2	1	1	1	0	1	132	97	82	8.7	10.1	9.4	106	13	0.95	243	174	71	144	5.7	1
1	48	26.1	96.5	2	2	2	0	0	1	137	87	50	8.9	8.8	8.85	84	14	0.86	223	189	60	134	5.6	0
1	46	23.1	83	1	1	2	1	1	1	145	108	71	8.4	10.6	9.5	227	11	0.77	299	317	50	195	9.3	1
2	53	21.0	71	1	1	2	0	0	0	113	77	61	7.7	7.6	7.65	91	13	0.65	196	150	59	110	5.6	1
1	57	24.0	88.3	2	2	2	1	0	0	114	74	83	7.6	7.5	7.55	110	12	0.95	206	152	62	130	5.4	1
1	46	28.4	95	2	1	1	1	0	1	151	96	70	6.6	6.4	6.5	122	11	1.05	177	137	57	100	5.8	0
1	43	22.3	84.5	2	1	2	0	0	0	125	89	49	7.2	7.2	7.2	101	13	0.72	170	122	50	110	5.5	1
1	57	29.8	100	2	1	2	1	0	1	117	72	58	8.1	8.2	8.15	107	24	1.05	178	106	49	120	6.1	0
2	42	26.3	91	1	1	1	1	1	1	141	107	64	7.9	7.4	7.65	195	14	0.59	155	332	35	77	9.1	0
1	42	25.8	88	2	1	2	0	0	1	123	80	62	7.1	6.8	6.95	107	19	0.84	211	298	43	118	5.6	1
1	54	28.7	92	1	1	2	1	1	1	142	82	71	7.2	7.3	7.25	105	14	0.8	153	84	44	101	5.9	0
1	60	26.1	93	2	1	2	1	0	1	124	88	56	8.7	8.7	8.7	104	16	0.94	188	186	44	119	5.8	1
1	53	25.4	94	1	1	1	1	0	0	126	83	61	6.8	6.8	6.8	88	10	0.87	218	88	55	139	5.4	0
1	46	25.8	87	2	1	2	0	0	1	104	62	56	7.1	7.1	7.1	110	12	0.73	213	134	47	136	5.8	0
1	63	23.0	83	2	1	2	0	0	1	111	80	70	9	9.1	9.05	89	18	1.25	197	84	55	119	6	1
1	60	25.4	87	2	2	2	1	0	1	152	101	67	9.5	9.4	9.45	84	13	0.81	175	337	30	91	5.6	1
1	60	25.0	92	1	1	2	0	0	1	123	87	55	8	7.9	7.95	88	10	0.86	189	425	34	92	5.2	1
1	53	23.4	89	2	1	1	0	0	1	114	78	78	7.5	7.7	7.6	92	17	0.83	167	105	39	164	6.1	1
1	54	24.9	86	2	1	2	1	0	1	139	98	79	8.3	8.2	8.25	105	11	0.94	183	263	33	110	5.7	1
1	49	27.5	95	1	1	2	1	0	1	143	94	65	7.8	7.6	7.7	104	12	1.01	219	161	48	150	5.3	1
1	59	20.9	83	2	1	2	1	1	0	128	90	91	8.6	8.6	8.6	153	15	0.86	206	179	44	139	7.1	1
2	56	22.4	77	1	1	1	1	0	0	119	78	53	9	8.9	8.95	101	16	0.83	152	67	64	88	6	1
1	59	25.6	91	1	1	2	1	0	1	140	101	69	8.6	7.9	8.25	99	12	1	212	204	41	126	5.4	1

1	57	21.9	81	1	1	2	1	0	0	167	100	46	8.2	7.6	7.9	102	13	0.89	170	47	68	92	5.5	0
1	49	24.5	86.3	1	1	2	0	0	1	117	74	56	7.1	7.1	7.1	105	12	0.97	208	112	37	147	5.7	1
1	51	22.5	84.5	1	1	2	0	0	0	134	83	64	7	7	7	92	11	1.01	182	106	52	119	5.5	1
1	69	23.8	85	2	1	2	0	0	1	132	87	69	10.9	10.7	10.8	107	17	0.92	158	108	54	90	5.7	1
1	53	20.5	76	1	1	2	0	0	0	128	84	56	7.3	7.4	7.35	92	15	0.86	159	51	62	84	5.4	0
1	53	22.1	80	1	1	2	0	0	0	116	75	59	7.3	7.4	7.35	101	17	0.8	207	74	53	140	5.5	1
1	54	22.6	77.5	1	1	2	0	0	0	124	89	74	6.4	7	6.7	87	22	0.83	174	40	63	94	5.4	1
1	51	26.5	98.5	2	1	1	1	0	1	138	97	65	6.7	6.9	6.8	111	14	0.99	242	193	59	151	5.7	0
1	52	25.6	95	2	2	1	1	0	0	131	97	48	7.6	7.4	7.5	121	15	0.78	227	102	60	150	5.9	1
1	57	31.3	107	1	1	2	0	1	1	135	86	75	8.4	8.3	8.35	129	14	1.04	187	292	32	110	4.3	1
1	52	22.7	85	2	1	2	0	0	0	120	78	59	7.4	7.2	7.3	93	15	0.97	204	44	69	135	5.4	0
2	58	29.4	100	1	1	1	1	0	1	141	83	68	8.3	8.3	8.3	104	20	0.61	224	378	41	105	5.7	0
1	47	30.0	100	2	2	1	1	0	1	143	100	62	7.1	7.1	7.1	117	12	0.91	328	308	32	217	6.3	0
1	51	26.5	92	2	2	1	0	0	0	138	84	64	10.9	11.6	11.25	108	11	0.95	198	159	55	124	5.8	0
2	55	22.5	76	1	1	2	0	0	1	129	89	65	6.8	6.2	6.5	93	12	0.57	197	84	68	110	5.5	0
1	43	25.6	87	1	1	1	1	0	0	140	89	51	6.5	6.3	6.4	93	13	1.05	225	84	54	150	5.3	1
1	40	22.9	86.5	1	1	1	0	0	0	130	89	88	6.7	6.8	6.75	89	16	0.88	180	58	49	124	4.9	0
1	49	22.1	80	2	1	2	0	0	1	124	87	58	7.4	7.6	7.5	105	23	1.02	253	68	52	179	5.4	0
1	45	23.6	90.5	2	2	2	0	0	1	114	72	59	7.4	7.4	7.4	103	12	0.83	171	84	37	126	5.4	0
2	76	22.3	87	2	2	2	1	0	0	146	75	61	8.5	8.3	8.4	101	24	0.69	212	59	78	130	5.8	0
1	44	24.8	89	1	1	2	1	0	0	129	94	69	6.9	6.9	6.9	91	14	1.12	231	130	64	129	5.6	1
2	51	23.1	74	1	1	1	0	0	0	122	80	67	7.5	7.4	7.45	95	10	0.75	186	75	40	133	5.4	0
1	50	23.3	83	2	2	2	0	0	0	120	78	52	7.7	7.5	7.6	90	10	0.91	224	62	67	153	5.6	1
1	52	25.4	89	2	1	1	1	0	1	137	98	67	6.7	7.3	7	120	13	0.9	127	381	45	44	5.9	1
1	55	24.2	88.5	2	1	2	1	0	0	145	101	81	7.7	7.9	7.8	87	10	0.7	172	44	67	91	4.9	1
1	39	16.5	67.5	2	1	2	0	0	0	121	81	62	7.8	7.5	7.65	84	13	0.93	182	66	87	98	5.6	1
1	52	27.4	91	2	2	1	1	1	1	137	83	73	7	7.1	7.05	147	30	0.78	129	252	47	46	6.8	1
1	51	24.4	92	2	1	2	0	0	1	108	77	69	8.4	8	8.2	86	15	0.91	246	137	48	175	5.6	1
1	55	24.8	89	1	1	1	0	0	1	122	85	67	7.9	8.1	8	113	12	1	150	97	51	96	6	1

1	63	26.3	93.5	2	1	1	1	0	1	124	80	78	11.4	7.5	9.45	101	10	0.82	160	60	55	103	6.1	1
1	41	29.4	98	1	1	2	1	1	1	136	92	83	7.2	6.9	7.05	311	14	0.73	208	193	39	143	13.2	1
2	52	20.4	78	1	2	1	0	0	0	119	76	56	7.7	8.1	7.9	98	9	0.63	205	72	58	137	5.5	1
2	43	22.1	73	2	1	2	1	0	0	135	91	61	5.8	5.8	5.8	84	7	0.53	172	134	53	98	5.3	0
1	35	22.7	79	2	1	2	0	0	1	139	89	60	6.5	6.5	6.5	91	8	0.95	137	89	27	101	5.4	1
1	42	24.7	91	2	1	1	0	0	1	115	75	59	7.8	7.6	7.7	84	15	0.82	227	124	45	163	5.4	0
1	73	23.8	81	2	1	2	1	0	1	178	84	62	12.3	12.5	12.4	105	15	0.84	130	167	51	63	5.6	0
1	54	22.4	83.5	2	1	2	1	0	0	125	79	62	8.7	8.5	8.6	93	11	1	171	63	50	115	5.4	1
1	55	23.8	80	2	1	2	0	0	1	132	87	52	8.2	8.4	8.3	100	17	1.18	218	75	70	137	5.4	1
1	56	26.1	91	1	1	2	0	0	0	116	78	72	6.4	6.6	6.5	99	18	0.93	178	69	43	127	5.8	1
2	45	20.0	70	1	1	1	0	0	0	110	74	49	7.4	7.5	7.45	94	12	0.77	216	82	56	148	5.3	1
1	48	21.1	78	1	1	2	0	0	1	133	82	66	7.2	7.2	7.2	92	13	1.09	198	57	56	129	5.4	0
1	52	24.9	85	2	2	2	1	0	0	147	99	71	7.7	7.7	7.7	113	19	0.84	167	161	43	108	5.3	1
2	54	27.7	94	1	1	2	1	0	1	136	92	68	8.5	10.4	9.45	114	11	0.65	224	106	40	168	5.7	0
1	52	26.6	94	2	2	2	0	0	0	138	86	71	6.3	6.3	6.3	97	12	0.97	219	136	60	147	5.9	0
1	42	25.7	90	2	1	2	1	0	0	136	92	68	7.5	7.6	7.55	98	10	0.87	186	134	43	134	5.6	0
2	60	28.3	88	1	1	1	0	0	0	120	73	62	6.5	6.4	6.45	102	13	0.74	216	78	57	145	5.5	1
1	51	22.2	83	2	2	2	0	0	0	123	72	53	7.5	7.3	7.4	98	7	0.93	225	101	61	140	5.4	0
1	51	27.1	85.5	2	1	1	0	0	0	136	89	89	6.9	7	6.95	125	11	1	205	57	99	110	6.2	0
2	46	22.8	78.5	1	1	1	0	0	0	104	58	65	6.2	6.5	6.35	76	12	0.74	164	43	55	100	5	1
1	46	22.9	84	2	1	2	0	0	0	119	80	61	7.4	7.4	7.4	91	13	0.9	178	99	58	99	5.4	0
1	52	19.8	80	2	1	2	1	0	0	131	90	74	7.8	7.5	7.65	98	14	1.07	184	91	46	126	5.5	1
2	55	23.1	83.5	1	1	2	0	0	1	127	77	64	6.3	6.1	6.2	91	13	0.58	230	180	45	163	5.7	1
1	65	22.9	83	2	2	1	1	1	1	178	104	83	10.7	9.8	10.25	267	14	0.76	241	110	73	161	8.8	1
1	51	22.6	80	2	1	2	0	0	1	121	86	73	10.2	8.3	9.25	97	14	0.9	190	81	55	129	5.9	1
1	49	38.4	120	2	1	2	1	1	1	166	92	91	7.3	6.8	7.05	220	10	0.83	129	146	36	83	9.9	0
2	54	23.9	86	1	1	1	0	0	1	127	79	68	6.9	7	6.95	104	16	0.67	240	60	73	156	5.8	0
1	43	20.5	75.5	2	1	2	0	0	0	131	84	76	7.4	7.3	7.35	103	14	1.04	234	143	63	137	5.4	0
1	32	24.2	90	1	1	2	1	0	0	131	93	71	7.4	7.3	7.35	110	11	0.83	174	189	49	101	5.3	1

1	41	19.4	76.5	2	1	1	0	0	0	115	76	77	7.9	7.7	7.8	105	18	1.06	187	175	43	127	5.8	0
1	38	27.7	92	2	1	1	0	0	1	129	77	56	6.7	6.8	6.75	97	9	0.83	246	145	53	173	5.4	1
1	33	26.6	96.5	2	2	1	0	0	0	131	73	51	6.8	6.7	6.75	97	15	0.94	200	105	43	144	5.7	1
1	56	20.9	75	1	1	2	0	0	0	112	82	70	8.7	8.9	8.8	100	15	0.97	203	73	69	117	5.4	1
1	48	23.6	85.6	2	1	2	0	0	0	132	87	62	7.8	7.8	7.8	93	16	1.04	223	89	62	146	5.4	0
1	63	22.7	87	2	1	1	1	1	1	153	100	53	10.8	10.2	10.5	168	10	0.64	162	107	69	87	7.8	1
1	52	23.5	84	2	1	1	0	0	1	107	67	62	7.6	7.7	7.65	93	16	0.81	204	241	52	128	5.5	1
1	52	22.5	87	1	2	1	0	1	0	130	85	65	9	8.1	8.55	147	15	0.91	194	98	45	134	6.6	1
1	53	30.7	102	1	1	1	1	0	1	175	109	99	7.8	7.2	7.5	113	12	0.9	177	105	37	127	5.9	1
2	43	22.8	78	1	1	1	0	0	0	109	70	60	6.9	7	6.95	88	15	0.68	146	42	45	91	5.8	0
2	31	20.8	79.2	1	1	1	0	0	0	112	75	68	6.6	6.5	6.55	95	13	0.67	224	46	56	151	5.4	1
1	53	37.9	120	2	2	1	1	0	0	173	117	83	8	7.8	7.9	115	10	0.89	210	176	53	145	5.8	1
1	52	27.7	98	2	1	1	1	0	0	148	98	64	9.4	7.5	8.45	87	13	1.02	189	126	54	120	5.4	0
1	26	24.8	80.5	2	1	2	0	0	0	123	68	64	6.2	6.2	6.2	75	19	1.05	177	96	56	118	5.2	0
1	43	26.7	89	2	1	2	1	0	1	155	94	64	6.6	6.6	6.6	99	18	1.01	140	137	38	55	5.4	0
1	52	27.0	93	2	1	2	1	0	0	133	92	59	7.7	7.8	7.75	100	13	0.8	196	109	47	135	5.5	1
1	51	21.6	81.5	1	1	2	1	0	1	156	102	59	10.7	8.6	9.65	103	14	0.97	179	211	36	114	5.4	1
1	67	24.5	90	2	1	2	1	0	0	128	89	73	9.3	9	9.15	123	19	0.97	200	143	65	122	5.8	1
2	45	19.6	73	1	1	1	0	0	0	114	72	61	7.4	7.5	7.45	88	13	0.62	146	44	59	76	5.4	1
2	36	19.1	70	1	1	1	0	0	0	102	72	62	6.6	6.7	6.65	85	10	0.48	159	39	50	92	5.2	1
2	30	22.5	79.5	1	1	1	0	0	1	115	67	52	8.2	8.2	8.2	91	13	0.73	165	187	34	101	5.1	1
2	47	23.9	78.5	1	1	1	0	0	0	122	82	52	5.8	5.6	5.7	86	14	0.76	199	165	49	133	5.4	0
1	57	22.1	84	1	1	2	1	1	0	124	91	73	8.1	7.2	7.65	102	11	0.78	143	70	49	92	6.3	1
2	54	22.3	85	1	1	1	0	0	0	114	70	49	8.1	8.2	8.15	90	13	0.63	177	63	46	126	6	1
1	47	23.7	84	2	1	2	0	0	0	120	85	60	7.5	7.6	7.55	81	13	1.26	177	92	43	122	5.6	1
2	45	27.1	94	2	2	2	1	0	0	150	99	59	7.1	6.4	6.75	104	17	0.59	181	59	81	104	5.8	1
1	52	23.8	86	2	1	2	1	0	1	120	90	65	7.7	8.1	7.9	113	13	1.17	153	87	55	90	5.8	0
2	61	22.9	78	1	1	2	1	0	1	149	85	64	10.2	9.8	10	85	9	0.54	165	67	39	108	5.1	0
1	64	23.8	90	2	1	2	1	0	0	127	84	53	8.1	8.4	8.25	102	19	1.12	202	166	49	137	5.6	1

1	60	30.1	105	2	2	1	1	0	0	159	106	83	9.8	9.6	9.7	118	7	0.61	230	166	65	132	5.6	1
2	41	24.5	76	1	1	1	1	1	1	154	111	67	7.7	6.9	7.3	174	13	0.6	163	200	43	101	7.9	1
1	44	24.8	94	2	2	1	0	0	0	116	80	65	6.9	6.7	6.8	97	19	0.91	194	183	46	126	5.4	0
1	55	28.4	94	2	1	2	1	0	1	124	89	55	8.6	8.2	8.4	99	19	1.09	198	181	39	140	5.6	1
1	46	26.0	88	2	1	2	1	0	1	127	85	79	7.4	7.9	7.65	96	13	1.07	223	147	53	156	5.5	0
1	45	29.1	94	2	2	2	0	0	0	109	73	56	7	7.3	7.15	102	9	1.07	179	90	47	118	5.5	0
1	60	26.5	106.5	2	2	1	1	0	1	151	97	64	7.8	7.7	7.75	101	14	0.68	240	95	41	177	5.7	1
1	50	23.3	95	2	1	2	0	0	0	138	95	62	8.4	8.3	8.35	95	8	0.97	205	99	57	135	5.4	1
1	51	29.1	100.5	2	1	2	0	0	1	111	78	53	7.5	7.4	7.45	93	17	0.98	205	149	28	130	5.4	1
1	53	24.7	97	2	2	1	0	0	0	121	83	82	8.4	8.1	8.25	118	13	0.86	168	91	42	115	5.6	0
1	51	25.5	88	2	2	1	1	0	0	134	101	61	7.1	7.4	7.25	109	13	0.82	185	191	40	103	5.5	1
1	68	23.4	88	1	1	2	0	0	0	133	88	79	6.8	7	6.9	92	19	1.06	187	48	57	111	5.4	1
1	48	29.0	95	2	1	2	1	0	1	142	105	80	6.7	7	6.85	110	14	1.04	231	340	51	113	5.8	1
1	45	26.1	90	2	1	1	0	1	1	124	78	77	6.6	6.8	6.7	133	14	0.72	192	438	27	90	7.6	0
1	44	29.7	103	2	2	2	0	0	1	126	84	58	6.7	6.7	6.7	91	11	1.02	241	67	55	161	5.4	1
1	52	24.0	84	2	1	1	0	1	1	126	84	71	8.4	8.3	8.35	149	14	0.95	166	129	57	92	7	1
1	46	24.4	91	2	1	1	0	1	1	131	88	69	6.9	6.9	6.9	142	14	0.9	258	91	43	180	6.8	1
1	48	23.3	83	2	2	2	1	0	0	116	81	61	7.1	7.2	7.15	96	14	0.91	178	66	71	96	5.4	1
1	48	23.3	79.5	2	1	1	0	0	0	116	70	67	7.4	7.5	7.45	101	10	0.93	209	97	49	138	5.5	1
1	49	36.8	116	2	2	2	1	1	1	132	85	83	6.9	7.3	7.1	111	17	1.31	239	258	39	145	7.3	1
1	56	21.7	80	1	1	1	1	1	0	139	93	65	8.3	8.2	8.25	127	17	1.05	232	124	70	152	5.4	1
1	58	24.3	90	2	1	2	0	1	1	136	79	54	7.5	7.4	7.45	102	17	0.89	214	181	37	144	6.7	0
1	31	28.7	98	2	2	2	0	0	1	127	85	62	7.3	6.9	7.1	110	11	0.81	222	289	51	134	5.4	0
2	56	21.4	76	1	1	2	0	0	0	103	72	54	7.3	7.3	7.3	90	12	0.64	199	90	67	123	5.5	0
2	53	21.3	76	1	1	2	0	0	0	129	89	62	7.2	7.6	7.4	99	13	0.68	144	62	63	76	6.1	0
1	39	22.4	80.5	1	2	2	1	0	0	133	94	62	7.1	6.8	6.95	94	11	0.84	179	38	90	93	5.3	1
1	42	21.1	77	2	1	2	0	0	0	109	74	62	6.8	6.8	6.8	105	11	0.92	203	46	74	120	5.3	0
1	49	28.1	97	2	2	1	0	0	0	127	83	61	7.4	7.3	7.35	105	15	1	216	174	46	154	5.7	1
1	47	26.8	94	2	2	1	1	1	1	127	98	81	9.2	7.7	8.45	162	12	0.94	250	261	45	176	7.4	0

1	49	22.2	84	1	2	2	1	0	1	130	91	70	7.4	7.4	7.4	97	19	0.85	254	122	58	180	5.6	0
1	42	25.7	88	2	2	2	1	0	0	125	91	53	7.5	7.7	7.6	98	19	1.05	180	176	42	113	5.2	0
2	59	21.3	83	2	2	2	0	0	0	134	87	78	7.8	7.4	7.6	100	17	0.68	209	112	49	142	5.8	1
1	51	25.9	92	2	2	2	1	0	0	120	88	82	6.3	6.5	6.4	88	13	1.16	235	151	85	139	5.3	1
1	49	23.1	86	1	1	2	0	0	0	124	79	65	7.7	8	7.85	91	18	1.04	198	67	60	121	5.5	1
2	44	20.9	74.5	1	1	1	0	0	0	107	68	77	6.8	6.9	6.85	87	15	0.78	213	36	86	120	5.3	0
2	69	24.1	82	1	1	1	1	0	1	135	84	54	9.4	9.6	9.5	101	18	0.75	215	219	56	120	5.9	1
1	45	23.8	89	2	1	2	0	0	1	122	86	69	6.9	6.9	6.9	100	11	1.1	238	158	61	150	5.4	0
2	58	21.3	76	1	1	2	0	0	0	117	79	54	7.4	7.6	7.5	103	13	0.7	221	115	55	143	5.6	0
1	49	23.0	84	1	1	2	0	0	1	120	79	63	7.5	7.4	7.45	103	12	0.84	247	92	48	178	5.5	0
1	47	24.3	93	1	1	1	0	0	1	134	89	61	6.8	6.9	6.85	98	18	0.91	193	252	50	103	5.6	1
1	51	27.4	93	1	1	1	0	0	1	127	84	58	6.8	7	6.9	107	17	1.07	161	171	53	82	5.8	0
1	51	25.6	89	1	2	1	0	0	0	123	87	67	6.6	6.7	6.65	80	15	0.94	176	90	46	118	5.6	1
1	46	23.3	87	2	1	1	1	0	1	141	93	74	7.6	7.4	7.5	120	16	0.94	256	268	59	156	5.6	1
1	50	25.0	91	2	1	1	1	0	1	149	96	45	7.6	8	7.8	93	19	0.85	222	114	74	132	5.9	1
1	61	23.6	81	2	1	1	0	0	0	111	73	47	7.2	6.9	7.05	77	13	0.77	168	63	48	106	5.1	0
2	61	20.2	76	1	1	1	0	0	0	114	75	81	7.5	7.5	7.5	103	14	0.72	214	53	81	120	5.7	1
2	57	22.4	76	1	1	2	0	0	1	117	80	65	8.8	8.7	8.75	109	20	0.72	243	125	66	143	5.9	1
1	43	25.3	98	1	1	2	0	0	0	138	88	72	6	5.9	5.95	90	14	1.03	181	72	45	122	5.4	0
1	57	25.5	94	2	2	2	1	0	1	128	88	61	7.1	7.1	7.1	109	10	0.67	188	140	39	130	5.6	1
1	58	24.1	101	2	1	1	0	0	1	129	96	56	8.4	8.2	8.3	105	12	0.83	250	72	59	163	5.4	1
1	36	25.2	94	2	1	2	0	0	0	131	89	67	5.7	5.8	5.75	85	12	1.08	170	52	64	98	5	1
1	49	33.9	107	2	1	2	1	0	1	145	90	61	5.9	5.6	5.75	86	12	1.08	172	199	38	116	5.5	1
1	52	23.0	95	2	2	2	1	0	0	132	91	74	6.9	6.8	6.85	119	18	0.98	183	115	59	96	5.8	0
1	46	23.6	82.5	2	2	2	1	1	1	141	92	69	7.4	7.4	7.4	137	14	1.04	228	176	47	144	6.2	0
1	53	23.3	90	2	1	1	1	0	1	138	94	89	8.3	8.4	8.35	114	12	0.92	154	180	47	93	5.6	1
1	63	23.6	88	2	2	2	1	1	1	150	97	72	9.9	9.6	9.75	129	15	0.98	220	285	43	119	6.8	1
1	51	27.7	94	2	1	2	1	0	1	133	91	50	8.2	7.8	8	96	19	0.94	181	104	39	131	5.6	1
1	54	25.8	89	2	2	2	1	0	0	119	88	61	7.9	8	7.95	105	15	0.68	205	101	52	136	5.5	0

1	52	22.7	89	2	2	2	1	1	1	146	97	63	6.8	7	6.9	158	13	0.98	178	173	65	92	6.6	0
2	60	21.4	73	2	2	2	1	1	1	106	69	68	9.3	8.9	9.1	128	18	0.82	171	76	102	71	6.1	1
2	56	26.1	90	1	1	1	0	0	0	124	82	76	6.7	7	6.85	84	11	0.7	227	54	66	147	5.4	1
2	53	19.8	75.5	1	1	2	1	0	1	143	94	80	8	7.9	7.95	103	14	0.58	249	71	61	172	5.4	1
2	56	23.7	83	1	1	1	0	0	1	117	72	74	9.1	8.1	8.6	87	10	0.67	231	680	30	96	5.4	1
2	53	20.3	73	1	1	2	0	1	1	133	78	71	7.9	8.2	8.05	113	10	0.61	170	47	79	93	6.2	1
1	61	24.3	94	2	1	2	1	0	0	140	98	59	8.4	8.1	8.25	99	15	0.84	169	153	42	107	5.7	1
1	55	23.2	84	2	1	2	0	0	0	130	83	56	7.6	7.6	7.6	98	11	0.85	177	183	41	116	5.3	1
1	51	25.3	87	2	2	2	1	0	1	151	104	63	8.5	8	8.25	105	19	1.06	176	219	39	114	5.7	1
2	42	28.9	95	1	1	1	0	0	0	130	71	59	6.6	6.6	6.6	100	11	0.69	181	41	63	109	5.4	1
2	52	26.4	87	1	1	1	0	0	1	135	77	76	6.1	6.1	6.1	100	12	0.57	238	110	51	163	5.4	1
1	56	29.3	93.6	2	2	2	0	0	0	118	80	64	7.5	7.4	7.45	98	13	0.89	168	121	43	114	5.4	1
2	57	22.2	79	1	1	2	1	0	0	136	84	73	7.9	7.9	7.9	77	12	0.65	227	102	62	93	5.6	1
1	57	27.3	94	2	2	1	1	1	1	149	104	66	7.3	7.3	7.3	126	13	0.79	217	175	53	138	6.3	1
1	49	25.2	97	2	2	1	0	0	1	114	77	65	6.4	6.4	6.4	82	20	0.96	207	163	37	135	5.4	1
1	59	25.6	95	2	1	1	1	0	0	132	89	73	9.9	9.8	9.85	100	15	0.6	159	114	54	141	5.5	1
2	52	21.6	75	1	1	2	0	0	1	113	80	55	7.6	7.7	7.65	95	16	0.7	310	193	48	191	5.9	1
1	59	21.4	81	2	1	2	0	0	0	127	85	53	8.3	8.4	8.35	105	15	0.97	239	130	59	145	5.7	1
1	69	23.1	88	2	2	1	1	0	0	137	90	63	7.3	7.2	7.25	89	14	1.41	229	50	74	135	5.2	1
2	48	23.5	85	2	1	2	0	0	1	111	80	57	8.6	8.3	8.45	104	11	0.7	250	220	55	134	5.7	1
1	62	24.9	84	1	1	2	0	0	1	127	85	58	7.1	7.4	7.25	100	13	1.08	256	68	73	154	5.6	1
1	70	28.3	104	2	2	2	1	0	0	155	97	61	11.2	10.6	10.9	100	14	1.06	190	46	46	131	6.1	1
2	64	26.8	101	2	1	1	1	0	0	139	94	64	8.4	8.5	8.45	102	16	0.89	211	81	44	140	6.4	1
2	66	22.9	79	1	1	2	0	0	0	132	82	48	8.9	8.4	8.65	99	18	0.89	232	89	53	158	5.6	1
1	50	21.7	82	2	1	1	1	0	0	151	101	57	6.6	6.7	6.65	94	14	0.79	193	97	62	113	5.4	1
2	60	19.0	75	1	1	2	0	0	1	133	82	65	8.6	8.4	8.5	116	10	0.66	268	163	48	159	5.6	1
1	59	19.8	77	2	2	1	1	1	0	143	91	88	10.2	9.2	9.7	164	11	0.84	108	94	40	44	7.5	1
1	57	22.3	78	2	2	2	0	0	0	114	74	53	8.1	8	8.05	102	16	0.97	171	64	70	104	5.5	1
1	56	22.7	78.5	2	1	1	0	0	0	128	82	54	6.9	6.9	6.9	108	18	0.74	226	73	78	126	6.1	1

1	75	29.8	102	2	1	2	1	0	1	128	84	55	8.5	8.4	8.45	98	10	0.75	158	112	48	145	5.8	1
1	56	28.7	97	2	2	2	1	0	1	136	102	88	7.4	7.5	7.45	113	13	0.99	237	134	46	172	5.8	1
1	53	27.5	88	1	1	1	1	0	1	133	97	70	8.3	7.5	7.9	92	17	1.16	174	110	35	124	5.6	1
1	52	28.4	94	2	1	2	0	0	1	137	87	67	7.7	7.6	7.65	94	15	1	193	186	43	131	5.7	1
1	63	28.7	103	2	1	2	1	0	1	144	92	68	7.9	7.7	7.8	105	13	0.85	182	340	39	110	5.6	1
2	54	24.0	85	1	1	2	0	0	1	130	79	56	7.2	7.4	7.3	87	12	0.8	264	72	63	186	5.7	1
1	68	24.8	84	1	1	1	0	0	1	130	78	83	6.5	6.2	6.35	96	16	1.06	260	135	42	167	6.1	1
1	51	26.0	91	2	1	2	0	0	1	115	77	54	7.6	7.4	7.5	108	12	1.2	218	79	42	157	5.5	0
2	67	20.6	81	1	1	1	0	0	0	111	78	84	8.4	8.9	8.65	87	10	0.63	210	51	59	141	5.4	1
1	66	25.5	90.5	1	1	2	0	0	0	122	83	60	7.6	7.5	7.55	87	16	1	233	193	57	139	5.4	0
1	55	27.6	99	2	2	1	1	0	1	135	92	71	10.6	11.2	10.9	118	16	0.95	268	60	57	201	6.1	1
1	65	26.1	90.5	1	1	1	1	0	0	132	78	56	7.4	7.4	7.4	93	19	1.04	213	39	69	128	5.4	0
2	55	20.2	72	2	2	2	1	0	0	143	88	53	7.5	7.4	7.45	96	6	0.68	178	133	79	75	5.5	0
1	52	25.3	89	2	2	2	1	0	1	136	91	50	7.4	7.1	7.25	89	13	0.78	204	209	38	123	5.3	1
1	59	24.3	95	2	1	1	1	0	0	126	86	62	7.3	7.8	7.55	107	13	0.84	214	89	57	135	5.7	0
1	57	32.2	104	2	2	2	1	0	1	139	92	61	7.1	7.2	7.15	92	14	1.04	224	75	42	166	5.7	0
2	68	20.2	85	1	1	1	1	0	0	137	92	83	11.3	10.1	10.7	103	16	0.83	213	131	66	106	5.4	0
1	53	26.4	95	1	1	1	1	0	1	122	95	71	8.7	12.5	10.6	90	14	0.97	253	73	64	163	5.5	1
1	64	26.7	89	2	2	2	1	0	1	143	96	73	10.6	8.9	9.75	109	16	1.04	186	69	64	108	5.9	0
2	57	22.3	81	1	1	2	0	0	0	123	88	78	7.1	7.3	7.2	106	11	0.68	210	142	56	125	6	0
2	74	24.2	88	1	1	2	1	1	1	103	61	52	24.8	26	25.4	159	29	0.97	262	162	51	160	7.5	1
2	57	21.5	81.5	1	1	2	1	0	1	173	115	70	8.3	8.3	8.3	97	19	0.75	251	71	81	149	5.7	1
